# Supplementary material for: Factors influencing the participation of surrogate decision-makers for advanced cancer patients in advance care planning: A cross-sectional study
Source: PLoS One. 2025 Jun 16;20(6):e0325551. doi: 10.1371/journal.pone.0325551 (PMC12169540; doi:10.1371/journal.pone.0325551)
Supplement: Attachment — Submitted filename: PONE-D-24-35495_reviewer comment.pdf [file pone.0325551.s003.pdf]

PLOS ONE

Factors influencing the participation of surrogate decision-makers for advanced cancer patients in advance care planning: A cross-sectional study

--Manuscript Draft--

Manuscript Number:	PONE-D-24-35495
Article Type:	Research Article
Full Title:	Factors influencing the participation of surrogate decision-makers for advanced cancer patients in advance care planning: A cross-sectional study
Short Title:	Influencing factors of participation in ACP
Corresponding Author:	Xiaoqin Ma<br>Zhejiang Chinese Medical University<br>Hangzhou, Zhejiang CHINA
Keywords:	Advance care planning, Advance directives, Surrogate decision-makers, Decision-making, Advanced cancer patients
Abstract:	Aim: This study explores the influencing factors of participation willingness of surrogate decision-makers of advanced cancer patients towards advance care planning. The aim is to provide reference for the construction of the education scheme of advance care planning, and then improve the participation of surrogate decision-makers towards advance care planning.<br>Design: A cross-sectional Study.<br>Method: A total of 276 surrogate decision-makers were conveniently sampled for this study. Through quantitative research, a questionnaire survey was conducted to the surrogate decision-makers of advanced cancer patients. Then perform statistical analysis to identify the factors influencing the participation of surrogate decision-makers in advance care planning for advanced cancer patients.<br>Results: Through investigation, this study found that the participants have a positive attitude towards advance care planning. Through univariate analysis, correlation analysis and multiple linear regression analysis, we found that seven variables have an impact on the participation attitudes of surrogate decision-makers in advanced cancer patients towards advance care planning.<br>Conclusion: Our results suggest that some interventions need to be implemented to strengthen the willingness of the surrogate decision-makers to participate in advance care planning. The social propaganda about advance care planning should be carried out, and the establishment of relevant legal provisions should also be paid attention to.<br>Impact: In mainland China, there is a need to increase the involvement of surrogate decision-makers of advanced cancer patients in ACP. This study can provide a basis for the development of future educational scheme. At the same time, it can also provide a basis for the formulation of relevant policies in mainland China.<br>Patient or public contribution: Surrogate decision-makers of advanced cancer patients were involved in the design of the study particularly in assessment of validation of the tools used in the study.
Order of Authors:	Zhihao Han
	Guiyue Ma
	Yong Fang
	Linxia Tang
	Xiaoqin Ma
Additional Information:	
Question	Response
Financial Disclosure<br><br>Enter a financial disclosure statement that describes the sources of funding for the	The author(s) received no specific funding for this work.

Powered by Editorial Manager® and ProduXion Manager® from Aries Systems Corporation

work included in this submission. Review the submission guidelines for detailed requirements. View published research articles from *PLOS ONE* for specific examples.<br><br>This statement is required for submission and **will appear in the published article** if the submission is accepted. Please make sure it is accurate.<br><br>**Funded studies**<br>Enter a statement with the following details:<br>• Initials of the authors who received each award<br>• Grant numbers awarded to each author<br>• The full name of each funder<br>• URL of each funder website<br>• Did the sponsors or funders play any role in the study design, data collection and analysis, decision to publish, or preparation of the manuscript?<br><br>Did you receive funding for this work?	
**Competing Interests**<br><br>Use the instructions below to enter a competing interest statement for this submission. On behalf of all authors, disclose any competing interests that could be perceived to bias this work—acknowledging all financial support and any other relevant financial or non-financial competing interests.<br><br>This statement is required for submission and **will appear in the published article** if the submission is accepted. Please make sure it is accurate and that any funding sources listed in your Funding Information later in the submission form are also declared in your Financial Disclosure statement.<br><br>View published research articles from *PLOS ONE* for specific examples.	The authors have declared that no competing interests exist.

**NO authors have competing interests**<br><br>Enter: *The authors have declared that no competing interests exist.*<br><br>**Authors with competing interests**<br><br>Enter competing interest details beginning with this statement:<br><br>*I have read the journal's policy and the authors of this manuscript have the following competing interests: [insert competing interests here]*<br><br>* typeset	
**Ethics Statement**<br><br>Enter an ethics statement for this submission. This statement is required if the study involved:<br><br>• Human participants<br>• Human specimens or tissue<br>• Vertebrate animals or cephalopods<br>• Vertebrate embryos or tissues<br>• Field research<br><br>Write "N/A" if the submission does not require an ethics statement.<br><br>General guidance is provided below. Consult the submission guidelines for detailed instructions. **Make sure that all information entered here is included in the Methods section of the manuscript.**	Ethical clearance was sought from Zhejiang Chinese Medical University ethics committee. Clearance to collect data was also sought from ethical committees in the study settings. Participation in the study was entirely voluntary; participants were provided written informed consent to participate and took part anonymously. Ethical approval number is 20230216-5, and the approval date is 16th February 2023.

Powered by Editorial Manager® and ProduXion Manager® from Aries Systems Corporation

**Format for specific study types**<br><br>**Human Subject Research (involving human participants and/or tissue)**<br>• Give the name of the institutional review board or ethics committee that approved the study<br>• Include the approval number and/or a statement indicating approval of this research<br>• Indicate the form of consent obtained (written/oral) or the reason that consent was not obtained (e.g. the data were analyzed anonymously)<br><br>**Animal Research (involving vertebrate animals, embryos or tissues)**<br>• Provide the name of the Institutional Animal Care and Use Committee (IACUC) or other relevant ethics board that reviewed the study protocol, and indicate whether they approved this research or granted a formal waiver of ethical approval<br>• Include an approval number if one was obtained<br>• If the study involved *non-human primates*, add *additional details* about animal welfare and steps taken to ameliorate suffering<br>• If anesthesia, euthanasia, or any kind of animal sacrifice is part of the study, include briefly which substances and/or methods were applied<br><br>**Field Research**<br><br>Include the following details if this study involves the collection of plant, animal, or other materials from a natural setting:<br>• Field permit number<br>• Name of the institution or relevant body that granted permission	
**Data Availability**<br><br>Authors are required to make all data underlying the findings described fully available, without restriction, and from the time of publication. PLOS allows rare exceptions to address legal and ethical concerns. See the PLOS Data Policy and FAQ for detailed information.	Yes - all data are fully available without restriction

Powered by Editorial Manager® and ProduXion Manager® from Aries Systems Corporation

A Data Availability Statement describing where the data can be found is required at submission. Your answers to this question constitute the Data Availability Statement and **will be published in the article**, if accepted.<br><br>**Important:** Stating 'data available on request from the author' is not sufficient. If your data are only available upon request, select 'No' for the first question and explain your exceptional situation in the text box.<br><br>Do the authors confirm that all data underlying the findings described in their manuscript are fully available without restriction?	
**Describe where the data may be found in full sentences. If you are copying our sample text, replace any instances of XXX with the appropriate details.**<br><br>• If the data are **held or will be held in a public repository**, include URLs, accession numbers or DOIs. If this information will only be available after acceptance, indicate this by ticking the box below. For example: *All XXX files are available from the XXX database (accession number(s) XXX, XXX.*).<br>• If the data are all contained **within the manuscript and/or Supporting Information files**, enter the following: *All relevant data are within the manuscript and its Supporting Information files.*<br>• If neither of these applies but you are able to provide **details of access elsewhere**, with or without limitations, please do so. For example:<br><br>*Data cannot be shared publicly because of [XXX]. Data are available from the XXX Institutional Data Access / Ethics Committee (contact via XXX) for researchers who meet the criteria for access to confidential data.*<br><br>*The data underlying the results presented in the study are available from (include the name of the third party*	All relevant data are within the manuscript and its Supporting Information files.

and contact information or URL).<br>• This text is appropriate if the data are owned by a third party and authors do not have permission to share the data.<br><br>* typeset	
Additional data availability information:	Tick here if the URLs/accession numbers/DOIs will be available only after acceptance of the manuscript for publication so that we can ensure their inclusion before publication.

Powered by Editorial Manager® and ProduXion Manager® from Aries Systems Corporation

Manuscript Click here to access/download;Manuscript;Manuscript.docx

Factors influencing the participation of surrogate decision-makers for advanced cancer patients in advance care planning: A cross-sectional study

Zhihao Han[1], Guiyue Ma[2], Yong Fang[3], Linxia Tang[2], Xiaoqin Ma[2*]

[1] Zhejiang Chinese Medical University First Clinical School of Medicine, Hangzhou City, Zhejiang Province, China

[2] Department of Nursing, Zhejiang Chinese Medical University, Hangzhou City, Zhejiang Province, China

[3] Department of Nursing, Changsha Medical College, Changsha City, Hunan Province, China

[*] Correspondence: 20041028@zcmu.edu.cn

Abstract

Aim: This study explores the influencing factors of participation willingness of surrogate decision-makers of advanced cancer patients towards advance care planning. The aim is to provide reference for the construction of the education scheme of advance care planning, and then improve the participation of surrogate decision-makers towards advance care planning.

Design: A cross-sectional Study.

Method: A total of 276 surrogate decision-makers were conveniently sampled for this study. Through quantitative research, a questionnaire survey was conducted to the surrogate decision-makers of advanced cancer patients. Then perform statistical analysis to identify the factors influencing the participation of surrogate decision-makers in advance care planning for advanced cancer patients.

Results: Through investigation, this study found that the participants have a positive attitude towards advance care planning. Through univariate analysis, correlation analysis and multiple linear regression analysis, we found that seven variables have an impact on the participation attitudes of surrogate decision-makers in advanced cancer patients towards advance care planning.

Conclusion: Our results suggest that some interventions need to be implemented to strengthen the willingness of the surrogate decision-makers to participate in advance care planning. The social propaganda about advance care planning should be carried out, and the establishment of relevant legal provisions should also be paid attention to.

Impact: In mainland China, there is a need to increase the involvement of surrogate decision-makers of advanced cancer patients in ACP. This study can provide a basis for the development of future educational scheme. At the same time, it can also provide a basis for the formulation of relevant policies in mainland China.

Patient or public contribution: Surrogate decision-makers of advanced cancer patients were involved in the design of the study particularly in assessment of validation of the tools used in the study.

Keywords: Advance care planning, Advance directives, Surrogate decision-makers, Decision-making, Advanced cancer patients

1 INTRODUCTION

Advance Care Planning (ACP) is defined as a process in which patients can discuss medical preferences with families and health care providers, which aims to respect the patient's values and ensure that the patient can receive reasonable medical care at the end of life [1]. The concept of ACP is similar to that of Advance directives (ADs). In terms of time, ACP is a process of reaching a consensus, and ADs is a time point for signing relevant documents [2]. In essence, ACP is the consensus reached through communication, and ADs is more normative in the implementation of specific treatment [2]. The implementation of ACP is guaranteed by laws in many countries and regions. Some countries, such as the United Kingdom, the United States, Australia, Singapore, etc., have issued relevant regulations on ACP to protect the rights and interests of citizens [3]. America passed the Patient Self-Determination Act to ensure the patient's right to execute the ADs [4]. The requirements and contents of the ADs are regulated by federal laws in Australia [5]. In 2000, Taiwan implemented the Hospice Palliative Care Act to respect the hospice right of terminally ill patients [6]. Current debates around future directions for ACP in the United States and internationally concentrate on professional and policy perspectives [7]. At present, the

discussion of ACP is mainly about feasibility and ethical aspects in mainland China, which leads to slow development of ACP.

2 BACKGROUND

Some reports pointed out that nearly half of all new cancer cases worldwide occurred in Asia and most of those individuals lived in China [8]. With the improvement of medical level, the survival time of patients with advanced cancer has been prolonged. However, there is a problem of general overtreatment [9], which is deeply influenced by traditional ideology in mainland Chian [10]. Scholars, researchers, clinicians, and citizens care about overtreatment (when they do) because it is taken to cause harm and/or waste precious healthcare resources [11]. The overtreatment will lead to the problems of wasting medical resources, reducing the life quality of patients and increasing the family's financial burden [12]. However, how to avoid overtreatment in advanced cancer patients? Some researchers believe that providing patients with multicriteria decision support can effectively avoid the occurrence of overtreatment [13]. Therefore, we need to know the medical preferences of patients, and provide better medical services on the premise of respecting their wishes. ACP is an important way to achieve this goal.

The formulation of ACP requires the participation of patients family members and health care providers. Studies have found that up to 70% of patients will need some or all of their end of life decisions to be made by a surrogate [14]. In mainland Chian, when patients lose their decision-making ability, their family member usually make medical decisions for patients as surrogate. As ACP interventions have been developed and tested, however, assessment has focused primarily on patients engagement with ACP, either by measuring ACP completion rates, or more recently, engagement in the comprehensive ACP process as measured by the ACP Engagement Survey [15-18]. However, surrogate decision maker also play an important role in the ACP process. Compared with health care providers, surrogate decision-makers are more accurate in replacing patients to transmit treatment preference [19]. The purpose of this study was to explore the influencing factors of the surrogate decision-makers participation in ACP.

3 THE STUDY

3.1 Aim

The aim of this study was to explore the influencing factors of their participation in ACP. And then to provide reference for the construction of the educational scheme of advance care planning to improve their participation.

4 METHODS

4.1 Participants and Data Collection

In mainland China, for serious diseases that are almost incurable, such as advanced cancer, most people will choose the treatment scheme of Traditional Chinese Medicine combined with western medicine for final maintenance treatment [20, 21]. Therefore, this study selected subjects from the First Affiliated Hospital of Zhejiang Chinese Medical University for the structured questionnaire survey. It is one of the largest hospitals of Zhejiang Province, in which oncology was rated as a key discipline by State Administration of Traditional Chinese Medicine of the People's Republic of China. The inclusion criteria of participants were: (1) age above 18 years old; (2) surrogate decision-makers are designated in writing by advanced cancer patients according to the relevant laws, and these surrogate decision-makers may be the patient's family members or friends; (3) consented to participate this study and signed an information letter and consent. The exclusion criteria of participants: (1) severe mental disorder or communication disorder; (2) not being well enough to complete the questionnaire. Data collection was conducted from February 20, 2023 to January 28, 2024.

According to Kendall's [22] sample size estimation principle, this study includes a total of 23 variables: 12 variables from demographic information characteristics, 1 dimension from life orientation test, 4 dimensions from uncertainty in illness scale-family member form, 3 dimensions from social support rating scale, and 3 dimensions from advance care planning engagement survey for surrogate decision-makers. Taking 5-10 times the number of observation variables, the sample size is estimated to be 115-230 cases. Meanwhile, considering a dropout rate of 20%, the final sample size range determined for this study is 138-276 cases.

4.2 Measurements

To identify the influencing factors of surrogate decision maker involvement in ACP in patients with advanced cancer, a structured questionnaire was designed in this study. The structured questionnaire were divided into five categories. Looking back at previous studies on influencing factors, interpretations have often been made from the individual, environmental, and behavioral dimensions. The theory of triadic reciprocal determinism proposes a dynamic relationship model among individuals, environment, and behavior [23]. Therefore, this study, based on the theory of triadic reciprocal determinism, explores how individual and environmental factors influence the decision-making behavior of surrogate decision-makers participating in ACP. Individual factors such as self-efficacy and outcome expectations mainly manifest as life orientation ability and uncertainty about illness. Environmental factors primarily manifest as the level of social support for surrogate decision-makers, and behavior involves the evaluation of surrogate decision-makers' participation in ACP.

4.2.1 Demographic information of the characteristics

The demographic information sheet was designed by the researchers. Previous studies were used to determined potential demographic variables that may affect surrogate decision-makers' attitudes. Eventually, 12 variables were considered, age, gender, education level, marital status, family numbers, annual income, relationship with patients, classification of patients' cancer, treatment expenditure, patients' self-care level, medical decision-making experiences and awareness of ACP.

4.2.2 Advance care planning engagement survey for surrogate decision-makers

The questionnaire was compiled by Wang Tianhang et al. [24] and includes 3 dimensions: conditions, thinking, and readiness to become a surrogate decision maker, with a total of 17 items. It uses a Likert 5-point rating scale. The response options range from 1, indicating "not at all / never," to 5, indicating "means very much / always," with 2 representing "means hardly ever / rarely," 3 as "means somewhat / sometimes," and 4 as "often / frequently." The score range of 17 to 85. A higher score indicates a higher level of involvement of advanced cancer patients as surrogate decision-makers in ACP. The total questionnaire's Cronbach's alpha value is 0.923 in this study, affirming the scale's reliability for the research purposes.

4.2.3 Uncertainty in illness scale-family member form

This scale was developed by Wenying Wang [25] and is mainly used to evaluate the level of disease uncertainty in the family members of patients. The scale consists of 4 dimensions: namely ambiguity, lack of clarification, lack of information, and unpredictability, with a total of 25 items. It uses a 5-point Likert scale ranging from 1 (strongly disagree) to 5 (strongly agree). Items 6, 9, 17, 21, 23, 24, and 25 in the scale are reverse scored, while the remaining items are positively scored, with a total score range of 25 to 125. A higher score indicates a higher level of disease uncertainty in family members. In this study, the total scale's Cronbach's alpha value is 0.864.

4.2.4 Social support rating scale

This scale, designed by Shuiyuan Xiao [26], is used to assess an individual's level of social support. The scale consists of 3 dimensions: subjective social support, objective social support, and social support utilization. Items 1, 2, 3, 4, 8, 9, and 10 in the scale are single-choice questions, with options 1-4 scored 1-4 points respectively. Item 5 has 5 options (A, B, C, D, E) with scores ranging from 1-4 for "none" to "full support." For items 6 and 7, if "no source at all" is selected, 0 points are given; if "from the following sources" is chosen, each source selected is scored from 0-9 points. The score range of this scale is 12-66 points, with higher scores indicating higher levels of social support. The total scale's Cronbach's alpha value is 0.734 in this study.

4.2.5 Life orientation test

This questionnaire was compiled by Wengen Deng [27] and is mainly used to assess an individual's level of optimism, generally applicable to Chinese people. This scale is a single-dimensional self-assessment scale consisting of 6 items. The questionnaire uses a Likert 5-point rating scale from 0 (strongly disagree) to 4 (strongly agree). Items 1, 3, and 6 are scored positively; items 2, 4, and 5 are scored negatively. The total score on this questionnaire ranges from 0-24 points, with higher scores indicating a higher level of optimism in the individual. In this study, the total questionnaire's Cronbach's alpha value is 0.780.

4.3 Statistical Analysis

Statistical analyses were performed using IBM SPSS Statastics 26.0 software. Descriptive statistics were used to describe surrogate decision-makers characteristics. Univariate analysis is applied to understand the relationship between basic

information of surrogate decision-makers and participation towards ACP. Using correlation analysis to explore the correlation between the uncertainty of disease, the social support, the optimism and participation towards ACP. The variables with statistical significance in univariate analysis and correlation analysis are included as independent variables in multiple linear regression analysis to control and identify confounding factors and explore the influencing factors. A value of alpha of 0.05 was used for all statistical tests.

4.4 Ethical considerations

Ethical clearance was sought from Zhejiang Chinese Medical University ethics committee. Clearance to collect data was also sought from ethical committees in the study settings. Participation in the study was entirely voluntary; participants were provided written informed consent to participate and took part anonymously. Ethical approval number is 20230216-5, and the approval date is 16th February 2023.

5 RESULTS

5.1 Characteristics of the participants

We interviewed a total of 276 participants. The participants' characteristics and advanced cancer patients' characteristics are presented in Table 1. The participants were evenly distributed by age and gender. Most of the participants were married and lived with their families. Most of the participants had no experiences in medical dicision-making and lacked knowledge of ACP. 44.9% of advanced cancer patients are parents of the participants, and 43.8% of advanced cancer patients are spouses of the participants. Most patients with advanced cancer can take care of themselves in daily life.

Table 1 Characteristics of the participants

Characteristics	Sample size (n)	Percentage (%)
**Age (years)**		
≤40	98	35.5
41-60	113	40.9
≥61	65	23.6
**Gender**		

Female	144	52.2
Male	132	47.8
**Education level**		
Primary school or less	46	16.7
Junior school	72	26.1
Senior high school	57	20.7
College school	90	32.6
Master degree or higher	11	4.0
**Marital status**		
Single, Bereaved, Divorced	37	13.4
Married	239	86.6
**Medical decision-making experiences**		
No	194	70.3
Yes	82	29.7
**Awareness of ACP**		
Unaware	189	68.5
Heard of ACP	55	19.9
Partial aware	28	10.1
Aware	4	1.4
**Treatment expenditure (RMB 10, 000 yuan)**		
≤10	110	39.9
11-30	120	43.5
31-50	28	10.1
≥51	18	6.5
**Annual income (RMB 10, 000 yuan)**		
≤10	112	40.6
11-30	101	36.6
31-50	30	10.9
≥51	33	12.0
**Famliy numbers**		
≤3	110	39.9
4-5	121	43.8
≥6	45	16.3

**Relationship with patient**		
Parents	124	44.9
Children	19	6.9
Couples	121	43.8
Brothers and sisters	6	2.2
Grandparents	6	2.2
**Classification of patients' cancer**		
Digestive system tumor	112	40.6
Blood system tumor	34	12.3
Urogenital system tumor	28	10.1
Head and neck tumor	25	9.1
Chest tumor	77	27.9
**Patients' self-care level**		
No self-care ability	24	8.7
Part of daily life self-care ability	77	27.9
Daily life self-care ability	62	22.5
Complete self-care ability	113	40.9

5.2 Preliminary screening of factors influencing ACP participation

Age, education level, annual income and other characteristics were influencing factors of participants' attitudes towards ACP. No differences in marital status, family numbers, patient's tumor type and patient's self-care ability were observed with different attitudes (Table 2). In addition, the correlation between the uncertainty of disease, the social support, the optimism with participants' attitudes towards ACP were shown in Table 3.

Table 2 Characteristics associated with participants' attitudes towards ACP

Characteristics	Conditions	Consideration	Readiness	Attitudes
**Age (years)**				
≤40	25.10±3.63	11.00 (7.00)	19.00 (7.00)	55.50 (18.00)
41-60	23.38±5.25	10.00 (7.00)	17.00 (7.00)	49.00 (21.00)

≥61	21.46±5.76	7.00 (8.00)	14.00 (7.00)	43.00 (24.00)
F/H	10.996	5.605	16.536	16.604
P	<0.001	0.061	<0.001	<0.001
**Gender**				
Female	22.51±4.93	9.00 (7.00)	16.00 (8.00)	47.50 (19.00)
Male	24.67±4.96	10.00 (8.00)	17.50 (9.00)	54.00 (22.00)
t/Z	-3.627	-0.557	-2.478	-2.678
P	<0.001	0.577	0.013	0.007
**Education level**				
Primary school or less	18.85±4.77	7.00 (2.00)	13.00 (3.00)	40.74±9.02
Junior school	21.38±4.64	7.00 (4.00)	15.00 (5.00)	45.33±10.73
Senior high school	24.93±3.63	12.00 (6.00)	19.05±4.38	55.44±10.61
College school	27.00 (5.00)	13.00 (7.00)	20.00 (8.00)	57.83±10.55
Master degree or higher	29.00 (2.00)	16.00 (5.00)	22.36±3.53	65.27±8.75
F/H	92.646	59.416	77.940	34.457
P	<0.001	<0.001	<0.001	<0.001
**Marital status**				
Single, Bereaved, Divorced	23.11±4.60	10.19±3.95	17.00 (9.00)	48.00 (22.00)
Married	24.00 (8.00)	10.00 (8.00)	17.00 (7.00)	49.00 (21.00)
Z	-0.831	-0.161	-0.284	-0.311
P	0.406	0.872	0.776	0.756
**Medical decision-making experiences**				
No	23.00 (6.00)	8.00 (6.00)	15.00 (6.00)	46.00 (15.00)
Yes	28.00 (4.00)	14.00 (5.00)	21.00±4.45	64.00 (12.00)
Z	-7.346	-6.780	-7.294	-7.852
P	<0.001	<0.001	<0.001	<0.001
**Awareness of ACP**				

Unaware	21.90±4.78	8.00 (6.00)	15.90±4.13	46.00 (15.00)
Heard of ACP	28.00 (3.00)	14.00 (5.00)	21.25±3.94	64.00 (11.00)
Partial aware	27.36±3.47	13.61±3.34	22.14±4.08	63.11±9.15
Aware	26.50±4.93	10.75±4.27	17.50±5.80	54.75±14.18
F/H	70.245	59.818	36.639	82.403
P	<0.001	<0.001	<0.001	<0.001
**Treatment expenditure (RMB 10, 000 yuan)**				
≤10	22.40±4.52	7.00 (6.00)	15.00 (5.00)	43.00 (14.00)
11-30	25.00 (7.00)	11.50 (7.00)	18.50 (8.00)	53.72±12.98
31-50	25.50 (7.00)	12.00 (7.00)	19.18±4.91	54.36±11.71
≥51	28.50 (5.00)	12.17±3.20	20.56±4.25	63.00 (12.00)
H	20.812	28.671	27.781	28.729
P	<0.001	<0.001	<0.001	<0.001
**Annual income (RMB 10, 000 yuan)**				
≤10	21.37±4.94	7.50（6.00）	14.50 (5.00)	43.50 (16.00)
11-30	24.00 (6.00)	11.00 (7.00)	18.00 (8.00)	52.00 (21.00)
31-50	27.50 (5.00)	12.50 (6.00)	18.40±4.60	55.70±12.45
≥51	27.00 (3.00)	12.09±3.31	20.52±3.95	58.97±8.96
H	42.865	23.014	37.549	40.237
P	<0.001	<0.001	<0.001	<0.001
**Famliy numbers**				
≤3	24.50 (8.00)	11.50 (8.00)	18.00 (9.00)	53.50 (24.00)
4-5	23.00 (7.00)	9.00 (7.00)	16.00 (7.00)	47.00 (19.00)
≥6	24.16±3.93	8.00 (8.00)	16.00 (7.00)	48.00 (20.00)
H	4.995	5.366	4.744	5.756
P	0.082	0.068	0.093	0.056
**Relationship with**				

**patient**				
Parents	24.69±4.15	11.00 (7.00)	18.00 (7.00)	54.00 (19.00)
Children	21.37±4.11	7.74±2.88	13.00 (4.00)	44.21±9.38
Couples	22.55±5.77	9.00 (8.00)	15.00 (8.00)	47.00 (24.00)
Brothers and sisters	26.17±2.93	10.50±4.46	18.83±4.79	55.50±11.36
Grandparents	23.83±5.19	16.00 (8.00)	19.33±4.80	56.50±12.36
F/H	4.253	11.811	16.576	15.189
P	0.002	0.019	0.002	0.004
**Classification of patients' cancer**				
Digestive system tumor	23.00 (8.00)	10.00 (8.00)	17.00 (8.00)	51.38±12.55
Blood system tumor	24.53±3.59	9.49±3.78	16.00 (7.00)	49.00 (19.00)
Urogenital system tumor	23.64±4.65	10.50 (9.00)	17.00 (11.00)	52.00 (27.00)
Head and neck tumor	23.64±5.83	8.00 (9.00)	16.36±4.89	46.00 (27.00)
Chest tumor	24.00 (9.00)	10.00 (7.00)	17.00 (9.00)	51.00 (24.00)
H	1.371	2.139	2.301	0.697
P	0.849	0.710	0.681	0.952
**Patients' self-care level**				
No self-care ability	25.50 (14.00)	12.50 (10.00)	17.46±5.70	51.25±16.50
Part of daily life self-care ability	26.00 (9.00)	12.00 (8.00)	20.00 (10.00)	60.00 (23.00)
Daily life self-care ability	23.42±4.74	10.00 (5.00)	16.00 (8.00)	48.00 (19.00)
Complete self-care ability	23.00 (6.00)	8.00 (7.00)	16.00 (6.00)	47.00 (15.00)
H	4.265	8.211	9.602	7.006
P	0.234	0.042	0.022	0.072

Table 3 The correlation between the uncertainty of disease, the social support, the optimism with participants' attitudes towards ACP

Characteristics	Conditions	Consideration	Readiness	Attitudes
The uncertainty of disease	-0.485**	-0.452**	-0.469**	-0.524**
The social support	0.514**	0.410**	0.501**	0.534**
The optimism	0.356**	0.387**	0.401**	0.424**

**: P<0.01

5.3 Factors influencing participation in ACP

The scores of ACP engagement survey for surrogate decision-makers as the dependent variable, and variables with $P<0.05$ in the results of univariate analysis and correlation analysis as independent variables, multiple linear regression analysis was carried out, and variables were screened by stepwise regression method. The assignment of independent variables is shown in Table 4. A total of 11 influencing factors entered the model, and the tolerance (TOL) of each variable in the fitting model was greater than 0.1, and the variance expansion factor (VIF) was less than 5. It was considered that there was no obvious multicollinearity between each variable.

Table 4 Independent variable assignment in multiple linear regression analysis

Independent variable	Assignment
Age	1=≤40, 2=40-60, 3=≥61
Gender	1=Female, 2=Male
Education level	1= Primary school or less, 2= Junior school, 3= Senior high school, 4= College school, 5= Master degree or higher
Medical decision-making experiences	1=No, 2=Yes
Awareness of ACP	1= Unaware, 2= Heard of ACP, 3= Partial aware, 4= Aware
Treatment expenditure	1=≤10, 2=11-30, 3=31-50, 4=≥51
Annual income	1=≤10, 2=11-30, 3=31-50, 4=≥51
Relationship with patient	1= Parents, 2= Children, 3= Couples, 4= Brothers and sisters, 5= Grandparents

The uncertainty of disease	null
The social support	null
The optimism	null

The multiple linear regression analysis results show that seven variables were included in the regression equation. the medical decision-making experiences, the education level, the awareness of ACP and other influencing factors affect the participants' attitudes towards ACP, which explains 56.7% of the total variation of the equation. The integrity test of the regression model has statistical significance (Adjusted R^2=0.567; F=52.448; P＜0.001). It is worth noting that participants' age, gender, annual income, and relationship with patient were found to have an impact on surrogate decision-makers' participation in ACP in the univariate analysis results, but the multiple linear regression model showed that these four variables do not have an impact on surrogate decision-makers' participation in ACP. Multiple linear regression analyses to predict attitudes of participants towards ACP are presented in Table 5.

Table 5 Multiple linear regression analysis of independent predictors of attitudes towards ACP

**Characteristics**	**Regression coefficient**	**Standardized coefficient**	**t**	**P**
Constant	29.912	-	7.040	＜0.001
Medical decision-making experiences	6.144	0.224	4.934	＜0.001
Education level	2.460	0.230	4.650	＜0.001
Awareness of ACP	1.989	0.116	2.387	0.018
Treatment expenditure	1.541	0.105	2.554	0.011
The uncertainty of disease	-0.196	-0.232	-5.051	＜0.001
The social support	0.249	0.159	3.186	0.002
The optimism	0.274	0.105	2.279	0.023

6 DISCUSSION

This study provides valuable insights into the attitudes of surrogate decision-makers of Chinese advanced cancer patients towards ACP, especially given the limited research in mainland China. A total of 276 surrogate decision-makers of advanced cancer patients participated in this study, of which 52.2% were women and 47.8% were men, with a balanced gender distribution. In the relationship between surrogate decision-makers and advanced cancer patients, the relationship between parents and couples accounts for 44.9% and 43.8% respectively. Combined with the age distribution and the proportion of married people in marital status (68.6%), this means that the risk of cancer among young people is greatly increasing. It is consistent with previous research conclusions [28-30]. Among the participants, those younger than 60 years old account for a large proportion. First of all, it is related to the high incidence of cancer in the elderly, which leads their children to play the role of surrogate decision-makers. Secondly, it is the younger onset of cancer that causes couples to act as surrogate decision maker for each other. Relevant research shows that education level has an impact on cognition, and the higher the education level, the better the individual’s cognitive ability [31]. The results of this study show that 36.6% of the participants’ education level is above college school, and 31.4% of them have Partial awareness of ACP. The similar proportion confirms the correlation between education level and cognition to some extent [31].

70.3% of the participants have not medical decision-making experiences, which is mainly due to the limited implementation of the doctor-patient-surrogate decision-making model in mainland China, which leads to the inability of the surrogate decision-makers to fully obtain the patient’s medical information [32-34]. The results of this study show that the treatment expenditure and annual income have a similar composition structure, which indicates that some participants may spend most of their income on the treatment expenditure of patients with advanced cancer, which also means that the treatment of patients has brought huge economic burden to their families [35-37]. Among the cancer types, digestive system tumors accounted for the highest proportion (40.6%), followed by chest tumors (27.9%). The digestive system tumors are mainly gastric cancer and colorectal cancer, and the chest tumors are mainly lung cancer and esophageal cancer. This conclusion is partly different from the official statistics of China [38], which may be related to the inconsistency of the

time to collect questionnaires in different departments. It may also be related to the difference in the number of cases in different departments.

Multiple linear regression analysis reveals that seven factors will affect surrogate decision-maker's participation in ACP. Surrogate decision-makers with decision-making experiences will be more calm in the face of family members' medical decisions, which stems from a more objective understanding of the development of the disease process [39]. Decision aid tools [40] can make the surrogate decision-makers obtain medical information in the form of words, pictures and videos, and help them to formulate treatment plans in line with patients' interests and values, which can guide the surrogate decision-makers to generate reasonable expectations for treatment results and reduce medical tangle [41]. Participants with higher education level have a more positive attitude towards ACP than those with lower education level, which is consistent with the research conclusions of Keam et al [41]. Participants with high education level have a strong sense of autonomy, and will think about life values and future medical wishes when actively seeking disease information [42, 43]. However, participants with low education level may lack appropriate knowledge to make them think, although they want to express their wishes [44]. Participants who have awareness of ACP have a more positive attitude than those who do not, which is consistent with the research conclusions of Park et al [45]. A full understanding of new things is a prerequisite for participation. The results show that the proportion of participants who understand ACP is low, which requires the government, media and hospitals to strengthen the publicity of ACP and strengthen the public's understanding of ACP [46].

This study believes that the treatment expenditure is an important factor considered by the surrogate decision-makers when making medical decisions for advanced cancer patients, and the high cost of treatment expenditure often puts surrogate decision-makers under great economic pressure [47, 48]. It is recommended that medical staff should understand the treatment needs and economic status of surrogate decision-makers and patients, help them weigh the pros and cons of different treatment options to provide personalized medical decision-making recommendations [49]. The uncertainty of disease affects participants' attitudes towards ACP. The uncertainty in illness theory holds that uncertainty arises when individuals are unable to establish an adaptive cognitive framework for related events [50]. For non-medical professional

surrogate decision-makers, it is necessary to increase communication with doctors to make them understand disease information more accurately. The social support affects participants' attitudes towards ACP. Social support refers to the material and spiritual support that individuals obtain from family or other social networks [51, 52]. The social support is associated with the attitude towards taking an active role in medical decision-making [53], and active medical decision-making can promote individuals to complete high-quality ACP [54]. Many research conclusions have shown that when individuals are in a good emotional state, they will produce more altruistic behavior [55-57]. Optimistic surrogate decision-makers can face patients with better attitudes and actively seek help from others to find solutions to problems when they encounter difficulties. Therefore, the optimistic surrogate decision-makers are more willing to participate in the discussion of patients' treatment decisions and have a more positive attitude towards ACP.

7 CONCLUSIONS

In mainland China, some interventions need to be implemented to strengthen the willingness of the surrogate decision-makers to participate in ACP. The most important measure is to strengthen the propaganda of ACP and avoid the public's cognitive errors on ACP. In addition, Some legal provisions must be established to protect the medical decisions of surrogate decision-makers and patients. At the same time, it is also the occupational protection for medical staff. Through this research, we have identified the factors influencing the engagement of surrogate decision-makers in ACP, providing a basis for the development of later educational programs and the establishment of relevant systems.

There are several limitations of this study. First, we used a convenience sample of participants in a single cancer center. Thus, their views may not represent the general population of surrogate decision-makers in mainland China. Second, We did not conduct qualitative interviews with surrogate decision-makers from an individual perspective to explore the influencing factors of individual-level participation in ACP, such as individual life views and values. Therefore, the conclusions of this study should be interpreted with caution. Next we will conduct additional research with a large national sample of surrogate decision-makers in mainland China. On this basis,

we will also carry out mixed research to deeply understand the attitudes of surrogate decision-makers towards ACP.

AUTHER CONTRIBUTIONS

Zhihao Han took charge of sampling, data analysis and manuscript writing. Guiyue Ma, Linxia Tang and Yong Fang assisted with the survey and data analyses. Xiaoqin Ma was responsible for the whole framework of this work. The authors declare no conflict of interest.

COI STATEMENT

No conflict of interest has been declared by the authors.

FUNDING INFORMATION

This project did not receive any specific grant from funding agencies in the public, commercial, or not-for-profit sectors.

ACKNOWLEDGMENTS

This research was not funded by any public, commercial, or nonprofit agency. No competing financial interests exist. The authors thank all members of the cancer center for their help in administering the questionnaire interview.

DATA AVAILABILITY STATEMENT

Data available on request from the authors.

REFERENCES

[1] C. Sedini, M. Biotto, L.M. Crespi Bel'skij, R.E. Moroni Grandini, M. Cesari, Advance care planning and advance directives: an overview of the main critical issues, Aging clinical and experimental research, 34 (2022) 325-330.

[2] P.A. Komesaroff, Advance Care Planning and Advance Care Directives: ideas whose time has passed?, Internal medicine journal, 47 (2017) 359-360.

[3] A. Canny, B. Mason, K. Boyd, Public perceptions of advance care planning (ACP) from an international perspective: a scoping review, BMC Palliat Care, 22 (2023) 107.

[4] A. Yu, Advance Care Planning Preferences in Hong Kong: A Cross-Sectional Study in a Community, Healthcare (Basel, Switzerland), 10 (2022).

[5] K.A. Kierner, B. Hladschik-Kermer, V. Gartner, H.H. Watzke, Attitudes of patients with malignancies towards completion of advance directives, Supportive care in cancer : official journal of the Multinational Association of Supportive Care in Cancer, 18 (2010) 367-372.
[6] H.C. Cheng, L.S. Ke, S.Y. Chang, H.Y. Huang, Y.C. Ku, M.J. Lee, Knowledge, attitudes, and behavioral intentions of elderly individuals regarding advance care planning: Questionnaire development and testing, PloS one, 17 (2022) e0272351.
[7] R.S. Morrison, D.E. Meier, R.M. Arnold, What's Wrong With Advance Care Planning?, Jama, 326 (2021) 1575-1576.
[8] S. McGuire, World Cancer Report 2014. Geneva, Switzerland: World Health Organization, International Agency for Research on Cancer, WHO Press, 2015, Advances in nutrition (Bethesda, Md.), 7 (2016) 418-419.
[9] X. Liang, C. Zhang, X. Ye, Overdiagnosis and overtreatment of ground-glass nodule-like lung cancer, Asia-Pacific journal of clinical oncology, (2024).
[10] X. Chen, R. Fan, The family and harmonious medical decision making: cherishing an appropriate Confucian moral balance, The Journal of medicine and philosophy, 35 (2010) 573-586.
[11] S.M. Carter, Overdiagnosis: An Important Issue That Demands Rigour and Precision Comment on "Medicalisation and Overdiagnosis: What Society Does to Medicine", Int J Health Policy Manag, 6 (2017) 611-613.
[12] J.R. Bhatt, L. Klotz, Overtreatment in cancer - is it a problem?, Expert opinion on pharmacotherapy, 17 (2016) 1-5.
[13] V.K. Rajput, J. Dowie, M.K. Kaltoft, Multicriteria Decision Support Would Avoid Overdiagnosis and Overtreatment, Stud Health Technol Inform, 275 (2020) 172-176.
[14] L.J. Van Scoy, A.G. Day, M. Howard, R. Sudore, D.K. Heyland, Adaptation and Preliminary Validation of the Advance Care Planning Engagement Survey for Surrogate Decision Makers, J Pain Symptom Manage, 57 (2019) 980-988.e989.
[15] G. Bravo, M.F. Dubois, B. Wagneur, Assessing the effectiveness of interventions to promote advance directives among older adults: a systematic review and multi-level analysis, Soc Sci Med, 67 (2008) 1122-1132.
[16] M.I. Tamayo-Velázquez, P. Simón-Lorda, R. Villegas-Portero, C. Higueras-Callejón, J.F. García-Gutiérrez, F. Martínez-Pecino, I.M. Barrio-Cantalejo,

Interventions to promote the use of advance directives: an overview of systematic reviews, Patient Educ Couns, 80 (2010) 10-20.
[17] R.L. Sudore, D.K. Heyland, D.E. Barnes, M. Howard, K. Fassbender, C.A. Robinson, J. Boscardin, J.J. You, Measuring Advance Care Planning: Optimizing the Advance Care Planning Engagement Survey, J Pain Symptom Manage, 53 (2017) 669-681.e668.
[18] R.L. Sudore, A.L. Stewart, S.J. Knight, R.D. McMahan, M. Feuz, Y. Miao, D.E. Barnes, Development and validation of a questionnaire to detect behavior change in multiple advance care planning behaviors, PLoS One, 8 (2013) e72465.
[19] E.K. Vig, H. Starks, J.S. Taylor, E.K. Hopley, K. Fryer-Edwards, Surviving surrogate decision-making: what helps and hampers the experience of making medical decisions for others, Journal of general internal medicine, 22 (2007) 1274-1279.
[20] Y. Xiang, Z. Guo, P. Zhu, J. Chen, Y. Huang, Traditional Chinese medicine as a cancer treatment: Modern perspectives of ancient but advanced science, Cancer medicine, 8 (2019) 1958-1975.
[21] X. Zhang, H. Qiu, C. Li, P. Cai, F. Qi, The positive role of traditional Chinese medicine as an adjunctive therapy for cancer, Bioscience trends, 15 (2021) 283-298.
[22] S. Chen, A. Ghadami, B.I. Epureanu, Practical guide to using Kendall's τ in the context of forecasting critical transitions, R Soc Open Sci, 9 (2022) 211346.
[23] A. Bandura, Social cognitive theory: an agentic perspective, Annu Rev Psychol, 52 (2001) 1-26.
[24] Tianhang. Wang, Wen. Wang, Wenting. Shen, Baoxin. Shi, Participation in advance care planning and associated factors among surrogate decision makers of patients with hematologic walignancies, Chinese general practice, 25 (2022) 859-866.
[25] Wenying. Wang, Shen Wang, Dasheng. Xia, Guipu. Cui, Chunmei. Wang, Study on reliability and validity of Chinese version of Mishel uncertainty in illness scale-family member form, Chinese nursing research, 26 (2012) 662-663.
[26] Suiyuan. Xiao, Theory and research applications of the Social Support Rating Scale, Journal of clinical psychiatry, (1994) 98-100.
[27] Wengen. Deng, Peng. Wang, Shuangshuang. Liu, Examining the issues and solutions for testing the dimensions of LOT and LOT-R using traditional methods, Journal of Shandong Normal University (Natural Science Edition), 24 (2009) 51-54.

[28] C. Wright, J. Heron, R. Kipping, M. Hickman, R. Campbell, R.M. Martin, Young adult cancer risk behaviours originate in adolescence: a longitudinal analysis using ALSPAC, a UK birth cohort study, BMC cancer, 21 (2021) 365.
[29] R.S. Zheng, S.W. Zhang, K.X. Sun, R. Chen, S.M. Wang, L. Li, H.M. Zeng, W.W. Wei, J. He, [Cancer statistics in China, 2016], Zhonghua zhong liu za zhi [Chinese journal of oncology], 45 (2023) 212-220.
[30] W. Chen, C. Xia, R. Zheng, M. Zhou, C. Lin, H. Zeng, S. Zhang, L. Wang, Z. Yang, K. Sun, H. Li, M.D. Brown, F. Islami, F. Bray, A. Jemal, J. He, Disparities by province, age, and sex in site-specific cancer burden attributable to 23 potentially modifiable risk factors in China: a comparative risk assessment, The Lancet. Global health, 7 (2019) e257-e269.
[31] M. Lövdén, L. Fratiglioni, M.M. Glymour, U. Lindenberger, E.M. Tucker-Drob, Education and Cognitive Functioning Across the Life Span, Psychological science in the public interest : a journal of the American Psychological Society, 21 (2020) 6-41.
[32] C. Chen, G. Cheng, X. Chen, L. Yu, Information disclosure to cancer patients in Mainland China: A meta-analysis, Psycho-oncology, 32 (2023) 342-355.
[33] J. Hahne, T. Liang, K. Khoshnood, X. Wang, X. Li, Breaking bad news about cancer in China: Concerns and conflicts faced by doctors deciding whether to inform patients, Patient education and counseling, 103 (2020) 286-291.
[34] H. Xu, M. Yuan, Family roles in informed consent from the perspective of young Chinese doctors: a questionnaire study, BMC medical ethics, 25 (2024) 2.
[35] J. Xu, J. Wang, R. Liu, J. Xing, L. Su, F. Yu, M. Lu, Mental health inpatient treatment expenditure trends in China, 2005-2012: evidence from Shandong, The journal of mental health policy and economics, 17 (2014) 173-182.
[36] A. Yao, X. Shen, J. Chai, J. Cheng, R. Liu, R. Feng, D. Wang, Characteristics and implications of insurance-reimbursed inpatient care for gastric and oesophageal cancers in Anhui, China, International health, 13 (2021) 446-455.
[37] W. Fu, J. Shi, X. Zhang, C. Liu, C. Sun, Y. Du, H. Wang, C. Liu, L. Lan, M. Zhao, L. Yang, B. Bao, S. Cao, Y. Zhang, D. Wang, N. Li, W. Chen, M. Dai, G. Liu, J. He, Effects of cancer treatment on household impoverishment: a multicentre cross-sectional study in China, BMJ open, 11 (2021) e044322.
[38] C. Maomao, L. He, S. Dianqin, H. Siyi, Y. Xinxin, Y. Fan, Z. Shaoli, X. Changfa, L. Lin, P. Ji, C. Wanqing, Current cancer burden in China: epidemiology, etiology, and prevention, Cancer biology & medicine, 19 (2022) 1121-1138.

[39] B.M. Bakke, M.A. Feuz, R.D. McMahan, D.E. Barnes, B. Li, A.M. Volow, J. Powell, R.L. Sudore, Surrogate Decision Makers Need Better Preparation for Their Role: Advice from Experienced Surrogates, Journal of palliative medicine, 25 (2022) 857-863.
[40] A. Emmanuel, Assisting clinical decision making: tools, rules and teams, Clinical medicine (London, England), 21 (2021) 81.
[41] B. Keam, Y.H. Yun, D.S. Heo, B.W. Park, C.H. Cho, S. Kim, D.H. Lee, S.N. Lee, E.S. Lee, J.H. Kang, S.Y. Kim, J.L. Lee, C.G. Lee, Y.K. Lim, S.Y. Kim, J.S. Choi, H.S. Jeong, M. Chun, The attitudes of Korean cancer patients, family caregivers, oncologists, and members of the general public toward advance directives, Supportive care in cancer : official journal of the Multinational Association of Supportive Care in Cancer, 21 (2013) 1437-1444.
[42] F. Wu, Y. Zhuang, X. Chen, H. Wen, W. Tao, Y. Lao, H. Zhou, Decision-making among the substitute decision makers in intensive care units: An investigation of decision control preferences and decisional conflicts, Journal of advanced nursing, 76 (2020) 2323-2335.
[43] F.P. Hopp, Preferences for surrogate decision makers, informal communication, and advance directives among community-dwelling elders: results from a national study, The Gerontologist, 40 (2000) 449-457.
[44] J.P. Rwabihama, J. Belmin, R. Rakotoarisoa, M. Hagege, E. Audureau, H. Benzengli, G. Ambime, M.T. Rabus, S. Bastuji-Garin, E. Paillaud, N. Oubaya, Promoting patients' rights at the end of life in a geriatric setting in France: The healthcare professionals' level of knowledge about surrogate decision-makers and advance directives, Patient education and counseling, 103 (2020) 1390-1398.
[45] H.Y. Park, Y.A. Kim, J.A. Sim, J. Lee, H. Ryu, J.L. Lee, C.H. Maeng, J.H. Kwon, Y.J. Kim, E.M. Nam, H.J. Shim, E.K. Song, K.H. Jung, E.J. Kang, J.H. Kang, Y.H. Yun, Attitudes of the General Public, Cancer Patients, Family Caregivers, and Physicians Toward Advance Care Planning: A Nationwide Survey Before the Enforcement of the Life-Sustaining Treatment Decision-Making Act, Journal of pain and symptom management, 57 (2019) 774-782.
[46] S.S. Lin Goh, Singapore Takes Six Steps Forward in 'The Quality of Death Index' Rankings, Asia-Pacific journal of oncology nursing, 5 (2018) 21-25.

[47] W. Jeong, S. Kim, H. Kim, E.C. Park, S.I. Jang, Effects of life-sustaining treatment plans on healthcare expenditure and healthcare utilization, BMC health services research, 23 (2023) 1236.
[48] C.J. Longo, M.I. Fitch, J.M. Loree, L.E. Carlson, D. Turner, W.Y. Cheung, D. Gopaul, J. Ellis, J. Ringash, M. Mathews, J. Wright, C. Stevens, D. D'Souza, R. Urquhart, T. Maity, F. Balderrama, E. Haddad, Patient and family financial burden associated with cancer treatment in Canada: a national study, Supportive care in cancer : official journal of the Multinational Association of Supportive Care in Cancer, 29 (2021) 3377-3386.
[49] L. van den Heuvel, M.J. Meinders, B. Post, B.R. Bloem, A.M. Stiggelbout, Personalizing decision-making for persons with Parkinson's disease: where do we stand and what to improve?, Journal of neurology, 269 (2022) 3569-3578.
[50] M.H. Mishel, Reconceptualization of the uncertainty in illness theory, Image--the journal of nursing scholarship, 22 (1990) 256-262.
[51] Y.Y. Usta, Importance of social support in cancer patients, Asian Pacific journal of cancer prevention : APJCP, 13 (2012) 3569-3572.
[52] J.E. Hupcey, Clarifying the social support theory-research linkage, Journal of advanced nursing, 27 (1998) 1231-1241.
[53] A.E. Brabers, J.D. de Jong, P.P. Groenewegen, L. van Dijk, Social support plays a role in the attitude that people have towards taking an active role in medical decision-making, BMC health services research, 16 (2016) 502.
[54] J.J. Arch, E.E. Bright, L.B. Finkelstein, R.M. Fink, J.L. Mitchell, D.J. Andorsky, J.S. Kutner, Anxiety and Depression in Metastatic Cancer: A Critical Review of Negative Impacts on Advance Care Planning and End-of-Life Decision Making With Practical Recommendations, JCO oncology practice, 19 (2023) 1097-1108.
[55] L. Feng, L. Zhang, H. Zhong, Perceived Parenting Styles and Mental Health: The Multiple Mediation Effect of Perfectionism and Altruistic Behavior, Psychology research and behavior management, 14 (2021) 1157-1170.
[56] K.M. Brethel-Haurwitz, M. Stoianova, A.A. Marsh, Empathic emotion regulation in prosocial behaviour and altruism, Cognition & emotion, 34 (2020) 1532-1548.
[57] A. Barasch, E.E. Levine, J.Z. Berman, D.A. Small, Selfish or selfless? On the signal value of emotion in altruistic behavior, Journal of personality and social psychology, 107 (2014) 393-413.

Click here to access/download
Supporting Information
Supporting Information.xlsx

Click here to access/download
Supporting Information
supporting file.pdf
