# Supplementary material for: Factors influencing the participation of surrogate decision-makers for advanced cancer patients in advance care planning: A cross-sectional study
Source: PLoS One. 2025 Jun 16;20(6):e0325551. doi: 10.1371/journal.pone.0325551 (PMC12169540; doi:10.1371/journal.pone.0325551)
Supplement: Attachment — Submitted filename: Reviewers in-text comment.pdf [file pone.0325551.s006.pdf]

Factors influencing the participation of surrogate decision-makers for advanced cancer patients in advance care planning: A cross-sectional study

Zhihao Han[#1], Jingjing Su[#2], Guiyue Ma[2], Yong Fang[3], Linxia Tang[2], Xiaoqin Ma[*2]

[1] Zhejiang Chinese Medical University First Clinical School of Medicine, Hangzhou City, Zhejiang Province, China

[2] Department of Nursing, Zhejiang Chinese Medical University, Hangzhou City, Zhejiang Province, China

[3] Department of Nursing, Changsha Medical College, Changsha City, Hunan Province, China

[#] These authors contributed equally to this work

[*] Correspondence: 20041028@zcmu.edu.cn

Abstract

Aim: The aim of this study was to explore the factors influencing of surrogate decision-makers (SDMs) engagement in Advance Care Planning (ACP).

Design: A cross-sectional Study.

Method: A total of 285 SDMs of advanced cancer patients were recruited using convenience sampling methods. All eligible participants completed the structured questionnaires. Univariate analysis and correlation analyses were used to explore the relationship between different variables with the ACP engagement of SDMs. Factors significant previously in univariate or correlation analyses were included in the multiple linear regression analyses with $P < 0.05$.

Results: Eight factors in univariate analyses and three factors in correlation analyses with $P<0.05$ were taken into multiple linear regression analysis. Finally, seven variables revealed a statistically significant influence on ACP engagement of SDMs and were included in the multiple linear regression model.

Conclusion: Our study suggested that some interventions should be implemented to strengthen the willingness of the surrogate decision-makers to participate in ACP. The

dissemination of ACP concepts should be strengthen, and related legislation should be established to provide support for ACP engagement in mainland China.

Impact: Based on this study, we have identified some factors influencing the ACP engagement of SMDs, which might provide basic information for construction of the educational scheme of ACP in mainland China and help to improve SDMs engagement in clinical ACP practice in future.

Patient or public contribution: SDMs of advanced cancer patients were participated in this study, and assisted to assess the validation of the measurements.

Keywords: Advance care planning, Advance directives, Surrogate decision-makers, Decision-making, Advanced cancer patients

1 Introduction

Advance Care Planning (ACP) is increasingly implemented in oncology and other domains to enable individuals to make plans about their future health care and improve consistency of care with patients' goals. In 2017 the European Association for Palliative Care proposed a definition for ACP as a process that "enables individuals to define goals and preferences for future medical treatment and care, to discuss these goals and preferences with family and health-care providers, and to record and review these preferences if appropriate" [1]. The definition clearly states that ACP is much more than the completion of advance directives (ADs), which is an instructional directive or "living will" that allows a person to make decisions or state goals of care for future medical treatment and/or appoint a close affiliate to make decisions on their behalf [2]. ACP is the discussions surrounding the patients' wishes and care and the AD is the formal document. The implementation of AD has been guaranteed in many countries and regions, such as United Kingdom, United States, Austria, Germany, etc. While, the practices of ADs were marked heterogeneity between countries of in a study of 2021, which showed that only 15 of the 28 European Union countries had developed specific rules on ADs, which are, if written, legally binding in 86% of cases [3]. AD and ACP in mainland China have not been widely adopted due to the influence of traditional Chinese culture, and there is a lack of laws and regulations to guarantee the application of ADs and ACP.

2 Background

According to the latest data from the International Agency for Research on Cancer in 2022, there were close to 20 million new cases of cancer alongside 9.7 million deaths from cancer. China contributed with 24.1% of new cases and 26.8% of cancer-related deaths [4]. Many patients in China are diagnosed with advanced stages of cancer due to delayed cancer screening and prevention efforts, and they have to face death and life-threatening complications during treatment [5]. Disease progression might result in them suddenly falling into unconsciousness or becoming incapable of expressing their own treatment preferences. In Chinese family, the family members always insist to save or sustain the patients' lives. While, life-sustaining treatments may also expose patients to grave suffering and reduce the quality of life [6]. To maintain patients' autonomy and protect their right of self-determination, discussions about medical preferences must be conducted when patients are still capable of decision-making.

ACP has emerged as a solution to this problem, enabling patients to choose their preferences for medical treatment and care even when they are incapacitated [7]. ACP also encourage patients and their families to participate in the shared decision-making discussion with healthcare professionals. This collaborative pattern helps surrogate decision makers (SDMs) understand the patient's health status, values and goals, easing decision-making burdens, and preventing conflict.

When patients experience cognitive impairment, delirium and other conditions of loss of decision-making capacity, family members are often deemed to be the critical decisions makers on their behalf. Additionally, in traditional Chinese cultural contexts, familial collective needs often supersede individual preferences. Therefore, most patients in China would choose family members, such as spouses, children or parents, as surrogate decision-makers [8] due to the family concept. Engagement of family members in the ACP process is critical component in the improvement of ACP practice.

Currently, most studies are mainly focused on patients' engagement in ACP, such as the compliance rate of ACP, or the engagement in the comprehensive ACP process measured by the ACP Engagement Survey [9-12]. However, there are few studies focused on the ACP engagement of SDMs. The purpose of this study was to explore the influencing factors of the SDMs engagement in ACP.

3 The Study

3.1 Aim

The aim of this study was to explore the factors influencing of ACP engagement of SDMs. This study might provide some information for the construction of the educational scheme of ACP in mainland China and help to improve SDMs engagement in clinical ACP practice in future.

4 Methods

4.1 Study design

This study was conducted from February 2023 to July 2023 at First Affiliated Hospital of Zhejiang Chinese Medical University. This hospital is one of the largest tertiary hospitals of Zhejiang Province, China, in which oncology department is rated as a key discipline by National Administration of Traditional Chinese Medicine of the People's Republic of China.

Participants were recruited using convenience sampling methods. Participants were invited to take part in this survey under the guidance of well-trained investigators. The investigators explained in detail the purpose of this study and obtained informed consent from all patients. All eligible participants completed the structured questionnaires by themselves or with the assist of professional investigators.

Data collection and evaluation was conducted from February 2023 to January 2024. The questionnaires were verified after participants finished to decrease missing data or illegible. Besides, double entry data recording was used to avoid the data error.

4.2 Participants and selection criteria

The inclusion criteria for SDMs of advanced cancer patients were as follows: (1) 18 years old or above; (2) blood, marital, parental relationship with the advanced tumor patient (such as, parents, spouse, children, sibling etc.) and appointment as a SDM by the patient according to the relevant regulations; (3) provision of informed consent and cooperation with the survey; (4) able to communicate and compliance to complete the questionnaire.

Based on the principles of sample size estimation in empirical methods [13], this study includes a total of 23 variables. Taking 5-10 times the number of observation variables, the sample size is estimated to be 115-230 subjects. Meanwhile, considering a dropout rate of 20%, the final sample size range determined for this study is 138-276 subjects.

4.3 Measurements

To identify the factors influencing the ACP engagement of SDMs of patients with advanced cancer, a structured questionnaire was designed in this study. Based on the results of previous studies, factors were always divided into three dimensions, the individual, environmental, and behavioral dimensions. This theory of triadic reciprocal determinism proposed a dynamic relationship model among the three dimensions [14]. This study explored how individual and environmental factors influencing the ACP engagement of SDMs by the theory of triadic reciprocal determinism. Individual factors such as self-efficacy and outcome expectations mainly manifested as life orientation ability and uncertainty about illness. Environmental factors primarily manifested as the level of social support for SDMs, and behaviors involving the evaluation of SDMs' ACP engagement.

4.3.1 Socio-demographic and cancer characteristics

The Socio-demographic and cancer characteristics questionnaire was designed by the researchers. We chose 12 variables in the questionnaire, which might affect the SDMs' engagement of ACP in previous studies, including age, gender, education level, marital status, numbers of family members, annual income, type of surrogate relationship, classification of patients' cancer, treatment expenditure, patients' independence level, experiences of making medical decision and knowledge of ACP.

4.3.2 Chinese Version of 17-item Advance Care Planning Engagement Survey for Surrogate Decision Makers (ACP-17-SDM)

The Chinese Version of ACP-17-SDM was translated by Wang Tianhang et al [15] from the English version of ACP-17-SDM [16]. The ACP-17-SDM included 3 dimensions (serving as SDM, contemplation, and readiness) with17 items. Each item was rated with 5-Point Likert Scale ranging from 1 (not at all / never) to 5 (very much / always). The total score of ACP-17-SDM ranged from 17 to 85 points with higher

scores indicating higher ACP engagement of SDMs. This Chinese Version of ACP-17-SDM was reliable to use with a total Cronbach's α coefficient of 0.915 in this study.

4.3.3 Chinese Version of Mishel Uncertainty in illness Scale-Family Member Form (MUIS-FM)

The levels of disease uncertainty in the family members of patients were evaluated with Chinese version of MUIS-FM which was translated and revised by Wenying Wang [17]. The C-MUIS-FM consisted of 4 dimensions (ambiguity, lack of clarity, lack of information, and unpredictability) covering 25 items. Each item was rated with 5-Point Likert Scale ranging from 1 (strongly disagree) to 5 (strongly agree) except item 6, 9, 17, 21, 23, 24, and 25, which were reverse rated. The total scores of C-MUIS-FM were ranging from 25 to 125 points with higher scores indicating higher level of disease uncertainty of family members. The total Cronbach's α coefficient of the C-MUIS-FM was 0.887 in this study.

4.3.4 Social support rating scale (SSRS)

The SSRS was revised by Shuiyuan Xiao [18], which was used to assess current level of social support. The scale consisted of 3 dimensions, including subjective social support, objective social support, and social support use. Items 1-4 and 8-10 were single-choice questions, which was rated with 4-Point Likert Scale from 1 point to 4 points. Item 5 had 5 sub-items (A, B, C, D, F) and each sub-item was rated from 1 point (none) to 4 points (full support).For items 6 and 7, if "no source" were selected, it would be rated with 0 point; otherwise, each source selected under "from the following sources" would add 1 point to the total scores. The total scores of SSRS ranged from 12 to 66 points, with higher scores indicating higher levels of social support. The total Cronbach's alpha coefficient of SSRS was 0.788 in this study.

4.3.5 Chinese version of Life orientation test-Revised (LOT-R)

This Chinese version of LOT-R was translated by Wengen Deng [19] from English version of LOT-R [20].The LOT-R was mainly used to assess the disposition of optimism or pessimism. The LOT-R was a single-dimensional self-assessment scale consisting of 10 items, with 3 items measuring optimism, 3 items measuring pessimism, and 4 items serving as fillers. Each item was rated with 5-Point Likert Scale ranging from 0 (strongly disagree) to 4 (strongly agree), and items 2, 4, and 5 were reverse

scored. The total scores of the LOT-R ranged from 0 to 24 points, the higher scores, and the higher level of optimism. In this study, the total Cronbach's α coefficient was 0.831.

4.4 Statistical Analysis

The Statistical Package for the Social Sciences (SPSS) software version 26.0 was used for data input and statistical analyses. The socio-demographic and cancer characteristics of SDMs were described by frequency and percentage. The scores of questionnaires were described by mean ± standard deviation or median (quartile) for normal or non-normal distribution data. Univariate analyses were used to explore the difference of ACP engagement of SDMs in different categories of socio-demographic characteristics. T test or ANOVA test and Mann-Whitney U rank-sum test or Kruskal-Wallis H rank-sum test were used for normal or non-normal distribution data, respectively. Pearson or Spearman correlation analyses were used to explore the correlations of other variables, including the uncertainty of disease, social support and optimism of participants, with ACP engagement of SDMs. Factors significant previously in univariate or correlation analyses were included in the multiple linear regression analyses with $P > 0.05$ as the removal standard.

4.5 Ethical considerations

This study was approved by Zhejiang Chinese Medical University Ethics Committee on 16th February 2023 (20230216-5). All participants were voluntary to anonymously participate in this study. Full explanation of this study was provided to all participants, and written informed consent was obtained before the interviews.

5 Results

During this study, of the 285 questionnaires administered, 9 responses were excluded because of missing data or illegible. Thus, 276 responses were finally analyzed (effective response rate 96.0%).

5.1 Socio-demographic characteristics of the participants

The participants' socio-demographic and cancer characteristics were presented in Table 1. The participants were evenly distributed by age and gender. Most of the participants

were married and lived with their family members. While, more than 2/3 (70.3% and 68.5%) of the participants had no experiences of medical decision-making or lacked knowledge of ACP. Most of the SDMs were the children (44.9%) or spouses (43.8%) of advanced cancer patients. Nearly 2/3 (63.4%) of the patients could take care of themselves in daily life.

Table 1 Socio-demographic characteristics of the participants

**Socio-demographic characteristics**		**Frequency (n)**	**Percentage (%)**
**Age (years)**	≤40	98	35.5
	41-60	113	40.9
	≥61	65	23.6
**Gender**	Female	144	52.2
	Male	132	47.8
**Education status**	Primary school or less	46	16.7
	Junior high school	72	26.1
	Senior high school	57	20.7
	College	90	32.6
	Master or greater	11	4.0
**Marital status**	Unmarried or widowed or divorced	37	13.4
	Married	239	86.6
**Experiences of making medical decision**	No	194	70.3
	Yes	82	29.7
**Knowledge of ACP**	Unknown	189	68.5
	Heard of ACP	55	19.9
	Partly known	28	10.1
	Known	4	1.4
**Treatment expenditure**	≤ ¥10,000 ($1370)	110	39.9
	≤ ¥30,000 ($4110)	120	43.5
	≤ ¥50,000 ($6850)	28	10.1
	>¥50,000 ($6850)	18	6.5

**Annual income**	≤ ¥10,000 ($1370)	112	40.6
	≤ ¥30,000 ($4110)	101	36.6
	≤ ¥50,000 ($6850)	30	10.9
	>¥50,000 ($6850)	33	12.0
**Numbers of Family members**	≤3	110	39.9
	4-5	121	43.8
	≥6	45	16.3
**Type of surrogate relationship**	Children	124	44.9
	Parents	19	6.9
	Spouse	121	43.8
	Siblings	6	2.2
	Grandchildren	6	2.2
**Classification of patients' cancer**	Digestive system tumors	112	40.6
	Hematologic cancers	34	12.3
	Genitourinary cancer	28	10.1
	Head and neck cancer	25	9.1
	Thoracic cancer	77	27.9
**Patients' independence level**	Dependent	24	8.7
	Partly dependent in daily life	77	27.9
	Independent in Daily life	62	22.5
	Completely dependent	113	40.9

5.2 Factors influencing the ACP engagement of SDMs

Base on univariate analyses, the factors significant influencing the ACP engagement of SDMs were age, education level, experiences of making medical decision, knowledge of ACP, treatment expenditure, annual income, type of surrogate relationship. While, there was no significant difference in ACP engagement of SDMs stratified by marital status, numbers of family members, classification of patients' cancer and patient's independence level (Table 2). In addition, the correlation between the uncertainty of disease, social support, optimism of participants with ACP engagement of SDMs were shown in Table 3. The ACP engagement of SDMs were negatively associated with

uncertainty of disease (P<0.01), and positively associated with social support and optimism of participants (both P<0.01).

Table 2 The difference of ACP engagement of SDMs with different socio-demographic and cancer characteristics

Characteristics	Serving as SDM	Contemplation	Readiness	Total
**Age (years)**				
≤40	25.10±3.63	11.00 (7.00)	19.00 (7.00)	55.50 (18.00)
41-60	23.38±5.25	10.00 (7.00)	17.00 (7.00)	49.00 (21.00)
≥61	21.46±5.76	7.00 (8.00)	14.00 (7.00)	43.00 (24.00)
F/H	10.996	5.605	16.536	16.604
P	＜0.001	0.061	＜0.001	＜0.001
**Gender**				
Female	22.51±4.93	9.00 (7.00)	16.00 (8.00)	47.50 (19.00)
Male	24.67±4.96	10.00 (8.00)	17.50 (9.00)	54.00 (22.00)
t/Z	-3.627	-0.557	-2.478	-2.678
P	＜0.001	0.577	0.013	0.007
**Education level**				
Primary school or less	18.85±4.77	7.00 (2.00)	13.00 (3.00)	40.74±9.02
Junior high school	21.38±4.64	7.00 (4.00)	15.00 (5.00)	45.33±10.73
Senior high school	24.93±3.63	12.00 (6.00)	19.05±4.38	55.44±10.61
College	27.00 (5.00)	13.00 (7.00)	20.00 (8.00)	57.83±10.55
Master or higher	29.00 (2.00)	16.00 (5.00)	22.36±3.53	65.27±8.75
F/H	92.646	59.416	77.940	34.457
P	＜0.001	＜0.001	＜0.001	＜0.001
**Marital status**				
Unmarried or widowed or divorced	23.11±4.60	10.19±3.95	17.00 (9.00)	48.00 (22.00)
Married	24.00 (8.00)	10.00 (8.00)	17.00 (7.00)	49.00 (21.00)
Z	-0.831	-0.161	-0.284	-0.311
P	0.406	0.872	0.776	0.756

**Experiences of making medical decision**				
No	23.00 (6.00)	8.00 (6.00)	15.00 (6.00)	46.00 (15.00)
Yes	28.00 (4.00)	14.00 (5.00)	21.00±4.45	64.00 (12.00)
Z	-7.346	-6.780	-7.294	-7.852
P	＜0.001	＜0.001	＜0.001	＜0.001
**Knowledge of ACP**				
Unknown	21.90±4.78	8.00 (6.00)	15.90±4.13	46.00 (15.00)
Heard of ACP	28.00 (3.00)	14.00 (5.00)	21.25±3.94	64.00 (11.00)
Partly known	27.36±3.47	13.61±3.34	22.14±4.08	63.11±9.15
Known	26.50±4.93	10.75±4.27	17.50±5.80	54.75±14.18
F/H	70.245	59.818	36.639	82.403
P	＜0.001	＜0.001	＜0.001	＜0.001
**Treatment expenditure**				
≤ ¥10,000 ($1370)	22.40±4.52	7.00 (6.00)	15.00 (5.00)	43.00 (14.00)
≤ ¥30,000 ($4110)	25.00 (7.00)	11.50 (7.00)	18.50 (8.00)	53.72±12.98
≤ ¥50,000 ($6850)	25.50 (7.00)	12.00 (7.00)	19.18±4.91	54.36±11.71
>¥50,000 ($6850)	28.50 (5.00)	12.17±3.20	20.56±4.25	63.00 (12.00)
H	20.812	28.671	27.781	28.729
P	＜0.001	＜0.001	＜0.001	＜0.001
**Annual income**				
≤ ¥10,000 ($1370)	21.37±4.94	7.50（6.00）	14.50 (5.00)	43.50 (16.00)
≤ ¥30,000 ($4110)	24.00 (6.00)	11.00 (7.00)	18.00 (8.00)	52.00 (21.00)
≤ ¥50,000 ($6850)	27.50 (5.00)	12.50 (6.00)	18.40±4.60	55.70±12.45
>¥50,000 ($6850)	27.00 (3.00)	12.09±3.31	20.52±3.95	58.97±8.96
H	42.865	23.014	37.549	40.237
P	＜0.001	＜0.001	＜0.001	＜0.001
**Numbers of Family members**				
≤3	24.50 (8.00)	11.50 (8.00)	18.00 (9.00)	53.50 (24.00)
4-5	23.00 (7.00)	9.00 (7.00)	16.00 (7.00)	47.00 (19.00)
≥6	24.16±3.93	8.00 (8.00)	16.00 (7.00)	48.00 (20.00)

H	4.995	5.366	4.744	5.756
P	0.082	0.068	0.093	0.056
**Type of surrogate relationship**				
Children	24.69±4.15	11.00 (7.00)	18.00 (7.00)	54.00 (19.00)
Parents	21.37±4.11	7.74±2.88	13.00 (4.00)	44.21±9.38
Spouse	22.55±5.77	9.00 (8.00)	15.00 (8.00)	47.00 (24.00)
Siblings	26.17±2.93	10.50±4.46	18.83±4.79	55.50±11.36
Grandchildren	23.83±5.19	16.00 (8.00)	19.33±4.80	56.50±12.36
F/H	4.253	11.811	16.576	15.189
P	0.002	0.019	0.002	0.004
**Classification of patients' cancer**				
Digestive system tumors	23.00 (8.00)	10.00 (8.00)	17.00 (8.00)	51.38±12.55
Hematologic cancers	24.53±3.59	9.49±3.78	16.00 (7.00)	49.00 (19.00)
Genitourinary cancer	23.64±4.65	10.50 (9.00)	17.00 (11.00)	52.00 (27.00)
Head and neck cancer	23.64±5.83	8.00 (9.00)	16.36±4.89	46.00 (27.00)
Thoracic cancer	24.00 (9.00)	10.00 (7.00)	17.00 (9.00)	51.00 (24.00)
H	1.371	2.139	2.301	0.697
P	0.849	0.710	0.681	0.952
**Patients' independence level**				
Dependent	25.50 (14.00)	12.50 (10.00)	17.46±5.70	51.25±16.50
Partly dependent in daily life	26.00 (9.00)	12.00 (8.00)	20.00 (10.00)	60.00 (23.00)
Independent in Daily life	23.42±4.74	10.00 (5.00)	16.00 (8.00)	48.00 (19.00)
Completely dependent	23.00 (6.00)	8.00 (7.00)	16.00 (6.00)	47.00 (15.00)
H	4.265	8.211	9.602	7.006
P	0.234	0.042	0.022	0.072

F: F value of ANOVA test, H: H value of Kruskal-Wallis H rank-sum test; t: t value of t test; Z: Z value of Mann-Whitney U rank-sum test.

Table 3 The correlation between the uncertainty of disease, social support, optimism of participants with ACP engagement of SDMs

**Characteristics**	Serving as SDM	Contemplation	Readiness	**Attitudes**
Uncertainty of disease	-0.485**	-0.452**	-0.469**	-0.524**
Social support	0.514**	0.410**	0.501**	0.534**
Optimism	0.356**	0.387**	0.401**	0.424**

**: P<0.01

5.3 Factors influencing ACP engagement of SDMs

Eight factors in univariate analyses and three factors in correlation analyses with $P<0.05$ were taken into multiple linear regression analysis. The stepwise regression method was performed to access the relationship between different variables. All independent variables were shown in Table 4, and the tolerance (TOL) of each variable in the model was greater than 0.1, and the variance Inflation factor (VIF) was less than 5. Thus, it was considered that there was no obvious multicollinearity between each variable.

Table 4 Independent variables assignment in multiple linear regression analysis

**Independent variable**	**Assignment**
Age	1=≤40, 2=40-60, 3=≥61
Gender	1=Female, 2=Male
Education level	1= Primary school or less, 2= Junior high school, 3= Senior high school, 4= College, 5= Master degree or higher
Experiences of medical decision-making	1=No, 2=Yes
Knowledge of ACP	1= Unknown, 2= Heard of ACP, 3= Partly known, 4= Known
Treatment expenditure	1=⩽ ¥10,000 ($1370), 2=⩽ ¥30,000 ($4110), 3=≤ ¥50,000 ($6850), 4=>¥50,000 ($6850)

Annual income	1=≤ ¥10,000 ($1370), 2=≤ ¥30,000 ($4110), 3=≤ ¥50,000 ($6850), 4=>¥50,000 ($6850)
Type of surrogate relationship	1= Children, 2= Parents, 3= Spouse, 4= Siblings, 5= Grandchildren
The uncertainty of disease	null
The social support	null
The optimism	null

Finally, as shown in Table 5, only seven variables revealed statistically significant influences on ACP engagement of SDMs and those variables included in the multiple linear regression model could explain 56.7% of the total variation (Adjusted R^2=0.567; F=52.448; P＜0.001). Those variables, except uncertainty of disease, presented a strong positive association with ACP engagement of SDMs.

Table 5 Multiple linear regression analysis of independent variables in ACP engagement of SDMs

**Variables**	**Regression coefficient**	**Standardized coefficient**	**t**	**P**
Constant	29.912	-	7.040	＜0.001
Experiences of medical decision-making	6.144	0.224	4.934	＜0.001
Education level	2.460	0.230	4.650	＜0.001
Knowledge of ACP	1.989	0.116	2.387	0.018
Treatment expenditure	1.541	0.105	2.554	0.011
Uncertainty of disease	-0.196	-0.232	-5.051	＜0.001
Social support	0.249	0.159	3.186	0.002
Optimism	0.274	0.105	2.279	0.023

6 Discussion

This study investigated the ACP engagement of SDMs of Chinese advanced cancer patients, which provided valuable insights in mainland China. To our knowledge, this study was the first large sample study focusing on the factors influencing ACP engagement of SDMs of different advanced cancer patients in mainland China. Currently, there were few studies related to ACP engagement of patients at the end life in mainland China due to the Chinese traditional culture which limited the spread and implement of ACP [15].

A total of 276 SDMs of advanced cancer patients were analyzed in this study, with balanced distribution of gender (52.2% female and 47.8% male). Most of participants were the children or spouse of the advanced cancer patients. Among the participants, SDMs younger than 60 years old were account for a large proportion of total participants (76.4%), which were similar to the results of Wang's study [15]. Combined with the surrogate relationship with patients, we considered the following possibilities of this phenomenon. On one hand, most of advanced cancer patient were the elderly, and their children became the breadwinner of family which means their children would also take the role of SDMs. On the other hand, for the some middle-aged cancer patients, their spouse might become the first choice of surrogate decision maker because their children were too young and their parents were too old to make decision. The univariate analyses showed that older SDMs had higher ACP engagement. This is similar to findings by Liu [21]. With age, the patients were more at risk of various diseases and they would think more about medical care and end-of-life issues.

Experiences of medical decision-making was significant correlated with the engagement of ACP, and participants with experiences showed more positive engagement of ACP. However, only 29.7% of the participants had experiences of medical decision-making in this study, which might attribute to the limited implementation of the doctor-patient-surrogate decision-making model in mainland China. And it would hinder SDMs from fully obtaining the patient's medical information [22-24]. A recent study presented that SDMs with experiences of decision-making would be more deliberate when making medical decisions due to the knowledge of disease trajectory [25].

Relevant research showed that education level has an impact on the cognition, the higher education level, the better individual's cognitive ability [26]. Our study showed

that 36.6% of the participants' education level were greater than college, and 31.4% of them have partial knowledge of ACP, which showed a potential correlation between education level and cognition. Besides, as the study of Keam et al [27] showed that participants with higher education level have a more positive ACP engagement than those with lower education level. Participants with high education level tended to have a strong sense of autonomy, and consider the value of life and future medical intention when actively seeking disease information [28, 29]. Conversely, participants with low education level might lack knowledge to support their thinking, although they want to express their wishes [30]. Participants had knowledge of ACP presented a more positive attitude than others, which is consistent with the study of Park et al [31]. A full understanding of new things is the prerequisite for participation. Therefore, it is important for government, media and hospitals to establish theoretical frameworks to disseminate accurate information about ACP and strengthen the public's knowledge of ACP [32]. The different types of cancer were

This study showed that the treatment expenditure played an important role in making medical decisions by SDMs for advanced cancer patients, and the high cost of treatment expenditure often puts SDMs under great economic pressure [33, 34]. The composition treatment expenditure was similar to annual income, which indicated the treatment expenditure of advanced cancer would occupy the most of annual income, bringing huge economic burden to their families [35-37]. It is recommended that medical staff should comprehensively consider the treatment needs and economic status of patients and their family, assisting them to weigh the pros and cons of different treatment options and provide personalized medical decision-making recommendations [38].

The uncertainty of disease was negatively correlated with ACP engagement. According to the uncertainty in illness theory, uncertainty arises when individuals are unable to establish an adaptive cognitive framework for related events [39]. Doctors and healthcare professionals play a vital role in facilitating communication between the patients and SDMs by offering support and guidance throughout these stages, especially for SMDs who have no medical knowledge. Social support, referring to the material and spiritual support that individuals obtain from family or other social networks [40, 41], was positively associated with engagement of ACP. However, the results was inconsistent to a study investigating the factor influencing ACP engagement of SDMs of patients with hematologic malignancies [15], which might attributing the difference

due to the characteristics of participants. Participants with high scores of social support showed more active involvement of ACP and high-quality of ACP completion [42]. Many studies have shown that individuals under good emotional state will result in more altruistic behaviors [43-45]. Optimistic SDMs would present positive attitudes to patients and actively seek help from others to find solutions when encountering difficulties. Therefore, the optimistic SDMs are more willing to participate in the discussion of patients' treatment decisions and have a more positive attitude towards ACP.

Several limitations of this study should be acknowledged. Firstly, recruiting a convenience sample from a single cancer center may limit generalizability to national target populations. Secondly, the individual perspectives of participants were not included in this study, such as the outlooks of world, life and value, which limited the and comprehensiveness. Further validation studies with a large national-wide sample and more comprehensive factors are need to warrant the conclusions.

7 Conclusion

In mainland China, some interventions should be implemented to strengthen the willingness of the SDMs to participate in ACP. One of most important measure is to strengthen the dissemination of ACP concepts and avoid the public's misunderstanding. In addition, legal support is also essential for the progress of ACP, and clearly defined legislation can provide strong support for all participants involved the decision-making processes, making ACP practice more authoritative and actionable. Based on this study, we have identified some factors influencing the ACP engagement of SMDs, which might provide basic information for the construction of the educational scheme of ACP in mainland China and help to improve SDMs engagement in clinical ACP practice in future.

Author Contributions

Conceptualization: Xiaoqin Ma.

Data curation: Zhihao Han.

Formal analysis: Zhihao Han.

Supervision: Xiaoqin Ma.

Investigation: Guiyue Ma, Linxia Tang, Yong Fang.

Validation: Jingjing Su.

Writing– original draft: Zhihao Han, Guiyue Ma.

Writing– review & editing: Jingjing Su, Xiaoqin Ma.

Conflict Statement

No conflict of interest has been declared by the authors.

Funding Information

This project did not receive any grant from funding agencies in the public, commercial, or not-for-profit sectors.

Acknowledgments

This research was not funded by any public, commercial, or nonprofit agency. No competing financial interests exist. The authors thank all members of the cancer center for their help in this study.

Data Availability Statement

Data available on request from the authors.

REFERENCES

1. Rietjens JAC, Sudore RL, Connolly M, van Delden JJ, Drickamer MA, Droger M, et al. Definition and recommendations for advance care planning: an international consensus supported by the European Association for Palliative Care. The Lancet Oncology. 2017;18(9):e543-e551. https://doi.org/10.1016/s1470-2045(17)30582-x. PMID: 28884703.
2. Ribeiro C, Pamplona P, Simonds AK. Advance care planning in patients with respiratory failure. European respiratory review : an official journal of the European Respiratory Society. 2024;33(174). https://doi.org/10.1183/16000617.0120-2024. PMID: 39537243.
3. Porcar Rodado E, Peral Sanchez D, Gisbert Grifo M. Advance Directives. Comparison of current legislation within the European Union. Spanish Journal of Legal Medicine. 2021;47(2):66-73. https://doi.org/https://doi.org/10.1016/j.remle.2020.05.011.
4. Bray F, Laversanne M, Sung H, Ferlay J, Siegel RL, Soerjomataram I, et al. Global cancer statistics 2022: GLOBOCAN estimates of incidence and mortality worldwide for 36 cancers in 185 countries. CA: a cancer journal for clinicians. 2024;74(3):229-263. https://doi.org/10.3322/caac.21834. PMID: 38572751.

5. Canny A, Mason B, Boyd K. Public perceptions of advance care planning (ACP) from an international perspective: a scoping review. BMC palliative care. 2023;22(1):107. https://doi.org/10.1186/s12904-023-01230-4. PMID: 37507777.
6. Lu Y, Gu Y, Yu W. Hospice and Palliative Care in China: Development and Challenges. Asia-Pacific journal of oncology nursing. 2018;5(1):26-32. https://doi.org/10.4103/apjon.apjon_72_17. PMID: 29379830.
7. Barry HC. Advance Care Planning Increases Execution of Advance Directives and Surrogate Decision-Maker Assignment. American family physician. 2018;98(11):682-683. PMID: 30485037.
8. Ni P, Zhou J, Wang ZX, Nie R, Phillips J, Mao J. Advance directive and end-of-life care preferences among nursing home residents in Wuhan, China: a cross-sectional study. Journal of the American Medical Directors Association. 2014;15(10):751-756. https://doi.org/10.1016/j.jamda.2014.06.006. PMID: 25066002.
9. Bravo G, Dubois MF, Wagneur B. Assessing the effectiveness of interventions to promote advance directives among older adults: a systematic review and multi-level analysis. Social science & medicine (1982). 2008;67(7):1122-1132. https://doi.org/10.1016/j.socscimed.2008.06.006. PMID: 18644667.
10. Tamayo-Velázquez MI, Simón-Lorda P, Villegas-Portero R, Higueras-Callejón C, García-Gutiérrez JF, Martínez-Pecino F, et al. Interventions to promote the use of advance directives: an overview of systematic reviews. Patient education and counseling. 2010;80(1):10-20. https://doi.org/10.1016/j.pec.2009.09.027. PMID: 19879090.
11. Sudore RL, Heyland DK, Barnes DE, Howard M, Fassbender K, Robinson CA, et al. Measuring Advance Care Planning: Optimizing the Advance Care Planning Engagement Survey. Journal of pain and symptom management. 2017;53(4):669-681.e668. https://doi.org/10.1016/j.jpainsymman.2016.10.367. PMID: 28042072.
12. Sudore RL, Stewart AL, Knight SJ, McMahan RD, Feuz M, Miao Y, et al. Development and validation of a questionnaire to detect behavior change in multiple advance care planning behaviors. PLOS One. 2013;8(9):e72465. https://doi.org/10.1371/journal.pone.0072465. PMID: 24039772.
13. Ni P, Chen JL, Liu N. Sample size estimation in quantitative research in nursing, Chinese Journal of Nursing, 45(2010) 378-380.
14. Bandura A. Social cognitive theory: an agentic perspective. Annu Rev Psychol. 2001;52:1-26. https://doi.org/10.1146/annurev.psych.52.1.1. PMID: 11148297.
15. Wang TH, Wang W, Shen WT, Shi BX, Participation in advance care planning and associated factors among surrogate decision makers of patients with hematologic walignancies, Chinese general practice, 25 (2022) 859-866.
16. Van Scoy LJ, Day AG, Howard M, Sudore R, Heyland DK. Adaptation and Preliminary Validation of the Advance Care Planning Engagement Survey for Surrogate Decision Makers. Journal of pain and symptom management. 2019;57(5):980-988.e989. https://doi.org/10.1016/j.jpainsymman.2019.01.008. PMID: 30684633.
17. Wang WY, Wang S, Xia DS, Cui GP, Wang CM, Study on reliability and validity of Chinese version of Mishel uncertainty in illness scalefamily member form, Chinese nursing research, 26 (2012) 662-663.
18. Xiao SY, Theory and research applications of the Social Support Rating Scale, Journal of clinical psychiatry, (1994) 98-100.

19. Deng WG, Wang P, Liu SS, Examining the issues and solutions for testing the dimensions of LOT and LOT-R using traditional methods, Journal of Shandong Normal University (Natural Science Edition), 24 (2009) 51-54. https://doi.org/10.3969/j.issn.1001-4748.2009.04.015.
20. Scheier MF, Carver CS, Bridges MW. Distinguishing optimism from neuroticism (and trait anxiety, self-mastery, and self-esteem): a reevaluation of the Life Orientation Test. Journal of personality and social psychology. 1994;67(6):1063-1078. https://doi.org/10.1037//0022-3514.67.6.1063. PMID: 7815302.
21. Liu X, Chen H, Zhang L, Zhang Q, Feng T, Liu D. Advance Care Planning Engagement Among Family Members of Community-Dwelling Elderly Patients With Chronic Diseases in China: A Mixed-Methods Study. Journal of hospice and palliative nursing : JHPN : the official journal of the Hospice and Palliative Nurses Association. 2022;24(2):e26-e34. https://doi.org/10.1097/njh.0000000000000829. PMID: 35045050.
22. Chen C, Cheng G, Chen X, Yu L. Information disclosure to cancer patients in Mainland China: A meta-analysis. Psycho-oncology. 2023;32(3):342-355. https://doi.org/10.1002/pon.6085. PMID: 36582008.
23. Hahne J, Liang T, Khoshnood K, Wang X, Li X. Breaking bad news about cancer in China: Concerns and conflicts faced by doctors deciding whether to inform patients. Patient education and counseling. 2020;103(2):286-291. https://doi.org/10.1016/j.pec.2019.08.022. PMID: 31455567.
24. Xu H, Yuan M. Family roles in informed consent from the perspective of young Chinese doctors: a questionnaire study. BMC medical ethics. 2024;25(1):2. https://doi.org/10.1186/s12910-023-00999-6. PMID: 38172849.
25. Bakke BM, Feuz MA, McMahan RD, Barnes DE, Li B, Volow AM, et al. Surrogate Decision Makers Need Better Preparation for Their Role: Advice from Experienced Surrogates. Journal of palliative medicine. 2022;25(6):857-863. https://doi.org/10.1089/jpm.2021.0283. PMID: 35076297.
26. Lövdén M, Fratiglioni L, Glymour MM, Lindenberger U, Tucker-Drob EM. Education and Cognitive Functioning Across the Life Span. Psychological science in the public interest : a journal of the American Psychological Society. 2020;21(1):6-41. https://doi.org/10.1177/1529100620920576. PMID: 32772803.
27. Keam B, Yun YH, Heo DS, Park BW, Cho CH, Kim S, et al. The attitudes of Korean cancer patients, family caregivers, oncologists, and members of the general public toward advance directives. Supportive care in cancer : official journal of the Multinational Association of Supportive Care in Cancer. 2013;21(5):1437-1444. https://doi.org/10.1007/s00520-012-1689-z. PMID: 23262809.
28. Wu F, Zhuang Y, Chen X, Wen H, Tao W, Lao Y, et al. Decision-making among the substitute decision makers in intensive care units: An investigation of decision control preferences and decisional conflicts. Journal of advanced nursing. 2020;76(9):2323-2335. https://doi.org/10.1111/jan.14451. PMID: 32538477.
29. Hopp FP. Preferences for surrogate decision makers, informal communication, and advance directives among community-dwelling elders: results from a national study. The Gerontologist. 2000;40(4):449-457. https://doi.org/10.1093/geront/40.4.449. PMID: 10961034.
30. Rwabihama JP, Belmin J, Rakotoarisoa R, Hagege M, Audureau E, Benzengli H, et al. Promoting patients' rights at the end of life in a geriatric setting in France: The healthcare professionals' level of knowledge about surrogate decision-makers and

advance directives. Patient education and counseling. 2020;103(7):1390-1398. https://doi.org/10.1016/j.pec.2020.01.020. PMID: 32070651.
31. Park HY, Kim YA, Sim JA, Lee J, Ryu H, Lee JL, et al. Attitudes of the General Public, Cancer Patients, Family Caregivers, and Physicians Toward Advance Care Planning: A Nationwide Survey Before the Enforcement of the Life-Sustaining Treatment Decision-Making Act. Journal of pain and symptom management. 2019;57(4):774-782. https://doi.org/10.1016/j.jpainsymman.2018.12.332. PMID: 30593911.
32. Lin Goh SS. Singapore Takes Six Steps Forward in 'The Quality of Death Index' Rankings. Asia-Pacific journal of oncology nursing. 2018;5(1):21-25. https://doi.org/10.4103/apjon.apjon_66_17. PMID: 29379829.
33. Jeong W, Kim S, Kim H, Park EC, Jang SI. Effects of life-sustaining treatment plans on healthcare expenditure and healthcare utilization. BMC health services research. 2023;23(1):1236. https://doi.org/10.1186/s12913-023-10235-x. PMID: 37950202.
34. Longo CJ, Fitch MI, Loree JM, Carlson LE, Turner D, Cheung WY, et al. Patient and family financial burden associated with cancer treatment in Canada: a national study. Supportive care in cancer : official journal of the Multinational Association of Supportive Care in Cancer. 2021;29(6):3377-3386. https://doi.org/10.1007/s00520-020-05907-x. PMID: 33403399.
35. Xu J, Wang J, Liu R, Xing J, Su L, Yu F, et al. Mental health inpatient treatment expenditure trends in China, 2005-2012: evidence from Shandong. The journal of mental health policy and economics. 2014;17(4):173-182. PMID: 25599281.
36. Yao A, Shen X, Chai J, Cheng J, Liu R, Feng R, et al. Characteristics and implications of insurance-reimbursed inpatient care for gastric and oesophageal cancers in Anhui, China. International health. 2021;13(5):446-455. https://doi.org/10.1093/inthealth/ihaa092. PMID: 33210133.
37. Fu W, Shi J, Zhang X, Liu C, Sun C, Du Y, et al. Effects of cancer treatment on household impoverishment: a multicentre cross-sectional study in China. BMJ open. 2021;11(6):e044322. https://doi.org/10.1136/bmjopen-2020-044322. PMID: 34193481.
38. van den Heuvel L, Meinders MJ, Post B, Bloem BR, Stiggelbout AM. Personalizing decision-making for persons with Parkinson's disease: where do we stand and what to improve? Journal of neurology. 2022;269(7):3569-3578. https://doi.org/10.1007/s00415-022-10969-4. PMID: 35084559.
39. Mishel MH. Reconceptualization of the uncertainty in illness theory. Image--the journal of nursing scholarship. 1990;22(4):256-262. https://doi.org/10.1111/j.1547-5069.1990.tb00225.x. PMID: 2292449.
40. Usta YY. Importance of social support in cancer patients. Asian Pacific journal of cancer prevention : APJCP. 2012;13(8):3569-3572. https://doi.org/10.7314/apjcp.2012.13.8.3569. PMID: 23098436.
41. Hupcey JE. Clarifying the social support theory-research linkage. Journal of advanced nursing. 1998;27(6):1231-1241. https://doi.org/10.1046/j.1365-2648.1998.01231.x. PMID: 9663875.
42. Arch JJ, Bright EE, Finkelstein LB, Fink RM, Mitchell JL, Andorsky DJ, et al. Anxiety and Depression in Metastatic Cancer: A Critical Review of Negative Impacts on Advance Care Planning and End-of-Life Decision Making With Practical Recommendations. JCO oncology practice. 2023;19(12):1097-1108. https://doi.org/10.1200/op.23.00287. PMID: 37831973.

43. Feng L, Zhang L, Zhong H. Perceived Parenting Styles and Mental Health: The Multiple Mediation Effect of Perfectionism and Altruistic Behavior. Psychology research and behavior management. 2021;14:1157-1170. https://doi.org/10.2147/prbm.S318446. PMID: 34377038.
44. Brethel-Haurwitz KM, Stoianova M, Marsh AA. Empathic emotion regulation in prosocial behaviour and altruism. Cognition & emotion. 2020;34(8):1532-1548. https://doi.org/10.1080/02699931.2020.1783517. PMID: 32576078.
45. Barasch A, Levine EE, Berman JZ, Small DA. Selfish or selfless? On the signal value of emotion in altruistic behavior. Journal of personality and social psychology. 2014;107(3):393-413. https://doi.org/10.1037/a0037207. PMID: 25133723.
