# Supplementary material for: Factors influencing the participation of surrogate decision-makers for advanced cancer patients in advance care planning: A cross-sectional study
Source: PLoS One. 2025 Jun 16;20(6):e0325551. doi: 10.1371/journal.pone.0325551 (PMC12169540; doi:10.1371/journal.pone.0325551)
Supplement: Attachment — Submitted filename: PONE-D-24-35495_R2_reviewer comment.pdf [file pone.0325551.s009.pdf]

PLOS ONE

Factors influencing the participation of surrogate decision-makers for advanced cancer patients in advance care planning: A cross-sectional study

--Manuscript Draft--

Manuscript Number:	PONE-D-24-35495R2
Article Type:	Research Article
Full Title:	Factors influencing the participation of surrogate decision-makers for advanced cancer patients in advance care planning: A cross-sectional study
Short Title:	Influencing factors of participation in ACP
Corresponding Author:	Xiaoqin Ma<br>Zhejiang Chinese Medical University<br>Hangzhou, Zhejiang CHINA
Keywords:	Advance care planning, Advance directives, Surrogate decision-makers, Decision-making, Advanced cancer patients
Abstract:	Aim: The aim of this study was to explore the factors influencing of surrogate decision-makers (SDMs) engagement in Advance Care Planning (ACP).<br>Design: A cross-sectional Study.<br>Method: A total of 285 SDMs of advanced cancer patients were recruited using convenience sampling methods. All eligible participants completed the structured questionnaires. Univariate analysis and correlation analyses were used to explore the relationship between different variables with the ACP engagement of SDMs. Factors significant previously in univariate or correlation analyses were included in the multiple linear regression analyses with P <0.05.<br>Results: Eight factors in univariate analyses and three factors in correlation analyses with P<0.05 were taken into multiple linear regression analysis. Finally, seven variables revealed a statistically significant influence on ACP engagement of SDMs and were included in the multiple linear regression model.<br>Conclusion: This cross-sectional study demonstrated that multiple factors, including experiences of medical decision-making, education level, knowledge of ACP, treatment expenditure, uncertainty of disease, social support and life orientation, significantly impact the engagement of SDMs in ACP.<br>Impact: Based on this study, we have identified some factors influencing the ACP engagement of SDMs, which might provide basic information for construction of the educational scheme of ACP in mainland China and help to improve SDMs engagement in clinical ACP practice in future.<br>Patient or public contribution: SDMs of advanced cancer patients were participated in this study, and assisted to assess the validation of the measurements.
Order of Authors:	Zhihao Han
	Jingjing Su
	Guiyue Ma
	Yong Fang
	Linxia Tang
	Xiaoqin Ma
Response to Reviewers:	Response to Reviewers<br>PONE-D-24-35495R1<br>Factors influencing the participation of surrogate decision-makers for advanced cancer patients in advance care planning: A cross-sectional study<br>We are very grateful to the reviewers for their revised comments on the manuscript, which are very important for the quality of the manuscript. On behalf of all the contributing authors, I would like to express our sincere appreciations of your letter and reviewers' constructive comments concerning our article entitled "Factors influencing the participation of surrogate decision-makers for advanced cancer patients in advance care planning: A cross-sectional study". We revised the manuscript again based on the

Powered by Editorial Manager® and ProduXion Manager® from Aries Systems Corporation

reviewers' comments, hoping to meet PLOS ONE's publication criteria.
Reviewer #1: The authors do not fully explore the principles behind advance care planning and substitute decision making. Their analysis largely ignores the fundamental issues of patient autonomy and of decision makers needing to implement the patient's wishes. The authors mention that these are Western concepts that are new to China, but they do not make pay substantial attention to that point. The authors' conclusions are sound, but the design of the study did not fully explore the points made in the conclusion. The research design was not the most appropriate design for this topic.
Response: Many thanks to the reviewers for their constructive comments. There have been comprehensive studies on the autonomy of cancer patients in participating in ACP, and the focus group of our study is the surrogate decision-makers of cancer patients. We only conducted a questionnaire survey on the willingness and influencing factors of surrogate decision-makers before formally participating in ACP. The surrogate decision-makers did not actually participate in ACP. Because currently ACP has not been fully implemented in mainland China, and no relevant laws have been promulgated, but our research conclusions can provide some suggestions for law makers. In addition, ACP began to rise in countries such as Europe and the United States, and mainland China should learn from it. Currently, mainland China has very limited policies and laws regarding ACP. Therefore, we are unable to provide a substantive explanation of the implementation of ACP in mainland China.
In response to the issue mentioned by the reviewer that the research design is not suitable for this topic, we would like to explain that conducting quantitative research is the most important part of our research topic. We are currently conducting qualitative research on patients' surrogate decision-makers, with the purpose of supplementing and explaining the quantitative research results. We hope that subsequent research results can also be submitted to the PLOS ONE and have opportunity to be published.
Reviewer #2: There are major areas that need revision to strengthen the manuscript for publication. These have been highlighted in the attached file.
1) Multiple grammatical errors, and flow of ideas, need to be reviewed and edited throughout the manuscript. Please have the manuscript reviewed for grammatical and sentence structure errors to bring more clarity to the content.
Response: We sincerely appreciate the reviewers' insightful comments and constructive suggestions, which have significantly improved the quality of our manuscript. We have polished the grammar and sentence structure of the manuscript and hope that the revised manuscript will meet the publication requirements in terms of grammar and sentence structure.
2) Need to check the facts again, for example, the implementation of AD has been guaranteed in UK etc. This is mentioned in the introduction. However, it is not guaranteed or legally binding there due to multifactorial reasons including legal and practical. Kindly review for each country listed and remove "etc." wherever possible in the manuscript.
Response: The authors are grateful for the valuable feedback provided by the reviewers, as it has greatly enhanced the clarity and rigor of this work. We misspelled the England into UK. We have checked and revised the issues raised by the reviewer.
3) Background:
•Last paragraph of this section talks a lack of existing research on ACP engagement by SDMs. Are you referring to the situation in China? Please specify and elaborate with current evidence to support the need for this study.
•Talk about relational aspect to caregiving in Asian cultures, and how does it impact involvement in ACP.
•It is unclear if authors are referring to the need for the SDMs to engage in ACP with their patients living with cancer or by themselves. Please clarify. ACP is not possible without the presence of the person in question.
Response: We thank the reviewers for their thoughtful critiques, which have guided us in refining both the methodology and presentation of results. We first explain the doubts raised by the reviewers. We then discussed the impact of Asian nursing cultural background on participation in ACP. In addition, we need to explain that ACP emphasizes the joint participation of patients and patient surrogate decision-makers. Therefore, the main purpose of our study is to understand the willingness and influencing factors of patients' surrogate decision-makers to participate in the formulation and implementation of patients' ACP, rather than to explore the surrogate decision-makers themselves' participation in ACP. There are many studies focus on ACP engagement, while the study about ACP engagement of SDMs in mainland China

	is less, and this is the first large sample study focusing on the factors influencing ACP engagement of SDMs of different advanced cancer patients. We hope that our explanation will provide reviewers with a clear understanding of the purpose of this study.<br>4) Study Design:<br>•Clearly identify the study design used in this section e.g., cross sectional study.<br>Response: The constructive criticisms from the reviewers have been instrumental in strengthening the arguments and addressing gaps in the original submission. We provide a clear explanation of study design types in this section.<br>5) Participants and selection criteria:<br>•Consider using the term, "participants" instead of subjects.<br>•Move participant related information in this section from the study design section.<br>•Clearly identify who recruited participants and how e.g., nurse or doctor in the department. What was the role of investigators in recruitment. Procedure of recruitment remains unclear.<br>•Was the survey electronic or paper-based.<br>Response: We deeply acknowledge the reviewers' expertise, and their detailed suggestions have led to a more robust and coherent analysis. We have replaced "subjects" with "participants" based on comments made by reviewers. We have moved participant related information in this section from the study design section. Our investigators are hospital nurses and the questionnaire is in the form of a paper questionnaire. The investigators were nurses in the department, and completed all the recruitment.<br>6) Measurements:<br>•Please cite the studies utilized for devising sociodemographic survey.<br>Response: We extend our gratitude to the reviewers for their meticulous evaluation. We have added relevant references where necessary in the manuscript.<br>7) Results:<br>•How was "gender" captured, did you only include female and male? These are biological constructs whereas, gender is a social construct. Please review and change accordingly.<br>Response: We sincerely appreciate the reviewer's insightful comment regarding the distinction between biological sex and gender as a social construct. In response to this concern, we have replaced the term "gender" with "sex" throughout the manuscript.<br>8) Discussion:<br>•Your point about treatment expenditure and ACP is not clear. What are the clear recommendations here? Do you think people with less might be coerced into ACP discussions?<br>Response: Thank you for your valuable feedback. We understand your concern about the clarity of the relationship between treatment expenditure and advance care planning (ACP) and the potential implications of economic pressure on decision - making.<br>In our study, we found that high treatment costs significantly increased the economic burden on families, which in turn made them more inclined to consider less aggressive medical care measures. This economic pressure led to a higher willingness to participate in ACP discussions. It's important to note that this does not necessarily mean that people with less economic means were coerced into ACP discussions. Rather, the financial strain influenced their preferences and considerations regarding end - of - life care options.<br>To address your concerns and provide clearer recommendations, we suggest that healthcare providers should be aware of the economic impact on patients and their families when discussing treatment options and ACP. They should offer support and guidance to help patients and their families make informed decisions that align with their values and financial situations. Additionally, policymakers could consider measures to reduce the financial burden of cancer treatment, such as improving insurance coverage and providing financial assistance programs, to ensure that patients have equal access to appropriate care and are not unduly influenced by economic factors in ACP discussions.<br>We have revised the manuscript to better clarify these points and provide more detailed recommendations. We hope this addresses your concerns and enhances the quality of our research.<br>•Throughout your discussion, you don't talk about the central role of the patient in ACP. ACP can't happen without the active involvement of the patient. What are your recommendations to bring patients and SDM together to involve in ACP.

Powered by Editorial Manager® and ProduXion Manager® from Aries Systems Corporation

Response: Thank you for your insightful comment. You are right that the patient's central role in advance care planning (ACP) is crucial. In our study, we focused on the factors affecting the involvement of SDMs. However, we acknowledge that we did not sufficiently emphasize the patient's active involvement in ACP. To address your concern and provide clear recommendations on how to bring patients and SDMs together in ACP, we suggest the following:

Patients play a central role in the development and implementation of the entire ACP, and it is crucial to facilitate communication between patients and SDMs. Healthcare providers should play an active role in facilitating communication between patients and SDMs. This includes providing clear and comprehensive information about ACP, its benefits, and the importance of the patient's active participation. Educational sessions and materials can help both patients and SDMs understand the process and their respective roles. Establishing a trusting relationship between patients, SDMs, and healthcare providers is essential. This can be achieved through open and empathetic communication, where patients feel comfortable expressing their wishes and concerns. Each patient and family is unique, and the approach to ACP should be tailored to their specific needs and circumstances. Healthcare providers should be sensitive to cultural, religious, and personal beliefs and preferences, and adapt their communication and support accordingly.

We have revised our manuscript to better address the patient's central role in ACP and to provide more detailed recommendations on how to involve both patients and SDMs in the process. We hope these changes address your concerns and enhance the quality of our research.

•What is your opinion on goals of care discussion for patients who are not able to participate in ACP process?

Response: Thank you for raising this important issue. You are correct that for patients who are not able to participate in the ACP process, the goals of care discussion become even more critical. In our study, titled "Factors influencing the participation of surrogate decision - makers for advanced cancer patients in advance care planning: A cross - sectional study," we focused on the factors affecting the involvement of SDMs. However, we acknowledge that we did not sufficiently address the specific needs of patients who cannot participate in ACP.

We should enhance communication between patients and surrogate decision-makers and emphasize the guiding role that healthcare providers play in this. We have added relevant content in the discussion section. We hope these changes address your concerns and enhance the quality of our research.

•Future implications for practice and research need to be further elaborated.

Response: Thank you for your valuable feedback. We appreciate your suggestion that the future implications for practice and research need to be further elaborated. In response to this, we have revised our manuscript to include more detailed discussions on the future directions for both clinical practice and research.

Our study highlights the importance of SDMs in ACP for advanced cancer patients. Future practice should focus on providing healthcare providers with enhanced training on how to effectively communicate with SDMs and patients about ACP. This includes training on cultural sensitivity, communication techniques, and strategies to overcome barriers to ACP discussions. ACP should be integrated into routine care for advanced cancer patients. Healthcare providers should make it a standard practice to discuss ACP with patients and their SDMs early in the disease trajectory. This can help ensure that patients' preferences and values are respected and that their care aligns with their wishes. In addition, future research should focus on longitudinal studies to better understand the long - term impact of ACP on patient outcomes and family satisfaction. Meanwhile, there is a need for more intervention studies to test the effectiveness of different ACP strategies.

We have revised our manuscript to include these points in the discussion section, providing a more comprehensive outlook on the future implications of our study. We believe these additions will enhance the value of our research and provide a clearer direction for future practice and research.

9) Limitations:

•Please elaborate on your point about "individual perspectives of participants were not included."

Response: Thank you for your insightful comment. We apologize for not elaborating on the point about "individual perspectives of participants were not included" in the original manuscript. Here's a more detailed explanation:

In this study, we mainly focused on the objective data and collective patterns. The

individual perspectives, such as the outlooks of world, life and value, were indeed not taken into account. This is because the research design was primarily aimed at exploring the general trends and relationships among the variables at a macro level. The inclusion of individual perspectives would have required a more in-depth and qualitative approach, which was beyond the scope of the current research. However, we fully acknowledge that this limitation has affected the comprehensiveness of the study. We will make up for this shortcoming in subsequent research. We have also suggested that future research could adopt a mixed - methods approach to incorporate both the objective data and the individual perspectives, so as to provide a more comprehensive understanding of the issue.

Once again, we appreciate your valuable suggestion and we have made the corresponding revisions to improve the quality of the paper.

Reviewer #3: The first and most crucial comment is to send your manuscript to professional editing before getting published. There were many grammatical mistakes and inconsistent use of terms, such as SDMs vs SMDs, severely affect readers' understanding. More specific comments are as below.

1) Section 1: introduction

•The connection between ACP & AD was not highlighted, despite the two concepts were compared. Why promoting ACP is significant and how does it relate to the completion of AD? Please explain.

Response: Thank you for your insightful comment regarding the connection between Advance Care Planning (ACP) and Advance Directives (AD). We appreciate this opportunity to clarify and elaborate on the significance of promoting ACP and its relation to the completion of AD.

Advance Care Planning (ACP): ACP is a process that involves discussions between patients, surrogate decision-makers, and healthcare providers about the patient's values, preferences, and goals for future healthcare. It is a dynamic and ongoing process that helps ensure that the patient's wishes are respected and followed, especially when they are no longer able to make decisions for themselves.

Advance Directives (AD): AD are legal documents that outline a patient's preferences for medical care in situations where they may not be able to communicate their wishes. AD can include living wills and durable power of attorney for healthcare.

Integration of AD into ACP: AD can be an important component of the ACP process. While ACP is a broader process that includes discussions and planning, AD serves as a legal document that formalizes these preferences. By promoting ACP, we can ensure that patients' preferences are not only discussed but also documented in a legally binding manner.

We have revised our manuscript to better highlight the connection between ACP and AD. We believe these additions will enhance the clarity and value of our research.

2) Section 2: Background

•Consider using the term "individual" instead of "patient" to minimize stigmatization.

Response: Thank you for your insightful suggestion. We fully agree with your point about minimizing stigmatization. In response to your comment, we have carefully reviewed the manuscript and have made the necessary changes to replace "patient" with "individual" where appropriate. We believe this amendment will enhance the inclusivity and sensitivity of our work. Thank you again for your valuable guidance.

3) Section 3: Aim

•Suggest moving the last sentence of this paragraph to the background section. It highlights the significance of the study rather than aim of this study.

Response: Thank you for your suggestion. We have carefully considered your comment and have moved the last sentence of the paragraph to the background section as you recommended. This change has been made to ensure that the paragraph now clearly focuses on the aim of the study, while the significance of the study is appropriately highlighted in the background section. Thank you for your helpful guidance.

4) Section 4.1: Study design

•Please state that this is a cross-sectional survey.

Response: Thank you for your suggestion. We appreciate your attention to the details of our study design. You are absolutely right that this is a cross-sectional survey. We have added a statement to clarify this in the relevant section of the manuscript. We believe this addition will provide a clearer description of the study design for our readers.

•Please clarify the duration of data collection period. It is not necessary to mention the period of evaluation (i.e. from July 2023 to January 2024).

Response: Thank you for your suggestion. We have revised the manuscript to clarify the duration of the data collection period. We have removed the mention of the evaluation period (i.e., from July 2023 to January 2024) as you suggested. The revised sentence now reads: “This data collection study was conducted from February 2023 to July 2023 at First Affiliated Hospital of Zhejiang Chinese Medical University.” We believe this change will provide a clearer and more concise description of the study timeline. Thank you again for your valuable feedback.
•Please state the data collection approach. Whether participants were invited to complete a self-report questionnaire or assisted by research assistants.
Response: Thank you for your suggestion. We have revised the manuscript to clarify the data collection approach.
Participants were recruited using convenience sampling methods. Paper questionnaires were used for data collection during the research process. Participants were invited to take part in this survey under the guidance of well-trained investigators. The investigators were hospital nurses who were responsible for explaining the study to the SDMs, and responsible for recycling and checking the paper questionnaires. The investigators were available to assist participants during the questionnaire filling process if they needed it, but most participants completed the questionnaire independently.
We believe this clarification will provide a better understanding of the data collection process for our readers.
5) Section 4.2: Participant selection
•Please clarify how SDM is appointed in your study setting. The phrase “appointed according to relevant regulations” is ambiguous.
Response: Thank you for your suggestion. We understand that the phrase “appointed according to relevant regulations” is ambiguous. We have revised the manuscript to clarify how SDM is appointed in our study setting. The revised sentence now reads as follows:
blood, marital, parental relationship with the advanced tumor patient (such as, parents, spouse, children, sibling etc.) and being appointed as a SDM by the patient through a formal written designation, in accordance with the ethical principles and clinical guidelines relevant to the protection of patients’ rights and interests.
We believe this revision provides a clearer explanation of the appointment process for SDMs in our study.
6) Section 4.3: Measurements
•Please cite the references that support your choice of exploring factors under the three dimensions.
Response: Thank you for your suggestion. We have revised the manuscript to include the references that support our choice of exploring factors under the three dimensions of person, environment, and behavior based on the Triadic Reciprocal Determinism theory. Specifically, we have added the following references:
Bandura A. Social cognitive theory: an agentic perspective. Annu Rev Psychol. 2001; 52:1-26. https://doi.org/10.1146/annurev.psych.52.1.1. PMID: 11148297.
The reference provide a solid theoretical foundation for our decision to explore factors under the three dimensions of person, environment, and behavior in our study.
7) Section 4.3.3 to 4.4.5: Instruments
•The description of scoring methods was lengthy. Consider delete it as long as the total score is reported for analysis. Showing the direction of total score is adequate.
Response: Thank you for your suggestion. We have carefully considered your comment regarding the description of the scoring methods. We understand that the detailed description may have been lengthy. In response to your suggestion, we have revised the manuscript to delete the detailed scoring methods, as long as the total score is reported for analysis. We believe that showing the direction of the total score is adequate for the purpose of this study.
8) Section 4.4 Statistical analysis
•The term “cancer characteristics of SDMs” are ambiguous. The SDMs are not cancer patient. Do you mean “the characteristics of cancer of their care recipients”.
Response: Thank you for pointing out the ambiguity in the term “cancer characteristics of SDMs.” You are absolutely right that SDMs are not cancer patients themselves. We meant to refer to “the characteristics of cancer of their care recipients.” We have revised the manuscript to clarify this terminology. Thank you again for your valuable feedback.
•Please state clearly if an independent t-test or paired t-test was used.
Response: Many thanks to the reviewers for their suggestions. In the statistical

analysis part of this study, the independent T-test was used, and the paired T-test was not used. We have made this clear in the manuscript.
•When stating the p-value. Please consider if a small letter “p” or a capital letter “P” should be used.
Response: Thank you for your attention to the details of our manuscript. You raised a good point regarding the use of "p" or "P" when stating the p-value. We have reviewed the manuscript and will ensure that the p-value is consistently presented with a capital letter "P" in accordance with standard statistical reporting conventions.
•Consider addressing how missing data was treated in this section.
Response: Thank you for your suggestion. We have revised the manuscript to address how missing data was treated. During the data collection process, investigators will check the paper questionnaires filled out by participants to ensure the integrity of the data. However, during the data entry process, we still found 9 paper questionnaires with missing data and difficult to identify data. We eventually deleted these questionnaires to ensure the integrity of all data. We believe that this approach is appropriate for our study. Thank you again for your valuable feedback.
9) Section 5.2: Factors influencing the ACP engagement of SDMs
•This section reported the results of univariate analyses. The heading should be “factors associated with the ACP engagement of SDMs”
Response: Thank you for your suggestion. We have revised the heading of this section to “Factors associated with the ACP engagement of SDMs” as you recommended. We believe this change will make the content of this section clearer and more consistent with the results of the univariate analyses.
•The section heading should not be the same for section 5.2 and 5.3
Response: The thoughtful remarks from the reviewers have not only improved the manuscript but also deepened our understanding of the topic:
What is presented in 5.2 is the result of univariate analysis; what is presented in 5.3 is the result of multiple linear regression analysis, that is, the variables with statistical significance in the univariate analysis are further analyzed. In order to avoid duplication of the titles of 5.2 and 5.3, we modify the title of 5.3 to “Factors associated with the ACP engagement of SDMs (Base on multiple linear regression analysis).”
10) Table 2:
•The heading of columns were not aligned with the heading of table. If subscale score is reported, please indicate these are the three subscale component of ACP engagement.
Response: We are indebted to the reviewers for their rigorous feedback. The second, third, and fourth columns of data in Table 2 represent the scores of the three dimensions of Serving as SDM, Contemplation, and Readiness in the ACP-17-SDM scale respectively. The fifth column represents the total score of the above three dimensions in a specific group of people. We hope that our explanation can make the reviewers understand the content in Table 2.
•In the column heading, please indicate “mean (SD)” was reported in the table.
Response: Thank you for your careful consideration of our manuscript and for pointing out the need to clarify the statistical measures used in the table.
We have conducted normality tests on all the data. Since some of the data were found to be non - normally distributed, we have used “mean (SD)” to describe the normally distributed data and “median (IQR)” for the non - normally distributed data in the table. We have revised the column heading in the table to clearly indicate the statistical measures used. We believe this revision will make the data presentation more accurate and understandable.
•The choice of statistical tests for certain variables were queried. Please review the test being used for “marital status”, “experience of making medical decision”
Response: We have the following explanations for the questions raised by reviewers and editors:
We divided marital status into two groups to clarify the differences in Serving as SDM, Contemplation, Readiness, and Total between the groups. After consulting with statistical experts and reviewing relevant literature, we concluded that the Mann-Whitney U test (Z test) is a non-parametric method suitable for comparing two independent samples when the data do not follow a normal distribution. It assesses whether the two groups come from the same distribution by comparing the rank sums of the data. Therefore, it is appropriate for the analysis of the variable of marital status. Similarly, the Mann-Whitney U test (Z test) is also applicable for the analysis of medical decision-making experience.
•Please state the level of significance at the bottom of the table.

Powered by Editorial Manager® and ProduXion Manager® from Aries Systems Corporation

Response: Thank you for your suggestion. We have made the following revision to our manuscript:
We have added the level of significance at the bottom of the table. The level of significance is set at 0.05, which is a commonly used threshold for determining statistical significance. This information is now clearly stated at the bottom of the table. Thank you for your attention to this detail. We believe this revision will make the statistical results in our manuscript clearer to the readers.
11) Table 3:
•Please clarify whether the construct reported here should be named as "life orientation" or "optimism"? Please align with section 4.3.5.
Response: Thank you for your comment. We have carefully considered your suggestion and have made the following revision:
We have changed the construct from "optimism" to "life orientation" throughout the manuscript to align with section 4.3.5. At the same time, we have made changes to other descriptions related to "life orientation" in the manuscript. This change ensures consistency and accuracy in the terminology used in our study.
•What was the variable "attitudes" reported here? It was not described in section 4.3
Response: Thank you for your comment. We recognize that the description of "attitudes" here was inappropriate:
What we intended to convey is that "attitudes" represents the total score of the three dimensions of the ACP-17-SDM scale (serving as SDM, contemplation, and readiness), and we aimed to explore the correlation between the total score of the ACP-17-SDM scale and the three variables Uncertainty of disease Social support and Life orientatio. We have changed "attitudes" to "Total" to correspond to the contents of Table 2. We have revised the relevant content in the manuscript to clarify this point.
12) Section 5.3
•The section heading should not be the same as section 5.2.
Response: Both reviewers raised the same question, and we have made positive changes to this question:
What is presented in 5.2 is the result of univariate analysis; what is presented in 5.3 is the result of multiple linear regression analysis, that is, the variables with statistical significance in the univariate analysis are further analyzed. In order to avoid duplication of the titles of 5.2 and 5.3, we modify the title of 5.3 to "Factors associated with the ACP engagement of SDMs (Base on multiple linear regression analysis)." At the same time, combined with the comments of another reviewer, we revised the title of 5.2 to: Factors associated with the ACP engagement of SDMs.
•Please check the presentation format of table 5. The collinearity statistics, TOL and VIF should be included in the table. Values for the constant is not necessarily to be reported.
Response: Thank you for your comment. We have checked the presentation format of Table 5 and made the following revisions:
We have included the collinearity statistics, Tolerance (TOL) and Variance Inflation Factor (VIF), in Table 5. We have removed the values for the constant, as they are not necessary to be reported. We believe these changes will improve the clarity and completeness of the table.
•Please add the level of significance/ removal standard at the bottom of the table.
Response: Thank you for your comment. We have added the level of significance at the bottom of the table. The level of significance is set at 0.05. These values are now clearly stated at the bottom of the table.
13) Section 6: Discussion
•The conclusion "The univariate analyses showed that older SDMs had higher ACP engagement" was not supported by the statistics. From table 2, the mean ACP engagement score of the age group 18-40 is the highest among all. Please update your discussion.
Response: We sincerely appreciate the reviewer's insightful comment regarding the conclusion about the relationship between age and ACP engagement. Upon re-examining Table 2, we realized that the mean ACP engagement score of the 18-40 age group was indeed the highest among all age groups, which contradicts the previous conclusion that "older SDMs had higher ACP engagement." We have updated the discussion section to reflect this finding and provide a more accurate interpretation of the data.
In the revised discussion, we have highlighted that the 18-40 age group showed the highest level of ACP engagement, and we have explored potential reasons for this observation. We have also removed the previous conclusion.

Powered by Editorial Manager® and ProduXion Manager® from Aries Systems Corporation

	We apologize for the oversight and are grateful for the reviewer's attention to detail. We believe that the revised manuscript provides a clearer and more accurate representation of the study findings.<br>•The discussion between educational level, cognitive ability and knowledge of ACP was not logical. People having higher educational level doesn't mean they have higher cognitive ability, as they may suffer from cognitive problem such as memory problem. The discussion followed cannot address the need of the less educated group. Please revise the implication drawn from this finding.<br>Response: We sincerely appreciate the reviewer's valuable feedback regarding the discussion of the relationship between educational level, cognitive ability, and knowledge of advance care planning (ACP). Upon reflection, we recognize that the original discussion incorrectly assumed a direct correlation between higher educational level and higher cognitive ability, overlooking the possibility of cognitive issues such as memory problems. We have revised the discussion to address this oversight.<br>In the revised manuscript, we have deleted the contradictory content in the manuscript. We have also emphasized the need to consider the specific needs and challenges of individuals with lower educational levels, ensuring that our implications are more inclusive and comprehensive.<br>Thank you again for your insightful comments, which have helped us improve the manuscript significantly.<br>•The discussion made based on the result of table 3 was not logical. Please explain why people having higher treatment expenditure reported higher level of ACP engagement; and why people having higher level of uncertainty of disease reported lower level of ACP engagement. Followed by the discussion of their implications.<br>Response: We apologize for the lack of clarity in the original discussion and appreciate the reviewer's valuable feedback. We have revised the discussion to better explain the relationship between higher treatment expenditure and higher ACP engagement. Table 2 (univariate analyses) and table 5 (multiple linear regression analysis) both show that treatment expenditure is an influencing factor for SDMs to participate in patients' ACP. The results in Table 2 show that SDMs with higher treatment expenditures have higher scores on the ACP-17-SDM scale, indicating that SDMs are more willing to participate in patients' ACP, and the differences between different groups are statistically significant. Therefore, we believe that SDMs with higher treatment expenditures have greater willingness to participate in patient ACP. SDMs with higher treatment expenditure may be more likely to engage in ACP due to several factors. Firstly, they may have a greater awareness of the importance of planning for future healthcare decisions, as they have already invested significantly in their treatment. Secondly, higher treatment expenditure may also be associated with better access to healthcare information and resources, making these individuals more informed and willing to participate in ACP. They may have established a closer relationship with their healthcare providers during the treatment process, which makes them feel more comfortable and confident in initiating ACP discussions.<br>We have also revised the discussion to clarify why SDMs with higher levels of disease uncertainty reported lower levels of ACP engagement. The results of Table 3 (correlation analysis) and Table 5 (multiple linear regression analysis) both show that there is a negative correlation between SDMs' disease uncertainty scores and ACP-17-SDM scores, that is, the stronger the disease uncertainty, the weaker the SDMs' willingness to participate in patient ACP. This is because high uncertainty increases anxiety and fear, making it difficult for them to think about and plan for future care. They may be so focused on the unknown aspects of the illness, such as prognosis and treatment outcomes, that they find it challenging to engage in ACP discussions. Additionally, the lack of clear information about the disease and its progression may hinder their ability to make informed decisions and express their preferences, further reducing their willingness to participate in ACP.<br>We hope our response can answer the reviewer's questions. We would like to thank you again for your questions. This is very important for improving the quality of our manuscript.<br>•The recommendations made (public education and legal support) were not drawn from the study findings. Please tailored your recommendation specifically to a specific population that need extra attention and support to enhance their ACP engagement. And suggest what components should be included in the ACP intervention. Recommendations should be discussed in Section 6 instead of section 7.<br>Response: We sincerely appreciate the reviewer's valuable feedback. We have carefully considered the comments and made the following revisions to our manuscript:

	We have made recommendations for specific groups of people who need support and attention and have included these in Part 6. In addition, we have modified the conclusion section to summarize the study and point out the significance of the study for clinical practice. At the same time, we also provide an outlook for the next step of research.<br>We hope these revisions address the reviewer's concerns and enhance the quality of our manuscript. Thank you again for your valuable feedback.<br>•Please clarify the meaning of "validation studies".<br>Response: Thank you for your comment. We apologize for any ambiguity in our original statement. By "validation studies", we refer to research efforts that aim to confirm the reliability and validity of our findings. In this context, it means conducting further studies with a larger, national - wide sample and incorporating more comprehensive factors to ensure that our conclusions are robust and generalizable. Thank you for your attention to this matter. We have revised the sentence for clarity: "Finally, the individual perspectives of participants, such as their worldviews, life outlooks, and values, were not included in this study, which limited the comprehensiveness of our conclusions. Further studies with a large, national - wide sample and more comprehensive factors are needed to substantiate our findings."<br>We have made every effort to revise the manuscript. This revision note aims to address the questions raised by the reviewers. We would like to express our gratitude once again for the suggestions provided by the reviewers, which are crucial for improving the quality of the research.
**Additional Information:**	
**Question**	**Response**
**Financial Disclosure**<br><br>Enter a financial disclosure statement that describes the sources of funding for the work included in this submission. Review the submission guidelines for detailed requirements. View published research articles from *PLOS ONE* for specific examples.<br><br>This statement is required for submission and **will appear in the published article** if the submission is accepted. Please make sure it is accurate.<br><br>**Funded studies**<br>Enter a statement with the following details:<br>• Initials of the authors who received each award<br>• Grant numbers awarded to each author<br>• The full name of each funder<br>• URL of each funder website<br>• Did the sponsors or funders play any role in the study design, data collection and analysis, decision to publish, or preparation of the manuscript?<br><br>Did you receive funding for this work?	The author(s) received no specific funding for this work.

Powered by Editorial Manager® and ProduXion Manager® from Aries Systems Corporation

**Competing Interests**<br><br>Use the instructions below to enter a competing interest statement for this submission. On behalf of all authors, disclose any competing interests that could be perceived to bias this work—acknowledging all financial support and any other relevant financial or non-financial competing interests.<br><br>This statement is required for submission and **will appear in the published article** if the submission is accepted. Please make sure it is accurate and that any funding sources listed in your Funding Information later in the submission form are also declared in your Financial Disclosure statement.<br><br>View published research articles from *PLOS ONE* for specific examples.<br><br>**NO authors have competing interests**<br><br>Enter: *The authors have declared that no competing interests exist.*<br><br>**Authors with competing interests**<br><br>Enter competing interest details beginning with this statement:<br><br>*I have read the journal's policy and the authors of this manuscript have the following competing interests: [insert competing interests here]*<br><br>* typeset	The authors have declared that no competing interests exist.
**Ethics Statement**<br><br>Enter an ethics statement for this submission. This statement is required if the study involved:<br><br>• Human participants<br>• Human specimens or tissue<br>• Vertebrate animals or cephalopods	Ethical clearance was sought from Zhejiang Chinese Medical University ethics committee. Clearance to collect data was also sought from ethical committees in the study settings. Participation in the study was entirely voluntary; participants were provided written informed consent to participate and took part anonymously. Ethical approval number is 20230216-5, and the approval date is 16th February 2023.

• Vertebrate embryos or tissues<br>• Field research<br><br>Write "N/A" if the submission does not require an ethics statement.<br><br>General guidance is provided below. Consult the submission guidelines for detailed instructions. **Make sure that all information entered here is included in the Methods section of the manuscript.**	

Powered by Editorial Manager® and ProduXion Manager® from Aries Systems Corporation

**Format for specific study types**<br><br>**Human Subject Research (involving human participants and/or tissue)**<br>• Give the name of the institutional review board or ethics committee that approved the study<br>• Include the approval number and/or a statement indicating approval of this research<br>• Indicate the form of consent obtained (written/oral) or the reason that consent was not obtained (e.g. the data were analyzed anonymously)<br><br>**Animal Research (involving vertebrate animals, embryos or tissues)**<br>• Provide the name of the Institutional Animal Care and Use Committee (IACUC) or other relevant ethics board that reviewed the study protocol, and indicate whether they approved this research or granted a formal waiver of ethical approval<br>• Include an approval number if one was obtained<br>• If the study involved *non-human primates*, add *additional details* about animal welfare and steps taken to ameliorate suffering<br>• If anesthesia, euthanasia, or any kind of animal sacrifice is part of the study, include briefly which substances and/or methods were applied<br><br>**Field Research**<br><br>Include the following details if this study involves the collection of plant, animal, or other materials from a natural setting:<br>• Field permit number<br>• Name of the institution or relevant body that granted permission	
**Data Availability**<br><br>Authors are required to make all data underlying the findings described fully available, without restriction, and from the time of publication. PLOS allows rare exceptions to address legal and ethical concerns. See the PLOS Data Policy and FAQ for detailed information.	Yes - all data are fully available without restriction

Powered by Editorial Manager® and ProduXion Manager® from Aries Systems Corporation

A Data Availability Statement describing where the data can be found is required at submission. Your answers to this question constitute the Data Availability Statement and **will be published in the article**, if accepted.<br><br>**Important:** Stating 'data available on request from the author' is not sufficient. If your data are only available upon request, select 'No' for the first question and explain your exceptional situation in the text box.<br><br>Do the authors confirm that all data underlying the findings described in their manuscript are fully available without restriction?	
**Describe where the data may be found in full sentences. If you are copying our sample text, replace any instances of XXX with the appropriate details.**<br><br>• If the data are **held or will be held in a public repository**, include URLs, accession numbers or DOIs. If this information will only be available after acceptance, indicate this by ticking the box below. For example: *All XXX files are available from the XXX database (accession number(s) XXX, XXX.*).<br>• If the data are all contained **within the manuscript and/or Supporting Information files**, enter the following: *All relevant data are within the manuscript and its Supporting Information files.*<br>• If neither of these applies but you are able to provide **details of access elsewhere**, with or without limitations, please do so. For example:<br><br>*Data cannot be shared publicly because of [XXX]. Data are available from the XXX Institutional Data Access / Ethics Committee (contact via XXX) for researchers who meet the criteria for access to confidential data.*<br><br>*The data underlying the results presented in the study are available from (include the name of the third party*	All relevant data are within the manuscript and its Supporting Information files.

and contact information or URL).<br>• This text is appropriate if the data are owned by a third party and authors do not have permission to share the data.<br><br>* typeset	
Additional data availability information:	Tick here if the URLs/accession numbers/DOIs will be available only after acceptance of the manuscript for publication so that we can ensure their inclusion before publication.

Powered by Editorial Manager® and ProduXion Manager® from Aries Systems Corporation

Manuscript Click here to access/download;Manuscript;Manuscript.docx

Factors influencing the participation of surrogate decision-makers for advanced cancer patients in advance care planning: A cross-sectional study

Zhihao Han[#1,2], Jingjing Su[#1], Guiyue Ma[1], Yong Fang[3], Linxia Tang[1], Xiaoqin Ma[*1]

[1] Department of Nursing, Zhejiang Chinese Medical University, Hangzhou City, Zhejiang Province, China

[2] Zhejiang Chinese Medical University First Clinical School of Medicine, Hangzhou City, Zhejiang Province, China

[3] Department of Nursing, Changsha Medical College, Changsha City, Hunan Province, China

[#] These authors contributed equally to this work

[*] Correspondence: 20041028@zcmu.edu.cn

Abstract

Aim: The aim of this study was to explore the factors influencing of surrogate decision-makers (SDMs) engagement in Advance Care Planning (ACP).

Design: A cross-sectional Study.

Method: A total of 285 SDMs of advanced cancer patients were recruited using convenience sampling methods. All eligible participants completed the structured questionnaires. Univariate analysis and correlation analyses were used to explore the relationship between different variables with the ACP engagement of SDMs. Factors significant previously in univariate or correlation analyses were included in the multiple linear regression analyses with $P < 0.05$.

Results: Eight factors in univariate analyses and three factors in correlation analyses with $P<0.05$ were taken into multiple linear regression analysis. Finally, seven variables revealed a statistically significant influence on ACP engagement of SDMs and were included in the multiple linear regression model.

Conclusion: This cross-sectional study demonstrated that multiple factors, including experiences of medical decision-making, education level, knowledge of ACP, treatment

expenditure, uncertainty of disease, social support and life orientation, significantly impact the engagement of SDMs in ACP.

Impact: Based on this study, we have identified some factors influencing the ACP engagement of SDMs, which might provide basic information for construction of the educational scheme of ACP in mainland China and help to improve SDMs engagement in clinical ACP practice in future.

Patient or public contribution: SDMs of advanced cancer patients were participated in this study, and assisted to assess the validation of the measurements.

Keywords: Advance care planning, Advance directives, Surrogate decision-makers, Decision-making, Advanced cancer patients

1 Introduction

Advance Care Planning (ACP) is increasingly implemented in oncology and other domains to enable individuals to make plans about their future health care and improve concordance of care with patients' goals. In 2017 the European Association for Palliative Care proposed a definition for ACP as a process that "enables individuals to define goals and preferences for future medical treatment and care, to discuss these goals and preferences with family and health-care providers, and to record and review these preferences if appropriate" [1]. The definition clearly states that ACP is much more than the completion of advance directives (ADs), which is an instructional directive or "living will" that allows a person to make decisions or state goals of care for future medical treatment and/or appoint a close affiliate to make decisions on their behalf [2]. AD can be an important component of the ACP process. While ACP is a broader process that includes discussions and planning, AD serves as a legal document that formalizes these preferences. The implementation of AD has been guaranteed in many countries and regions, such as England, United States, Austria, Germany. While, the practices of ADs were marked heterogeneity between countries of in a study of 2021, which showed that only 15 of the 28 European Union countries had developed specific rules on ADs, which are, if written, legally binding in 86% of cases [3]. In mainland China, the traditional Chinese culture deters the widespread adoption of ACP and there is a lack of laws and regulations to guarantee the application of ADs and ACP. In traditional Chinese culture, collectivist values and family-centered decision-making

often prioritize familial consensus over individual autonomy, which might go against the patients' own willingness. Additionally, discussing death and end-of-life care is considered to be inauspicious and remain something of taboos in Chinese culture, making it difficult to initiate ACP conversations. These cultural beliefs and practices create barriers to the mainland China.

2 Background

According to the latest data from the International Agency for Research on Cancer in 2022, there were close to 20 million new cases of cancer alongside 9.7 million deaths from cancer. China contributed with 24.1% of new cases and 26.8% of cancer-related deaths [4]. Many individuals in China are diagnosed with advanced stages of cancer due to delayed cancer screening and prevention efforts, and they have to face death and life-threatening complications during treatment [5]. Disease progression might result in them suddenly falling into unconsciousness or becoming incapable of expressing their own treatment preferences. In Chinese family, the family members always insist to save or sustain the individuals' lives. While, life-sustaining treatments may also expose individuals to grave suffering and reduce the quality of life [6]. To maintain individuals' autonomy and protect their right of self-determination, discussions about medical preferences must be conducted when individuals are still capable of decision-making.

ACP has emerged as a solution to this problem, enabling individuals to choose their preferences for medical treatment and care even when they are incapacitated [7]. ACP also encourage individuals and their families to participate in the shared decision-making discussion with healthcare professionals. This collaborative pattern helps surrogate decision makers (SDMs) understand the individual's health status, values and goals, easing decision-making burdens, and preventing conflict.

In Asian nursing cultures, collectivist values and family-centered decision-making often prioritize familial consensus over individual autonomy. Hierarchical respect for elders and physicians may limit open dialogue about end-of-life preferences, delegating ACP discussions to SDMs (usually family members). Filial piety norms encourage families to shield individuals from distressing prognoses, potentially delaying or avoiding ACP. However, shared responsibility among relatives can also foster collaborative planning. When individuals experience cognitive impairment, delirium

and other conditions of loss of decision-making capacity, family members are often deemed to be the primary decisions makers on their behalf. Therefore, most individuals in China would choose family members, such as spouses, children or parents, as SDMs [8] due to the family concept. Engagement of family members in the ACP process is critical component in the improvement of ACP practice.

Currently, most studies are mainly focused on individuals' engagement in ACP, such as the completion rate of ACP, or the engagement in the comprehensive ACP process measured by the ACP Engagement Survey [9-12]. However, there are few studies focused on the ACP engagement of SDMs in mainland China. The purpose of this study was to explore the influencing factors of the SDMs engagement in ACP. This study might provide some information for the construction of the educational scheme of ACP in mainland China and help to improve SDMs engagement in clinical ACP practice in future.

3 The Study

3.1 Aim

The aim of this study was to explore the factors influencing of ACP engagement of SDMs.

4 Methods

4.1 Study design

This study is a cross-sectional study. Data collection was conducted from February 2023 to July 2023 at First Affiliated Hospital of Zhejiang Chinese Medical University. This hospital is one of the largest tertiary hospitals of Zhejiang Province, China, in which oncology department is rated as a key discipline by National Administration of Traditional Chinese Medicine of the People's Republic of China. The questionnaires were verified after participants finished to decrease missing data or illegible.

4.2 Participants and selection criteria

Participants were recruited using convenience sampling methods. Participants were invited to take part in this survey under the guidance of well-trained investigators. The investigators explained in detail the purpose of this study and obtained informed

consent from all participants. All eligible participants completed the structured paper questionnaires by themselves or with the assist of professional investigators if needed. All questionnaires were collected and questionnaires with missing or illegible data would be excluded before analyzed.

The inclusion criteria for SDMs of advanced cancer patients were as follows: (1) 18 years old or above; (2) blood, marital, parental relationship with the advanced tumor patient (such as, parents, spouse, children, sibling etc.) and being appointed as a SDM by the patient through a formal written designation, in accordance with the ethical principles and clinical guidelines relevant to the protection of patients' rights and interests; (3) provision of informed consent and cooperation with the survey; (4) able to communicate and compliance to complete the questionnaire.

Based on the principles of sample size estimation in empirical methods [13], this study includes a total of 23 variables. Taking 5-10 times the number of observation variables, the sample size is estimated to be 115-230 participants. Meanwhile, considering a dropout rate of 20%, the final sample size range determined for this study is 138-276 participants.

4.3 Measurements

To identify the factors influencing the ACP engagement of SDMs of patients with advanced cancer, a structured questionnaire was designed in this study. Based on the results of previous study [14], factors were always divided into three dimensions, the individual, environmental, and behavioral dimensions. This study explored how individual and environmental factors influencing the ACP engagement of SDMs by the theory of triadic reciprocal determinism. Individual factors such as self-efficacy and outcome expectations mainly manifested as life orientation ability and uncertainty about illness. Environmental factors primarily manifested as the level of social support for SDMs, and behaviors involving the evaluation of SDMs' ACP engagement.

4.3.1 Socio-demographic and cancer characteristics

The Socio-demographic and cancer characteristics questionnaire was designed by the researchers. We chose 12 variables in the questionnaire, which might affect the SDMs' engagement of ACP in previous studies [8, 10, 15], including age, sex, education level, marital status, the number of family members of SDMs, annual income, relationship

with the advanced cancer patient, classification of patients' cancer, treatment expenditure, patients' activities of daily living (ADL) independence level, experiences of making medical decision and knowledge of ACP.

4.3.2 Chinese Version of 17-item Advance Care Planning Engagement Survey for Surrogate Decision Makers (ACP-17-SDM)

The Chinese Version of ACP-17-SDM was translated by Wang Tianhang et al [15] from the English version of ACP-17-SDM [16]. The ACP-17-SDM included 3 dimensions (serving as SDM, contemplation, and readiness) with17 items. Each item was rated with 5-Point Likert Scale ranging from 1 (not at all / never) to 5 (very much / always). The total score of ACP-17-SDM ranged from 17 to 85 points with higher scores indicating higher ACP engagement of SDMs. This Chinese Version of ACP-17-SDM was reliable to use with a total Cronbach's α coefficient of 0.915 in this study.

4.3.3 Chinese Version of Mishel Uncertainty in illness Scale-Family Member Form (MUIS-FM)

The levels of disease uncertainty in the family members of patients were evaluated with Chinese version of MUIS-FM which was translated and revised by Wenying Wang [17]. The C-MUIS-FM consisted of 4 dimensions (ambiguity, lack of clarity, lack of information, and unpredictability) covering 25 items. The total scores of C-MUIS-FM were ranging from 25 to 125 points with higher scores indicating higher level of disease uncertainty of family members. The total Cronbach's α coefficient of the C-MUIS-FM was 0.887 in this study.

4.3.4 Social support rating scale (SSRS)

The SSRS was revised by Shuiyuan Xiao [18], which was used to assess current level of social support. The scale consisted of 3 dimensions, including subjective social support, objective social support, and social support use. The total scores of SSRS ranged from 12 to 66 points, with higher scores indicating higher levels of social support. The total Cronbach's alpha coefficient of SSRS was 0.788 in this study.

4.3.5 Chinese version of Life orientation test-Revised (LOT-R)

This Chinese version of LOT-R was translated by Wengen Deng [19] from English version of LOT-R [20]. The LOT-R was mainly used to assess the disposition of

optimism or pessimism. The LOT-R was a single-dimensional self-assessment scale consisting of 10 items. Each item was rated with 5-Point Likert Scale ranging from 0 (strongly disagree) to 4 (strongly agree). The total scores of the LOT-R ranged from 0 to 24 points, the higher scores, and the higher level of optimism. In this study, the total Cronbach's α coefficient was 0.831.

4.4 Statistical Analysis

The Statistical Package for the Social Sciences (SPSS) software version 26.0 was used for data input and statistical analyses. The socio-demographic and the characteristics of cancer of their care recipients were described by frequency and percentage. The scores of questionnaires were described by mean ± standard deviation or median (Interquartile Range) for normal or non-normal distribution data. Univariate analyses were used to explore the difference of ACP engagement of SDMs in different categories of socio-demographic characteristics. Independent samples t-test or ANOVA test and Mann-Whitney U rank-sum test or Kruskal-Wallis H rank-sum test were used for normal or non-normal distribution data, respectively. Pearson or Spearman correlation analyses were used to explore the correlations of other variables, including the uncertainty of disease, social support and life orientation of participants, with ACP engagement of SDMs. Factors significant previously in univariate or correlation analyses were included in the multiple linear regression analyses with $P > 0.05$ as the removal standard. If we encounter problems such as missing data that affect data integrity during the data analysis process, we will delete the participant's data.

4.5 Ethical considerations

This study was approved by Zhejiang Chinese Medical University Ethics Committee on 16th February 2023 (20230216-5). All participants were voluntary to anonymously participate in this study. Full explanation of this study was provided to all participants, and written informed consent was obtained before the interviews.

5 Results

During this study, of the 285 questionnaires administered, 9 responses were excluded because of missing data or illegible. Thus, 276 responses were finally analyzed (effective response rate 96.0%).

5.1 Socio-demographic characteristics of the participants

The participants' socio-demographic and cancer characteristics were presented in Table 1. The participants were evenly distributed by age and sex. Most of the participants were married and lived with their family members. While, more than two-third (70.3% and 68.5%) of the participants had no experiences of medical decision-making or lacked knowledge of ACP. Most of the SDMs were the children (44.9%) or spouses (43.8%) of advanced cancer patients. Nearly two-third (63.4%) of the patients could take care of themselves in daily life.

Table 1 Socio-demographic characteristics of the participants (N=276)

**Socio-demographic characteristics**		**Frequency (n)**	**Percentage (%)**
**Age (years)**	18-40	98	35.5
	41-60	113	40.9
	≥61	65	23.6
**Sex**	Female	144	52.2
	Male	132	47.8
**Education status**	Primary school or less	46	16.7
	Junior high school	72	26.1
	Senior high school	57	20.7
	College	90	32.6
	Master degree or above	11	4.0
**Marital status**	Unmarried or widowed or divorced	37	13.4
	Married	239	86.6
**Experiences of making medical decision**	No	194	70.3
	Yes	82	29.7
**Knowledge of ACP**	Unknown	189	68.5
	Heard of ACP	55	19.9
	Partly known	28	10.1
	Known	4	1.4

**Treatment expenditure**	≤ ¥10,000 (USD$1370)	110	39.9
	≤ ¥30,000 (USD$4110)	120	43.5
	≤ ¥50,000 (USD$6850)	28	10.1
	>¥50,000 (USD$6850)	18	6.5
**Annual income**	≤ ¥10,000 (USD$1370)	112	40.6
	≤ ¥30,000 (USD$4110)	101	36.6
	≤ ¥50,000 (USD$6850)	30	10.9
	>¥50,000 (USD$6850)	33	12.0
**The number of family members of SDMs**	≤3	110	39.9
	4-5	121	43.8
	≥6	45	16.3
**Relationship with the advanced cancer patient**	Children	124	44.9
	Parents	19	6.9
	Spouse	121	43.8
	Siblings	6	2.2
	Grandchildren	6	2.2
**Classification of patients' cancer**	Digestive system tumors	112	40.6
	Hematologic cancers	34	12.3
	Genitourinary cancer	28	10.1
	Head and neck cancer	25	9.1
	Thoracic cancer	77	27.9
**Patient's ADL* independence level**	Dependent	24	8.7
	Partly dependent in daily life	77	27.9
	Independent in Daily life	62	22.5
	Completely dependent	113	40.9

* ADL: Activities of daily living

5.2 Factors associated with the ACP engagement of SDMs

Base on univariate analyses, the factors significant influencing the ACP engagement of SDMs were age, education level, experiences of making medical decision, knowledge of ACP, treatment expenditure, annual income, relationship with the advanced cancer

patient. While, there was no significant difference in ACP engagement of SDMs stratified by marital status, numbers of family members of SDMs, classification of patients' cancer and patient's independence level (Table 2). In addition, the correlation between the uncertainty of disease, social support, life orientation of participants with ACP engagement of SDMs were shown in Table 3. The ACP engagement of SDMs were negatively associated with uncertainty of disease (P<0.01), and positively associated with social support and life orientation of participants (both P<0.01).

Table 2 The difference of ACP engagement of SDMs with different socio-demographic and care recipients characteristics

**Characteristics**	**Subscales**			**Total** [mean±SD)/ median(IQR)]
	**Serving as SDM** [mean±SD)/ median(IQR)]	**Contemplation** [mean±SD)/ median(IQR)]	**Readiness** [mean±SD)/ median(IQR)]	
**Age (years)**				
18-40	25.10±3.63	11.00 (7.00)	19.00 (7.00)	55.50 (18.00)
41-60	23.38±5.25	10.00 (7.00)	17.00 (7.00)	49.00 (21.00)
≥61	21.46±5.76	7.00 (8.00)	14.00 (7.00)	43.00 (24.00)
F/H	10.996	5.605	16.536	16.604
P	**＜0.001**	0.061	**＜0.001**	**＜0.001**
**Sex**				
Female	22.51±4.93	9.00 (7.00)	16.00 (8.00)	47.50 (19.00)
Male	24.67±4.96	10.00 (8.00)	17.50 (9.00)	54.00 (22.00)
t/Z	-3.627	-0.557	-2.478	-2.678
P	**＜0.001**	0.577	**0.013**	**0.007**
**Education level**				
Primary school or less	18.85±4.77	7.00 (2.00)	13.00 (3.00)	40.74±9.02

Junior high school	21.38±4.64	7.00 (4.00)	15.00 (5.00)	45.33±10.73
Senior high school	24.93±3.63	12.00 (6.00)	19.05±4.38	55.44±10.61
College	27.00 (5.00)	13.00 (7.00)	20.00 (8.00)	57.83±10.55
Master degree or above	29.00 (2.00)	16.00 (5.00)	22.36±3.53	65.27±8.75
F/H	92.646	59.416	77.940	34.457
P	**＜0.001**	**＜0.001**	**＜0.001**	**＜0.001**
**Marital status**				
Unmarried or widowed or divorced	23.11±4.60	10.19±3.95	17.00 (9.00)	48.00 (22.00)
Married	24.00 (8.00)	10.00 (8.00)	17.00 (7.00)	49.00 (21.00)
Z	-0.831	-0.161	-0.284	-0.311
P	0.406	0.872	0.776	0.756
**Experiences of making medical decision**				
No	23.00 (6.00)	8.00 (6.00)	15.00 (6.00)	46.00 (15.00)
Yes	28.00 (4.00)	14.00 (5.00)	21.00±4.45	64.00 (12.00)
Z	-7.346	-6.780	-7.294	-7.852
P	**＜0.001**	**＜0.001**	**＜0.001**	**＜0.001**
**Knowledge of ACP**				
Unknown	21.90±4.78	8.00 (6.00)	15.90±4.13	46.00 (15.00)
Heard of ACP	28.00 (3.00)	14.00 (5.00)	21.25±3.94	64.00 (11.00)
Partly known	27.36±3.47	13.61±3.34	22.14±4.08	63.11±9.15
Known	26.50±4.93	10.75±4.27	17.50±5.80	54.75±14.18
F/H	70.245	59.818	36.639	82.403

P	**＜0.001**	**＜0.001**	**＜0.001**	**＜0.001**
**Treatment expenditure**				
≤ ¥10,000 (USD$1370)	22.40±4.52	7.00 (6.00)	15.00 (5.00)	43.00 (14.00)
≤ ¥30,000 (USD$4110)	25.00 (7.00)	11.50 (7.00)	18.50 (8.00)	53.72±12.98
≤ ¥50,000 (USD$6850)	25.50 (7.00)	12.00 (7.00)	19.18±4.91	54.36±11.71
>¥50,000 (USD$6850)	28.50 (5.00)	12.17±3.20	20.56±4.25	63.00 (12.00)
H	20.812	28.671	27.781	28.729
P	**＜0.001**	**＜0.001**	**＜0.001**	**＜0.001**
**Annual income**				
≤ ¥10,000 (USD$1370)	21.37±4.94	7.50（6.00）	14.50 (5.00)	43.50 (16.00)
≤ ¥30,000 (USD$4110)	24.00 (6.00)	11.00 (7.00)	18.00 (8.00)	52.00 (21.00)
≤ ¥50,000 (USD$6850)	27.50 (5.00)	12.50 (6.00)	18.40±4.60	55.70±12.45
>¥50,000 (USD$6850)	27.00 (3.00)	12.09±3.31	20.52±3.95	58.97±8.96
H	42.865	23.014	37.549	40.237
P	**＜0.001**	**＜0.001**	**＜0.001**	**＜0.001**
**The number of family members of SDMs**				
≤3	24.50 (8.00)	11.50 (8.00)	18.00 (9.00)	53.50 (24.00)
4-5	23.00 (7.00)	9.00 (7.00)	16.00 (7.00)	47.00 (19.00)
≥6	24.16±3.93	8.00 (8.00)	16.00 (7.00)	48.00 (20.00)

H	4.995	5.366	4.744	5.756
P	0.082	0.068	0.093	0.056
**Relationship with the advanced cancer patient**				
Children	24.69±4.15	11.00 (7.00)	18.00 (7.00)	54.00 (19.00)
Parents	21.37±4.11	7.74±2.88	13.00 (4.00)	44.21±9.38
Spouse	22.55±5.77	9.00 (8.00)	15.00 (8.00)	47.00 (24.00)
Siblings	26.17±2.93	10.50±4.46	18.83±4.79	55.50±11.36
Grandchildren	23.83±5.19	16.00 (8.00)	19.33±4.80	56.50±12.36
F/H	4.253	11.811	16.576	15.189
P	**0.002**	**0.019**	**0.002**	**0.004**
**Classification of patients' cancer**				
Digestive system tumors	23.00 (8.00)	10.00 (8.00)	17.00 (8.00)	51.38±12.55
Hematologic cancers	24.53±3.59	9.49±3.78	16.00 (7.00)	49.00 (19.00)
Genitourinary cancer	23.64±4.65	10.50 (9.00)	17.00 (11.00)	52.00 (27.00)
Head and neck cancer	23.64±5.83	8.00 (9.00)	16.36±4.89	46.00 (27.00)
Thoracic cancer	24.00 (9.00)	10.00 (7.00)	17.00 (9.00)	51.00 (24.00)
H	1.371	2.139	2.301	0.697
P	0.849	0.710	0.681	0.952
**Patients' ADL independence level**				
Dependent	25.50 (14.00)	12.50 (10.00)	17.46±5.70	51.25±16.50

Partly dependent in daily life	26.00 (9.00)	12.00 (8.00)	20.00 (10.00)	60.00 (23.00)
Independent in Daily life	23.42±4.74	10.00 (5.00)	16.00 (8.00)	48.00 (19.00)
Completely dependent	23.00 (6.00)	8.00 (7.00)	16.00 (6.00)	47.00 (15.00)
H	4.265	8.211	9.602	7.006
P	0.234	**0.042**	**0.022**	0.072

ADL: Activities of daily living; F: F value of ANOVA test, H: H value of Kruskal-Wallis H rank-sum test; t: t value of independent samples t-test; Z: Z value of Mann-Whitney U rank-sum test; P-values indicating significance at the 0.05 level are shown in bold.

Table 3 The correlation between the uncertainty of disease, social support, life orientation of participants with ACP engagement of SDMs

**Characteristics**	**Subscales**			**Total**
	**Serving as SDM**	**Contemplation**	**Readiness**	
Uncertainty of disease	-0.485**	-0.452**	-0.469**	-0.524**
Social support	0.514**	0.410**	0.501**	0.534**
Life orientation	0.356**	0.387**	0.401**	0.424**

**: P<0.01

5.3 Multiple linear regression analysis for factors associated with the ACP engagement of SDMs

Eight factors in univariate analysis and three factors in correlation analyses with P<0.05 were taken into multiple linear regression analysis. The stepwise regression method was performed to access the relationship between different variables. All independent variables were shown in Table 4, and the tolerance (TOL) of each variable in the model was greater than 0.1, and the variance inflation factor (VIF) was less than 5. Thus, it was considered that there was no obvious multicollinearity between each variable.

Table 4 Independent variables assignment in multiple linear regression analysis

Independent variable	Assignment
Age	1=18-40, 2=40-60, 3=≥61
Sex	1=Female, 2=Male
Education level	1= Primary school or less, 2= Junior high school, 3= Senior high school, 4= College, 5= Master degree or above
Experiences of medical decision-making	1=No, 2=Yes
Knowledge of ACP	1= Unknown, 2= Heard of ACP, 3= Partly known, 4= Known
Treatment expenditure	1=⩽ ¥10,000 (USD$1370), 2=⩽ ¥30,000 (USD$4110), 3=≤ ¥50,000 (USD$6850), 4=>¥50,000 (USD$6850)
Annual income	1=⩽ ¥10,000 (USD$1370), 2=⩽ ¥30,000 (USD$4110), 3=≤ ¥50,000 (USD$6850), 4=>¥50,000 (USD$6850)
Relationship with the advanced cancer patient	1= Children, 2= Parents, 3= Spouse, 4= Siblings, 5= Grandchildren
The uncertainty of disease	null
The social support	null
The life orientation	null

Finally, as shown in Table 5, only seven variables revealed statistically significant influences on ACP engagement of SDMs and those variables included in the multiple linear regression model could explain 56.7% of the total variation (Adjusted R^2=0.567; F=52.448; P＜0.001). Those variables, except uncertainty of disease, presented a strong positive association with ACP engagement of SDMs.

Table 5 Multiple linear regression analysis of independent variables in ACP engagement of SDMs

Variables	Regression coefficient	Standardized coefficient	t	P	TOL	VIF

Experiences of medical decision-making	6.144	0.224	4.934	**<0.001**	0.771	1.297
Education level	2.460	0.230	4.650	**<0.001**	0.491	2.035
Knowledge of ACP	1.989	0.116	2.387	**0.018**	0.649	1.541
Treatment expenditure	1.541	0.105	2.554	**0.011**	0.908	1.101
Uncertainty of disease	-0.196	-0.232	-5.051	**<0.001**	0.782	1.279
Social support	0.249	0.159	3.186	**0.002**	0.666	1.501
Life orientation	0.274	0.105	2.279	**0.023**	0.773	1.294

TOL: tolerance; VIF: variance inflation factor; P-values indicating significance at the 0.05 level are shown in bold.

6 Discussion

This study investigated the ACP engagement of SDMs of Chinese advanced cancer patients, which provided valuable insights for ACP adaptations in mainland China. To our knowledge, this study was the first large sample study focusing on the factors influencing ACP engagement of SDMs of different advanced cancer patients in mainland China. Currently, there were few studies related to ACP engagement of patients at the end life in mainland China due to the Chinese traditional culture which limited the spread and implement of ACP [15].

A total of 276 SDMs of advanced cancer patients were analyzed in this study, with balanced distribution of sex (52.2% female and 47.8% male). Most of participants were the children or spouse of the advanced cancer patients. Among the participants, SDMs younger than 60 years old were account for a large proportion, which were similar to the results of Wang's study [15]. Combined with the surrogate relationship with patients, we considered the following possibilities of this phenomenon. On one hand, most of advanced cancer patient were the elderly, and their children became the breadwinner of family which means their children would also take the role of SDMs. On the other hand, for some middle-aged cancer patients, their spouse might become the first choice of SDM because their children were too young and their parents were too old to make decision. In our study, participants with 18 to 40 years old have the highest level of ACP engagement among the different age groups and this is different to findings by Liu which showed that older SDMs had higher ACP engagement [21]. This difference

might be attributed the different population characteristics of care recipients, and Liu's study recruited community-dwelling elderly patients with chronic diseases.

Experiences of medical decision-making was significant associated with the engagement of ACP, and participants with experiences showed more positive engagement of ACP. However, only 29.7% of the participants had experiences of medical decision-making in this study, which might attribute to the limited implementation of the doctor-patient-surrogate decision-making model in mainland China. And it would hinder SDMs from fully obtaining the patient's medical information [22-24]. A recent study presented that SDMs with experiences of decision-making would be more deliberate when making medical decisions due to the knowledge of disease trajectory [25].

The univariate analyses showed that education level was positively correlation with ACP engagement. And the results was similar to the study of Keam et al [26], which also showed that participants with higher education level had a more positive ACP engagement than those with lower education level. Participants with high education level tended to have a strong sense of autonomy, and consider the value of life and future medical intention when actively seeking disease information [27, 28]. Conversely, participants with low education level might lack knowledge to support their thinking, although they want to express their wishes [29]. Participants had knowledge of ACP presented a more positive attitude than others, which is consistent with the study of Park et al [30]. A full understanding of new things is the prerequisite for participation. Therefore, it is important for government, media and hospitals to establish theoretical frameworks to disseminate accurate information about ACP and strengthen the public's knowledge of ACP [31]. To enhance the engagement of SDMs with restricted education in ACP discussions, it is essential to employ plain and straightforward language to elucidate the importance of ACP and provide them with easily digestible written resources and visual aids. Moreover, offering training programs and securing support from healthcare professionals can also help SDMs better understand the patient's requirements and preferences, thus increasing their active participation in ACP discussions.

This study showed that the treatment expenditure played an important role in making medical decisions by SDMs for advanced cancer patients, and the high cost of treatment

expenditure often puts SDMs under great economic pressure [32, 33], which in turn rendered them more predisposed to contemplate less invasive medical care approaches. This financial burden further motivated to participate in ACP discussions. The composition treatment expenditure was similar to annual income, which indicated the treatment expenditure of advanced cancer would occupy the most of annual income, bringing huge economic burden to their families [34-36]. It is recommended that medical staff should comprehensively consider the treatment needs and economic status of patients and their family, assisting them to weigh the pros and cons of different treatment options and provide personalized medical decision-making recommendations [37]. Additionally, policymakers should consider implementing measures to alleviate the financial burden of cancer treatment, such as expanding insurance coverage and establishing financial assistance programs.

This study showed that the engagement of ACP was negatively correlated to the SDMs' sense of disease uncertainty, and positively correlated to the SDMs' social support and life orientation ability. Firstly, according to the uncertainty in illness theory, uncertainty arises when individuals are unable to establish an adaptive cognitive framework for related events [38]. Doctors and healthcare professionals play a vital role in facilitating communication between the patients and SDMs by offering support and guidance throughout these stages, especially for SDMs who have no medical knowledge. Secondly, social support, referring to the material and spiritual support that individuals obtain from family or other social networks [39, 40]. However, the result was inconsistent to a study investigating the factor influencing ACP engagement of SDMs of patients with hematologic malignancies [15]. However, there is also a study with similar conclusions to this one: participants with high levels of social support showed more active involvement in ACP and higher quality ACP completion [41]. Thirdly, many studies have shown that individuals under good emotional state will result in more altruistic behaviors [42-44]. Optimistic SDMs often actively seek help from others to find solutions when encountering difficulties. Therefore, SDMs with strong life orientation ability are more willing to participate in the discussion of patients' treatment decisions and have a more positive attitude towards ACP.

Patients play a central role in the development and implementation of the entire ACP, and it is crucial to facilitate communication between patients and SDMs. Healthcare providers should play an active role in facilitating communication between patients and

SDMs. This includes providing clear and comprehensive information about ACP, its benefits, and the importance of the patient's active participation. Educational sessions and materials can help both patients and SDMs understand the process and their respective roles. Establishing a trusting relationship between patients, SDMs, and healthcare providers is essential. This can be achieved through open and empathetic communication, where patients feel comfortable expressing their wishes and concerns. Each patient and family is unique, and the approach to ACP should be tailored to their specific needs and circumstances. Healthcare providers should be sensitive to cultural, religious, and personal beliefs and preferences, and adapt their communication and support accordingly.

Our study highlights the importance of SDMs in ACP for advanced cancer patients. Future practice should focus on providing healthcare providers with enhanced training on how to effectively communicate with SDMs and patients about ACP. This includes training on cultural sensitivity, communication techniques, and strategies to overcome barriers to ACP discussions. ACP should be integrated into routine care for advanced cancer patients. Healthcare providers should make it a standard practice to discuss ACP with patients and their SDMs early in the disease trajectory. This can help ensure that patients' preferences and values are respected and that their care aligns with their wishes. In addition, future research should focus on longitudinal studies to better understand the long - term impact of ACP on patient outcomes and family satisfaction. Meanwhile, there is a need for more intervention studies to test the effectiveness of different ACP strategies.

Several limitations of this study should be acknowledged. Firstly, recruiting a convenience sample from a single cancer center may limit generalizability to national target populations. Secondly, the individual perspectives of participants, such as their worldviews, life outlooks, and values, were not included in this study, which limited the comprehensiveness of our conclusions. Further studies with a large, national-wide sample and more comprehensive factors are needed to substantiate our findings.

7 Conclusion

This cross-sectional study demonstrated that multiple factors, including experiences of medical decision-making, education level, knowledge of ACP, treatment expenditure,

uncertainty of disease, social support and life orientation, significantly impact the engagement of SDMs in ACP. In conclusion, this study provides valuable insights into the factors influencing ACP participation among surrogate decision-makers for advanced cancer patients. Future research should further explore the specific mechanisms underlying these factors and develop targeted interventions to improve ACP engagement, ultimately enhancing the quality of end-of-life care for patients with advanced cancer.

Author Contributions

Conceptualization: Xiaoqin Ma.

Data curation: Zhihao Han.

Formal analysis: Zhihao Han.

Supervision: Xiaoqin Ma.

Investigation: Guiyue Ma, Linxia Tang, Yong Fang.

Validation: Jingjing Su.

Writing– original draft: Zhihao Han, Guiyue Ma.

Writing– review & editing: Jingjing Su, Xiaoqin Ma.

Conflict Statement

No conflict of interest has been declared by the authors.

Funding Information

This project did not receive any grant from funding agencies in the public, commercial, or not-for-profit sectors.

Acknowledgments

This research was not funded by any public, commercial, or nonprofit agency. No competing financial interests exist. The authors thank all members of the cancer center for their help in this study.

Data Availability Statement

Data available on request from the authors.

REFERENCES

1. Rietjens JAC, Sudore RL, Connolly M, van Delden JJ, Drickamer MA, Droger M, et al. Definition and recommendations for advance care planning: an international consensus supported by the European Association for Palliative Care. The Lancet Oncology. 2017; 18(9):e543-e51. https://doi.org/10.1016/s1470-2045(17)30582-x PMID: 28884703.
2. Ribeiro C, Pamplona P, Simonds AK. Advance care planning in patients with respiratory failure. European respiratory review: an official journal of the European Respiratory Society. 2024; 33(174). https://doi.org/10.1183/16000617.0120-2024 PMID: 39537243.
3. Porcar Rodado E, Peral Sanchez D, Gisbert Grifo M. Advance Directives. Comparison of current legislation within the European Union. Spanish Journal of Legal Medicine. 2021; 47(2):66-73. https://doi.org/10.1016/j.remle.2020.05.011.
4. Bray F, Laversanne M, Sung H, Ferlay J, Siegel RL, Soerjomataram I, et al. Global cancer statistics 2022: GLOBOCAN estimates of incidence and mortality worldwide for 36 cancers in 185 countries. CA: a cancer journal for clinicians. 2024; 74(3):229-63. https://doi.org/10.3322/caac.21834 PMID: 38572751.
5. Canny A, Mason B, Boyd K. Public perceptions of advance care planning (ACP) from an international perspective: a scoping review. BMC palliative care. 2023; 22(1):107. https://doi.org/10.1186/s12904-023-01230-4 PMID: 37507777.
6. Lu Y, Gu Y, Yu W. Hospice and Palliative Care in China: Development and Challenges. Asia-Pacific journal of oncology nursing. 2018; 5(1):26-32. https://doi.org/10.4103/apjon.apjon_72_17 PMID: 29379830.
7. Barry HC. Advance Care Planning Increases Execution of Advance Directives and Surrogate Decision-Maker Assignment. American family physician. 2018; 98(11):682-683 PMID: 30485037.
8. Ni P, Zhou J, Wang ZX, Nie R, Phillips J, Mao J. Advance directive and end-of-life care preferences among nursing home residents in Wuhan, China: a cross-sectional study. Journal of the American Medical Directors Association. 2014; 15(10):751-56. https://doi.org/10.1016/j.jamda.2014.06.006 PMID: 25066002.
9. Bravo G, Dubois MF, Wagneur B. Assessing the effectiveness of interventions to promote advance directives among older adults: a systematic review and multi-level analysis. Social science & medicine (1982). 2008; 67(7):1122-32. https://doi.org/10.1016/j.socscimed.2008.06.006 PMID: 18644667.
10. Tamayo-Velázquez MI, Simón-Lorda P, Villegas-Portero R, Higueras-Callejón C, García-Gutiérrez JF, Martínez-Pecino F, et al. Interventions to promote the use of advance directives: an overview of systematic reviews. Patient education and counseling. 2010; 80(1):10-20. https://doi.org/10.1016/j.pec.2009.09.027 PMID: 19879090.
11. Sudore RL, Heyland DK, Barnes DE, Howard M, Fassbender K, Robinson CA, et al. Measuring Advance Care Planning: Optimizing the Advance Care Planning Engagement Survey. Journal of pain and symptom management. 2017; 53(4):669-81.e668. https://doi.org/10.1016/j.jpainsymman.2016.10.367 PMID: 28042072.

12. Sudore RL, Stewart AL, Knight SJ, McMahan RD, Feuz M, Miao Y, et al. Development and validation of a questionnaire to detect behavior change in multiple advance care planning behaviors. PLOS One. 2013; 8(9):e72465. https://doi.org/10.1371/journal.pone.0072465 PMID: 24039772.
13. Ni P, Chen JL, Liu N. Sample size estimation in quantitative research in nursing, Chinese Journal of Nursing, 45(2010) 378-80.
14. Bandura A. Social cognitive theory: an agentic perspective. Annu Rev Psychol. 2001; 52:1-26. https://doi.org/10.1146/annurev.psych.52.1.1 PMID: 11148297.
15. Wang TH, Wang W, Shen WT, Shi BX, Participation in advance care planning and associated factors among surrogate decision makers of patients with hematologic malignancies, Chinese general practice, 25 (2022) 859-66.
16. Van Scoy LJ, Day AG, Howard M, Sudore R, Heyland DK. Adaptation and Preliminary Validation of the Advance Care Planning Engagement Survey for Surrogate Decision Makers. Journal of pain and symptom management. 2019; 57(5):980-88.e989. https://doi.org/10.1016/j.jpainsymman.2019.01.008 PMID: 30684633.
17. Wang WY, Wang S, Xia DS, Cui GP, Wang CM, Study on reliability and validity of Chinese version of Mishel uncertainty in illness scale family member form, Chinese nursing research, 26 (2012) 662-63.
18. Xiao SY, Theory and research applications of the Social Support Rating Scale, Journal of clinical psychiatry, (1994) 98-100.
19. Deng WG, Wang P, Liu SS, Examining the issues and solutions for testing the dimensions of LOT and LOT-R using traditional methods, Journal of Shandong Normal University (Natural Science Edition), 24 (2009) 51-54. https://doi.org/10.3969/j.issn.1001-4748.2009.04.015.
20. Scheier MF, Carver CS, Bridges MW. Distinguishing optimism from neuroticism (and trait anxiety, self-mastery, and self-esteem): a reevaluation of the Life Orientation Test. Journal of personality and social psychology. 1994; 67(6):1063-78. https://doi.org/10.1037//0022-3514.67.6.1063 PMID: 7815302.
21. Liu X, Chen H, Zhang L, Zhang Q, Feng T, Liu D. Advance Care Planning Engagement Among Family Members of Community-Dwelling Elderly Patients With Chronic Diseases in China: A Mixed-Methods Study. Journal of hospice and palliative nursing : JHPN : the official journal of the Hospice and Palliative Nurses Association. 2022; 24(2):e26-e34. https://doi.org/10.1097/njh.0000000000000829 PMID: 35045050.
22. Chen C, Cheng G, Chen X, Yu L. Information disclosure to cancer patients in Mainland China: A meta-analysis. Psycho-oncology. 2023; 32(3):342-55. https://doi.org/10.1002/pon.6085 PMID: 36582008.
23. Hahne J, Liang T, Khoshnood K, Wang X, Li X. Breaking bad news about cancer in China: Concerns and conflicts faced by doctors deciding whether to inform patients. Patient education and counseling. 2020; 103(2):286-91. https://doi.org/10.1016/j.pec.2019.08.022 PMID: 31455567.
24. Xu H, Yuan M. Family roles in informed consent from the perspective of young Chinese doctors: a questionnaire study. BMC medical ethics. 2024; 25(1):2. https://doi.org/10.1186/s12910-023-00999-6 PMID: 38172849.
25. Bakke BM, Feuz MA, McMahan RD, Barnes DE, Li B, Volow AM, et al. Surrogate Decision Makers Need Better Preparation for Their Role: Advice from Experienced

Surrogates. Journal of palliative medicine. 2022; 25(6):857-63. https://doi.org/10.1089/jpm.2021.0283 PMID: 35076297.
26. Keam B, Yun YH, Heo DS, Park BW, Cho CH, Kim S, et al. The attitudes of Korean cancer patients, family caregivers, oncologists, and members of the general public toward advance directives. Supportive care in cancer: official journal of the Multinational Association of Supportive Care in Cancer. 2013; 21(5):1437-44. https://doi.org/10.1007/s00520-012-1689-z PMID: 23262809.
27. Wu F, Zhuang Y, Chen X, Wen H, Tao W, Lao Y, et al. Decision-making among the substitute decision makers in intensive care units: An investigation of decision control preferences and decisional conflicts. Journal of advanced nursing. 2020; 76(9):2323-35. https://doi.org/10.1111/jan.14451 PMID: 32538477.
28. Hopp FP. Preferences for surrogate decision makers, informal communication, and advance directives among community-dwelling elders: results from a national study. The Gerontologist. 2000; 40(4):449-57. https://doi.org/10.1093/geront/40.4.449 PMID: 10961034.
29. Rwabihama JP, Belmin J, Rakotoarisoa R, Hagege M, Audureau E, Benzengli H, et al. Promoting patients' rights at the end of life in a geriatric setting in France: The healthcare professionals' level of knowledge about surrogate decision-makers and advance directives. Patient education and counseling. 2020; 103(7):1390-98. https://doi.org/10.1016/j.pec.2020.01.020 PMID: 32070651.
30. Park HY, Kim YA, Sim JA, Lee J, Ryu H, Lee JL, et al. Attitudes of the General Public, Cancer Patients, Family Caregivers, and Physicians Toward Advance Care Planning: A Nationwide Survey Before the Enforcement of the Life-Sustaining Treatment Decision-Making Act. Journal of pain and symptom management. 2019; 57(4):774-82. https://doi.org/10.1016/j.jpainsymman.2018.12.332 PMID: 30593911.
31. Lin Goh SS. Singapore Takes Six Steps Forward in 'The Quality of Death Index' Rankings. Asia-Pacific journal of oncology nursing. 2018; 5(1):21-25. https://doi.org/10.4103/apjon.apjon_66_17 PMID: 29379829.
32. Jeong W, Kim S, Kim H, Park EC, Jang SI. Effects of life-sustaining treatment plans on healthcare expenditure and healthcare utilization. BMC health services research. 2023; 23(1):1236. https://doi.org/10.1186/s12913-023-10235-x PMID: 37950202.
33. Longo CJ, Fitch MI, Loree JM, Carlson LE, Turner D, Cheung WY, et al. Patient and family financial burden associated with cancer treatment in Canada: a national study. Supportive care in cancer: official journal of the Multinational Association of Supportive Care in Cancer. 2021; 29(6):3377-86. https://doi.org/10.1007/s00520-020-05907-x PMID: 33403399.
34. Xu J, Wang J, Liu R, Xing J, Su L, Yu F, et al. Mental health inpatient treatment expenditure trends in China, 2005-2012: evidence from Shandong. The journal of mental health policy and economics. 2014; 17(4):173-82 PMID: 25599281.
35. Yao A, Shen X, Chai J, Cheng J, Liu R, Feng R, et al. Characteristics and implications of insurance-reimbursed inpatient care for gastric and oesophageal cancers in Anhui, China. International health. 2021; 13(5):446-55. https://doi.org/10.1093/inthealth/ihaa092 PMID: 33210133.
36. Fu W, Shi J, Zhang X, Liu C, Sun C, Du Y, et al. Effects of cancer treatment on household impoverishment: a multicentre cross-sectional study in China. BMJ open. 2021; 11(6):e044322. https://doi.org/10.1136/bmjopen-2020-044322 PMID: 34193481.

37. van den Heuvel L, Meinders MJ, Post B, Bloem BR, Stiggelbout AM. Personalizing decision-making for persons with Parkinson's disease: where do we stand and what to improve? Journal of neurology. 2022; 269(7):3569-78. https://doi.org/10.1007/s00415-022-10969-4 PMID: 35084559.
38. Mishel MH. Reconceptualization of the uncertainty in illness theory. Image--the journal of nursing scholarship. 1990; 22(4):256-62. https://doi.org/10.1111/j.1547-5069.1990.tb00225.x PMID: 2292449.
39. Usta YY. Importance of social support in cancer patients. Asian Pacific journal of cancer prevention : APJCP. 2012; 13(8):3569-72. https://doi.org/10.7314/apjcp.2012.13.8.3569 PMID: 23098436.
40. Hupcey JE. Clarifying the social support theory-research linkage. Journal of advanced nursing. 1998; 27(6):1231-41. https://doi.org/10.1046/j.1365-2648.1998.01231.x PMID: 9663875.
41. Arch JJ, Bright EE, Finkelstein LB, Fink RM, Mitchell JL, Andorsky DJ, et al. Anxiety and Depression in Metastatic Cancer: A Critical Review of Negative Impacts on Advance Care Planning and End-of-Life Decision Making With Practical Recommendations. JCO oncology practice. 2023; 19(12):1097-08. https://doi.org/10.1200/op.23.00287 PMID: 37831973.
42. Feng L, Zhang L, Zhong H. Perceived Parenting Styles and Mental Health: The Multiple Mediation Effect of Perfectionism and Altruistic Behavior. Psychology research and behavior management. 2021; 14:1157-70. https://doi.org/10.2147/prbm.S318446 PMID: 34377038.
43. Brethel-Haurwitz KM, Stoianova M, Marsh AA. Empathic emotion regulation in prosocial behaviour and altruism. Cognition & emotion. 2020; 34(8):1532-48. https://doi.org/10.1080/02699931.2020.1783517 PMID: 32576078.
44. Barasch A, Levine EE, Berman JZ, Small DA. Selfish or selfless? On the signal value of emotion in altruistic behavior. Journal of personality and social psychology. 2014; 107(3):393-413. https://doi.org/10.1037/a0037207 PMID: 25133723.

Click here to access/download
Supporting Information
Supporting Information.xlsx

Click here to access/download
Supporting Information
supporting file.pdf

Factors influencing the participation of surrogate decision-makers for advanced cancer patients in advance care planning: A cross-sectional study

Zhihao Han[#1,2], Jingjing Su[#12], Guiyue Ma[12], Yong Fang[3], Linxia Tang[12], Xiaoqin Ma[*12]

~~[1] Zhejiang Chinese Medical University First Clinical School of Medicine, Hangzhou City, Zhejiang Province, China~~

[12] Department of Nursing, Zhejiang Chinese Medical University, Hangzhou City, Zhejiang Province, China

[21] Zhejiang Chinese Medical University First Clinical School of Medicine, Hangzhou City, Zhejiang Province, China

[3] Department of Nursing, Changsha Medical College, Changsha City, Hunan Province, China

[#] These authors contributed equally to this work

[*] Correspondence: 20041028@zcmu.edu.cn

Abstract

Aim: The aim of this study was to explore the factors influencing of surrogate decision-makers (SDMs) engagement in Advance Care Planning (ACP).

Design: A cross-sectional Study.

Method: A total of 285 SDMs of advanced cancer patients were recruited using convenience sampling methods. All eligible participants completed the structured questionnaires. Univariate analysis and correlation analyses were used to explore the relationship between different variables with the ACP engagement of SDMs. Factors significant previously in univariate or correlation analyses were included in the multiple linear regression analyses with P <0.05.

Results: Eight factors in univariate analyses and three factors in correlation analyses with P<0.05 were taken into multiple linear regression analysis. Finally, seven variables revealed a statistically significant influence on ACP engagement of SDMs and were included in the multiple linear regression model.

Conclusion: This cross-sectional study demonstrated that multiple factors, including experiences of medical decision-making, education level, knowledge of ACP, treatment expenditure, uncertainty of disease, social support and life orientation, significantly impact the engagement of SDMs in ACP.~~Our study suggested that some interventions should be implemented to strengthen the self-efficacy of ACP engagementwillingness of the SDMs.surrogate decision-makers to participate in ACP. The dissemination of ACP concepts should be strengthened, and related legislation should be established to provide support for ACP engagement in mainland China.~~

Impact: Based on this study, we have identified some factors influencing the ACP engagement of ~~SMDs~~SDMs, which might provide basic information for construction of the educational scheme of ACP in mainland China and help to improve SDMs engagement in clinical ACP practice in future.

Patient or public contribution: SDMs of advanced cancer patients were participated in this study, and assisted to assess the validation of the measurements.

Keywords: Advance care planning, Advance directives, Surrogate decision-makers, Decision-making, Advanced cancer patients

1 Introduction

Advance Care Planning (ACP) is increasingly implemented in oncology and other domains to enable individuals to make plans about their future health care and improve concordance ~~consistency~~ of care with patients' goals. In 2017 the European Association for Palliative Care proposed a definition for ACP as a process that "enables individuals to define goals and preferences for future medical treatment and care, to discuss these goals and preferences with family and health-care providers, and to record and review these preferences if appropriate" [1]. The definition clearly states that ACP is much more than the completion of advance directives (ADs), which is an instructional directive or "living will" that allows a person to make decisions or state goals of care for future medical treatment and/or appoint a close affiliate to make decisions on their behalf [2]. AD can be an important component of the ACP process. While ACP is a broader process that includes discussions and planning, AD serves as a legal document that formalizes these preferences.~~ACP is the discussions surrounding the patients' wishes and care and the AD is the formal document.~~ The implementation of AD has

Formatted: Font: Not Bold

Formatted: Font: Not Bold

been guaranteed in many countries and regions, such as EnglandUnited Kingdom, United States, Austria, Germany, etc. While, the practices of ADs were marked heterogeneity between countries of in a study of 2021, which showed that only 15 of the 28 European Union countries had developed specific rules on ADs, which are, if written, legally binding in 86% of cases [3]. In mainland China, the traditional Chinese culture deters the widespread adoption of ACP and there is a lack of laws and regulations to guarantee the application of ADs and ACP. In mainland China, In traditional Chinese culture, collectivist values and family-centered decision-making often prioritize familial consensus over individual autonomytraditional Chinese culture, which might go against the patients'’ own willingness. significantly influences the implementation of ACP. One key factor is filial piety, which emphasizes the importance of family and respect for elders. This cultural norm often leads to family-centered decision-making, where medical decisions are made collectively by family members rather than by individuals themselves. Additionally, discussing aboutdeath and end-of-life care are considered to be inauspicious and remain something of taboos in Chinese culture. are s, making it difficult to initiate ACP conversations. These cultural beliefs and practices create barriers to the widespread adoption of ACPmainland China. Therefore, AD and ACP in mainland China have not been widely adopted due to the influence of traditional Chinese culture, and there is a lack of laws and regulations to guarantee the application of ADs and ACP.

2 Background

According to the latest data from the International Agency for Research on Cancer in 2022, there were close to 20 million new cases of cancer alongside 9.7 million deaths from cancer. China contributed with 24.1% of new cases and 26.8% of cancer-related deaths [4]. Many patientindividuals in China are diagnosed with advanced stages of cancer due to delayed cancer screening and prevention efforts, and they have to face death and life-threatening complications during treatment [5]. Disease progression might result in them suddenly falling into unconsciousness or becoming incapable of expressing their own treatment preferences. In Chinese family, the family members always insist to save or sustain the patientindividuals' lives. While, life-sustaining treatments may also expose patientindividuals to grave suffering and reduce the quality of life [6]. To maintain patientindividuals' autonomy and protect their right of self-

determination, discussions about medical preferences must be conducted when ~~patient~~individuals are still capable of decision-making.

ACP has emerged as a solution to this problem, enabling ~~patient~~individuals to choose their preferences for medical treatment and care even when they are incapacitated [7]. ACP also encourage ~~patient~~individuals and their families to participate in the shared decision-making discussion with healthcare professionals. This collaborative pattern helps surrogate decision makers (SDMs) understand the ~~patient~~individual's health status, values and goals, easing decision-making burdens, and preventing conflict.

In Asian nursing cultures, collectivist values and family-centered decision-making often prioritize familial consensus over individual ~~patient~~ autonomy. Hierarchical respect for elders and physicians may limit open dialogue about end-of-life preferences, delegating ACP discussions to SDMs (usually family members). Filial piety norms encourage families to shield ~~patient~~individuals from distressing prognoses, potentially delaying or avoiding ACP. However, shared responsibility among relatives can also foster collaborative planning. When ~~patient~~individuals experience cognitive impairment, delirium and other conditions of loss of decision-making capacity, family members are often deemed to be the primary ~~critical~~ decisions makers on their behalf. ~~Additionally, in traditional Chinese cultural contexts, familial collective needs often supersede individual preferences.~~ Therefore, most ~~patient~~individuals in China would choose family members, such as spouses, children or parents, as SDMs~~surrogate decision makers~~ [8] due to the family concept. Engagement of family members in the ACP process is critical component in the improvement of ACP practice.

Currently, most studies are mainly focused on ~~patient~~individuals' engagement in ACP, such as the completion ~~compliance~~ rate of ACP, or the engagement in the comprehensive ACP process measured by the ACP Engagement Survey [9-12]. However, there are few studies focused on the ACP engagement of SDMs in mainland China. The purpose of this study was to explore the influencing factors of the SDMs engagement in ACP. This study might provide some information for the construction of the educational scheme of ACP in mainland China and help to improve SDMs engagement in clinical ACP practice in future.

3 The Study

3.1 Aim

The aim of this study was to explore the factors influencing of ACP engagement of SDMs. This study might provide some information for the construction of the educational scheme of ACP in mainland China and help to improve SDMs engagement in clinical ACP practice in future.

4 Methods

4.1 Study design

This study is a cross-sectional study. This dData collectionstudy was conducted from February 2023 to July 2023 at First Affiliated Hospital of Zhejiang Chinese Medical University. This hospital is one of the largest tertiary hospitals of Zhejiang Province, China, in which oncology department is rated as a key discipline by National Administration of Traditional Chinese Medicine of the People's Republic of China. Data collection and evaluation was conducted from February 2023 to January 2024. The questionnaires were verified after participants finished to decrease missing data or illegible. Besides, double entry data recording was used to avoid the data error.

Participants were recruited using convenience sampling methods. Participants were invited to take part in this survey under the guidance of well-trained investigators. The investigators explained in detail the purpose of this study and obtained informed consent from all patients. All eligible participants completed the structured questionnaires by themselves or with the assist of professional investigators.

Data collection and evaluation was conducted from February 2023 to January 2024. The questionnaires were verified after participants finished to decrease missing data or illegible. Besides, double entry data recording was used to avoid the data error.

4.2 Participants and selection criteria

Participants were recruited using convenience sampling methods. Paper questionnaires were used for data collection during the research process. Participants were invited to take part in this survey under the guidance of well-trained investigators. The investigators were hospital nurses who were responsible for explaining the study to the

SDMs, and responsible for recycling and checking the paper questionnaires. The investigators were available to assist participants during the questionnaire filling process if they needed it, but most participants completed the questionnaire independently. Participants were invited to take part in this survey under the guidance of well-trained investigators. The investigators explained in detail the purpose of this study and obtained informed consent from all participants patients. All eligible participants completed the structured paper questionnaires by themselves or with the assist of professional investigators if needed. All questionnaires were collected and questionnaires with missing or illegible data would be excluded before analyzed.

The inclusion criteria for SDMs of advanced cancer patients were as follows: (1) 18 years old or above; (2) blood, marital, parental relationship with the advanced tumor patient (such as, parents, spouse, children, sibling etc.) and being appointed as a SDM by the patient through a formal written designation, in accordance with the ethical principles and clinical guidelines relevant to the protection of patients' rights and interests; and appointment as a SDM by the patient according to the relevant regulations; (3) provision of informed consent and cooperation with the survey; (4) able to communicate and compliance to complete the questionnaire.

Based on the principles of sample size estimation in empirical methods [13], this study includes a total of 23 variables. Taking 5-10 times the number of observation variables, the sample size is estimated to be 115-230 participants. Meanwhile, considering a dropout rate of 20%, the final sample size range determined for this study is 138-276 participants.

4.3 Measurements

To identify the factors influencing the ACP engagement of SDMs of patients with advanced cancer, a structured questionnaire was designed in this study. Based on the results of previous studiesy [14], factors were always divided into three dimensions, the individual, environmental, and behavioral dimensions. This theory of triadic reciprocal determinism proposed a dynamic relationship model among the three dimensions [14]. This study explored how individual and environmental factors influencing the ACP engagement of SDMs by the theory of triadic reciprocal determinism. Individual factors such as self-efficacy and outcome expectations mainly manifested as life orientation ability and uncertainty about illness. Environmental factors primarily manifested as the

level of social support for SDMs, and behaviors involving the evaluation of SDMs' ACP engagement.

4.3.1 Socio-demographic and cancer characteristics

The Socio-demographic and cancer characteristics questionnaire was designed by the researchers. We chose 12 variables in the questionnaire, which might affect the SDMs' engagement of ACP in previous studies [8, 10, 15], including age, gendersex, education level, marital status, the number of family members of SDMsnumbers of family members, annual income, relationship with the advanced cancer patienttype of surrogate relationship, classification of patients' cancer, treatment expenditure, patients' activities of daily living (ADL) independence levelpatients' independence level, experiences of making medical decision and knowledge of ACP. The study collected participants' biological sex information through self-reported medical records, which was limited to male and female categories. We acknowledge that gender identity encompasses a broader sociocultural spectrum beyond this binary classification.

4.3.2 Chinese Version of 17-item Advance Care Planning Engagement Survey for Surrogate Decision Makers (ACP-17-SDM)

The Chinese Version of ACP-17-SDM was translated by Wang Tianhang et al [15] from the English version of ACP-17-SDM [16]. The ACP-17-SDM included 3 dimensions (serving as SDM, contemplation, and readiness) with17 items. Each item was rated with 5-Point Likert Scale ranging from 1 (not at all / never) to 5 (very much / always). The total score of ACP-17-SDM ranged from 17 to 85 points with higher scores indicating higher ACP engagement of SDMs. This Chinese Version of ACP-17-SDM was reliable to use with a total Cronbach's α coefficient of 0.915 in this study.

4.3.3 Chinese Version of Mishel Uncertainty in illness Scale-Family Member Form (MUIS-FM)

The levels of disease uncertainty in the family members of patients were evaluated with Chinese version of MUIS-FM which was translated and revised by Wenying Wang [17]. The C-MUIS-FM consisted of 4 dimensions (ambiguity, lack of clarity, lack of information, and unpredictability) covering 25 items. Each item was rated with 5-Point Likert Scale ranging from 1 (strongly disagree) to 5 (strongly agree) except item 6, 9, 17, 21, 23, 24, and 25, which were reverse rated. The total scores of C-MUIS-FM were

ranging from 25 to 125 points with higher scores indicating higher level of disease uncertainty of family members. The total Cronbach's α coefficient of the C-MUIS-FM was 0.887 in this study.

4.3.4 Social support rating scale (SSRS)

The SSRS was revised by Shuiyuan Xiao [18], which was used to assess current level of social support. The scale consisted of 3 dimensions, including subjective social support, objective social support, and social support use. Items 1-4 and 8-10 were single-choice questions, which was rated with 4-Point Likert Scale from 1 point to 4 points. Item 5 had 5 sub-items (A, B, C, D, F) and each sub-item was rated from 1 point (none) to 4 points (full support).For items 6 and 7, if "no source" were selected, it would be rated with 0 point; otherwise, each source selected under "from the following sources" would add 1 point to the total scores. The total scores of SSRS ranged from 12 to 66 points, with higher scores indicating higher levels of social support. The total Cronbach's alpha coefficient of SSRS was 0.788 in this study.

4.3.5 Chinese version of Life orientation test-Revised (LOT-R)

This Chinese version of LOT-R was translated by Wengen Deng [19] from English version of LOT-R [20]. The LOT-R was mainly used to assess the disposition of optimism or pessimism. The LOT-R was a single-dimensional self-assessment scale consisting of 10 items, with 3 items measuring optimism, 3 items measuring pessimism, and 4 items serving as fillers. Each item was rated with 5-Point Likert Scale ranging from 0 (strongly disagree) to 4 (strongly agree), and items 2, 4, and 5 were reverse scored. The total scores of the LOT-R ranged from 0 to 24 points, the higher scores, and the higher level of optimism. In this study, the total Cronbach's α coefficient was 0.831.

4.4 Statistical Analysis

The Statistical Package for the Social Sciences (SPSS) software version 26.0 was used for data input and statistical analyses. The socio-demographic and the characteristics of cancer of their care recipients cancer characteristics of SDMs were described by frequency and percentage. The scores of questionnaires were described by mean ± standard deviation or median (Interquartile Rangequartile) for normal or non-normal

distribution data. Univariate analyses were used to explore the difference of ACP engagement of SDMs in different categories of socio-demographic characteristics. Independent samples t~~T-~~-test or ANOVA test and Mann-Whitney U rank-sum test or Kruskal-Wallis H rank-sum test were used for normal or non-normal distribution data, respectively. Pearson or Spearman correlation analyses were used to explore the correlations of other variables, including the uncertainty of disease, social support and ~~optimism~~life orientation of participants, with ACP engagement of SDMs. Factors significant previously in univariate or correlation analyses were included in the multiple linear regression analyses with P >0.05 as the removal standard. If we encounter problems such as missing data that affect data integrity during the data analysis process, we will delete the participant's data.

4.5 Ethical considerations

This study was approved by Zhejiang Chinese Medical University Ethics Committee on 16th February 2023 (20230216-5). All participants were voluntary to anonymously participate in this study. Full explanation of this study was provided to all participants, and written informed consent was obtained before the interviews.

5 Results

During this study, of the 285 questionnaires administered, 9 responses were excluded because of missing data or illegible. Thus, 276 responses were finally analyzed (effective response rate 96.0%).

5.1 Socio-demographic characteristics of the participants

The participants' socio-demographic and cancer characteristics were presented in Table 1. The participants were evenly distributed by age and ~~gender~~sex. Most of the participants were married and lived with their family members. While, more than two-third~~2/3~~ (70.3% and 68.5%) of the participants had no experiences of medical decision-making or lacked knowledge of ACP. Most of the SDMs were the children (44.9%) or spouses (43.8%) of advanced cancer patients. Nearly two-third~~2/3~~ (63.4%) of the patients could take care of themselves in daily life.

Table 1 Socio-demographic characteristics of the participants (N=276)

Socio-demographic characteristics		Frequency (n)	Percentage (%)
**Age (years)**	18-40~~≤40~~	98	35.5
	41-60	113	40.9
	≥61	65	23.6
**~~Gender~~Sex**	Female	144	52.2
	Male	132	47.8
**Education status**	Primary school or less	46	16.7
	Junior high school	72	26.1
	Senior high school	57	20.7
	College	90	32.6
	Master degree or above~~Master or greater~~	11	4.0
**Marital status**	Unmarried or widowed or divorced	37	13.4
	Married	239	86.6
**Experiences of making medical decision**	No	194	70.3
	Yes	82	29.7
**Knowledge of ACP**	Unknown	189	68.5
	Heard of ACP	55	19.9
	Partly known	28	10.1
	Known	4	1.4
**Treatment expenditure**	≤ ¥10,000 (USD$1370)	110	39.9
	≤ ¥30,000 (USD$4110)	120	43.5
	≤ ¥50,000 (USD$6850)	28	10.1
	>¥50,000 (USD$6850)	18	6.5
**Annual income**	≤ ¥10,000 (USD$1370)	112	40.6
	≤ ¥30,000 (USD$4110)	101	36.6
	≤ ¥50,000 (USD$6850)	30	10.9
	>¥50,000 (USD$6850)	33	12.0
**~~Numbers of Family members~~The number**	≤3	110	39.9
	4-5	121	43.8

**of family members of SDMs**			
	≥6	45	16.3
**Relationship with the advanced cancer patientType of surrogate relationship**	Children	124	44.9
	Parents	19	6.9
	Spouse	121	43.8
	Siblings	6	2.2
	Grandchildren	6	2.2
**Classification of patients' cancer**	Digestive system tumors	112	40.6
	Hematologic cancers	34	12.3
	Genitourinary cancer	28	10.1
	Head and neck cancer	25	9.1
	Thoracic cancer	77	27.9
**Patient's ADL* independence levelPatients' independence level**	Dependent	24	8.7
	Partly dependent in daily life	77	27.9
	Independent in Daily life	62	22.5
	Completely dependent	113	40.9

* ADL: Activities of daily living

Formatted: Left

5.2 Factors influencing the ACP engagement of SDMs Factors associated with the ACP engagement of SDMs

Base on univariate analyses, the factors significant influencing the ACP engagement of SDMs were age, education level, experiences of making medical decision, knowledge of ACP, treatment expenditure, annual income, type of surrogate relationship. While, there was no significant difference in ACP engagement of SDMs stratified by marital status, numbers of family members of SDMs, classification of patients' cancer and patient's independence level (Table 2). In addition, the correlation between the uncertainty of disease, social support, optimismlife orientation of participants with ACP engagement of SDMs were shown in Table 3. The ACP engagement of SDMs were

negatively associated with uncertainty of disease (P<0.01), and positively associated with social support and ~~optimism~~life orientation of participants (both P<0.01).

Table 2 The difference of ACP engagement of SDMs with different socio-demographic and care recipients ~~cancer~~ characteristics

Characteristics	Subscales			Total [mean±SD)/ median(IQR)]
	Serving as SDM [mean±SD)/ median(IQR)]	Contemplation [mean±SD)/ median(IQR)]	Readiness [mean±SD)/ median(IQR)]	
**Age (years)**				
18-40~~≤40~~	25.10±3.63	11.00 (7.00)	19.00 (7.00)	55.50 (18.00)
41-60	23.38±5.25	10.00 (7.00)	17.00 (7.00)	49.00 (21.00)
≥61	21.46±5.76	7.00 (8.00)	14.00 (7.00)	43.00 (24.00)
F/H	10.996	5.605	16.536	16.604
P	**<0.001**	0.061	**<0.001**	**<0.001**
**~~Gender~~Sex**				
Female	22.51±4.93	9.00 (7.00)	16.00 (8.00)	47.50 (19.00)
Male	24.67±4.96	10.00 (8.00)	17.50 (9.00)	54.00 (22.00)
t/Z	-3.627	-0.557	-2.478	-2.678
P	**<0.001**	0.577	**0.013**	**0.007**
**Education level**				
Primary school or less	18.85±4.77	7.00 (2.00)	13.00 (3.00)	40.74±9.02
Junior high school	21.38±4.64	7.00 (4.00)	15.00 (5.00)	45.33±10.73
Senior high school	24.93±3.63	12.00 (6.00)	19.05±4.38	55.44±10.61
College	27.00 (5.00)	13.00 (7.00)	20.00 (8.00)	57.83±10.55

Formatted Table

Formatted: Font: Bold

Formatted: Font: Bold

Formatted: Font: Bold

Formatted: Font: Bold

Master degree or aboveMaster or higher	29.00 (2.00)	16.00 (5.00)	22.36±3.53	65.27±8.75
F/H	92.646	59.416	77.940	34.457
P	**<0.001**	**<0.001**	**<0.001**	**<0.001**
**Marital status**				
Unmarried or widowed or divorced	23.11±4.60	10.19±3.95	17.00 (9.00)	48.00 (22.00)
Married	24.00 (8.00)	10.00 (8.00)	17.00 (7.00)	49.00 (21.00)
Z	-0.831	-0.161	-0.284	-0.311
P	0.406	0.872	0.776	0.756
**Experiences of making medical decision**				
No	23.00 (6.00)	8.00 (6.00)	15.00 (6.00)	46.00 (15.00)
Yes	28.00 (4.00)	14.00 (5.00)	21.00±4.45	64.00 (12.00)
Z	-7.346	-6.780	-7.294	-7.852
P	**<0.001**	**<0.001**	**<0.001**	**<0.001**
**Knowledge of ACP**				
Unknown	21.90±4.78	8.00 (6.00)	15.90±4.13	46.00 (15.00)
Heard of ACP	28.00 (3.00)	14.00 (5.00)	21.25±3.94	64.00 (11.00)
Partly known	27.36±3.47	13.61±3.34	22.14±4.08	63.11±9.15
Known	26.50±4.93	10.75±4.27	17.50±5.80	54.75±14.18
F/H	70.245	59.818	36.639	82.403
P	**<0.001**	**<0.001**	**<0.001**	**<0.001**
**Treatment expenditure**				
≤¥10,000 (USD$1370)	22.40±4.52	7.00 (6.00)	15.00 (5.00)	43.00 (14.00)

Formatted: Font: Bold

Formatted: Font: Bold

Formatted: Font: Bold

≤¥30,000 (USD$4110)	25.00 (7.00)	11.50 (7.00)	18.50 (8.00)	53.72±12.98
≤¥50,000 (USD$6850)	25.50 (7.00)	12.00 (7.00)	19.18±4.91	54.36±11.71
>¥50,000 (USD$6850)	28.50 (5.00)	12.17±3.20	20.56±4.25	63.00 (12.00)
H	20.812	28.671	27.781	28.729
P	**<0.001**	**<0.001**	**<0.001**	**<0.001**
**Annual income**				
≤¥10,000 (USD$1370)	21.37±4.94	7.50（6.00）	14.50 (5.00)	43.50 (16.00)
≤¥30,000 (USD$4110)	24.00 (6.00)	11.00 (7.00)	18.00 (8.00)	52.00 (21.00)
≤¥50,000 (USD$6850)	27.50 (5.00)	12.50 (6.00)	18.40±4.60	55.70±12.45
>¥50,000 (USD$6850)	27.00 (3.00)	12.09±3.31	20.52±3.95	58.97±8.96
H	42.865	23.014	37.549	40.237
P	**<0.001**	**<0.001**	**<0.001**	**<0.001**
**~~Numbers of Family members~~The number of family members of SDMs**				
≤3	24.50 (8.00)	11.50 (8.00)	18.00 (9.00)	53.50 (24.00)
4-5	23.00 (7.00)	9.00 (7.00)	16.00 (7.00)	47.00 (19.00)
≥6	24.16±3.93	8.00 (8.00)	16.00 (7.00)	48.00 (20.00)
H	4.995	5.366	4.744	5.756
P	0.082	0.068	0.093	0.056
**Relationship with the advanced cancer patient~~Type of~~**				

Formatted: Font: Bold

Formatted: Font: Bold

~~surrogate relationship~~				
Children	24.69±4.15	11.00 (7.00)	18.00 (7.00)	54.00 (19.00)
Parents	21.37±4.11	7.74±2.88	13.00 (4.00)	44.21±9.38
Spouse	22.55±5.77	9.00 (8.00)	15.00 (8.00)	47.00 (24.00)
Siblings	26.17±2.93	10.50±4.46	18.83±4.79	55.50±11.36
Grandchildren	23.83±5.19	16.00 (8.00)	19.33±4.80	56.50±12.36
F/H	4.253	11.811	16.576	15.189
P	**0.002**	**0.019**	**0.002**	**0.004**
**Classification of patients' cancer**				
Digestive system tumors	23.00 (8.00)	10.00 (8.00)	17.00 (8.00)	51.38±12.55
Hematologic cancers	24.53±3.59	9.49±3.78	16.00 (7.00)	49.00 (19.00)
Genitourinary cancer	23.64±4.65	10.50 (9.00)	17.00 (11.00)	52.00 (27.00)
Head and neck cancer	23.64±5.83	8.00 (9.00)	16.36±4.89	46.00 (27.00)
Thoracic cancer	24.00 (9.00)	10.00 (7.00)	17.00 (9.00)	51.00 (24.00)
H	1.371	2.139	2.301	0.697
P	0.849	0.710	0.681	0.952
**Patients' ADL independence level** ~~**Patients' independence level**~~				
Dependent	25.50 (14.00)	12.50 (10.00)	17.46±5.70	51.25±16.50

Formatted: Font: Bold

Partly dependent in daily life	26.00 (9.00)	12.00 (8.00)	20.00 (10.00)	60.00 (23.00)
Independent in Daily life	23.42±4.74	10.00 (5.00)	16.00 (8.00)	48.00 (19.00)
Completely dependent	23.00 (6.00)	8.00 (7.00)	16.00 (6.00)	47.00 (15.00)
H	4.265	8.211	9.602	7.006
P	0.234	**0.042**	**0.022**	0.072

ADL: Activities of daily living; F: F value of ANOVA test, H: H value of Kruskal-Wallis H rank-sum test; t: t value of independent samples t t-test; Z: Z value of Mann-Whitney U rank-sum test; *: Serving as SDM, P-values indicating significance at the 0.05 level are shown in bold. contemplation, and readiness represent the three subscales of the ACP-17-SDM; significant at 0.05 significance level. .

Table 3 The correlation between the uncertainty of disease, social support, optimismlife orientation of participants with ACP engagement of SDMs –

**Characteristics**	**Subscales**			**Total** **Attitudes**
	**Serving as SDM**	**Contemplation**	**Readiness**	
Uncertainty of disease	-0.485**	-0.452**	-0.469**	-0.524**
Social support	0.514**	0.410**	0.501**	0.534**
Life orientation Optimism	0.356**	0.387**	0.401**	0.424**

**: P<0.01

5.3 –Multiple linear regression analysis for factors associated with the ACP engagement of SDMs

Factors influencing ACP engagement of SDMs –Factors associated with the ACP engagement of SDMs (Base on multiple linear regression analysis) –

Eight factors in univariate analysies and three factors in correlation analyses with P<0.05 were taken into multiple linear regression analysis. The stepwise regression method was performed to access the relationship between different variables. All independent variables were shown in Table 4, and the tolerance (TOL) of each variable in the model was greater than 0.1, and the variance iInflation factor (VIF) was less than

Formatted: Font: Bold

Formatted: Font: Bold

Formatted: Font: Bold

Formatted: Font: Bold

Formatted: Font: Bold

5. Thus, it was considered that there was no obvious multicollinearity between each variable.

Table 4 Independent variables assignment in multiple linear regression analysis

**Independent variable**	**Assignment**
Age	1=18-40≤40, 2=40-60, 3=≥61
GenderSex	1=Female, 2=Male
Education level	1= Primary school or less, 2= Junior high school, 3= Senior high school, 4= College, 5= Master degree or aboveMaster degree or higher
Experiences of medical decision-making	1=No, 2=Yes
Knowledge of ACP	1= Unknown, 2= Heard of ACP, 3= Partly known, 4= Known
Treatment expenditure	1=⩽ ¥10,000 (USD$1370), 2=⩽ ¥30,000 (USD$4110), 3=≤ ¥50,000 (USD$6850), 4=>¥50,000 (USD$6850)
Annual income	1=⩽ ¥10,000 (USD$1370), 2=⩽ ¥30,000 (USD$4110), 3=≤ ¥50,000 (USD$6850), 4=>¥50,000 (USD$6850)
Relationship with the advanced cancer patientType of surrogate relationship	1= Children, 2= Parents, 3= Spouse, 4= Siblings, 5= Grandchildren
The uncertainty of disease	null
The social support	null
The optimismlife orientation	null

Finally, as shown in Table 5, only seven variables revealed statistically significant influences on ACP engagement of SDMs and those variables included in the multiple linear regression model could explain 56.7% of the total variation (Adjusted R^2=0.567; F=52.448; P＜0.001). Those variables, except uncertainty of disease, presented a strong positive association with ACP engagement of SDMs.

Table 5 Multiple linear regression analysis of independent variables in ACP engagement of SDMs

Variables	Regression coefficient	Standardized coefficient	t	P	TOL	VIF
~~Constant~~	~~29.912~~	~~-~~	~~7.040~~	~~< 0.001~~		
Experiences of medical decision-making	6.144	0.224	4.934	**<0.001**	0.771	1.297
Education level	2.460	0.230	4.650	**<0.001**	0.491	2.035
Knowledge of ACP	1.989	0.116	2.387	**0.018**	0.649	1.541
Treatment expenditure	1.541	0.105	2.554	**0.011**	0.908	1.101
Uncertainty of disease	-0.196	-0.232	-5.051	**<0.001**	0.782	1.279
Social support	0.249	0.159	3.186	**0.002**	0.666	1.501
~~Optimism~~Life orientation	0.274	0.105	2.279	**0.023**	0.773	1.294

TOL: tolerance; VIF: variance inflation factor; P-values indicating significance at the 0.05 level are shown in bold. ~~significant at 0.05 significance level.~~

Formatted: Font: Bold

Formatted: Font: Bold

Formatted: Font: Bold

Formatted: Font: Bold

Formatted: Font: Bold

Formatted: Font: Bold

Formatted: Font: Bold

6 Discussion

This study investigated the ACP engagement of SDMs of Chinese advanced cancer patients, which provided valuable insights for ACP adaptations in mainland China. To our knowledge, this study was the first large sample study focusing on the factors influencing ACP engagement of SDMs of different advanced cancer patients in mainland China. Currently, there were few studies related to ACP engagement of patients at the end life in mainland China due to the Chinese traditional culture which limited the spread and implement of ACP [15].

A total of 276 SDMs of advanced cancer patients were analyzed in this study, with balanced distribution of ~~gender~~sex (52.2% female and 47.8% male). Most of participants were the children or spouse of the advanced cancer patients. Among the

participants, SDMs younger than 60 years old were account for a large proportion of total participants (76.4%), which were similar to the results of Wang's study [15]. Combined with the surrogate relationship with patients, we considered the following possibilities of this phenomenon. On one hand, most of advanced cancer patient were the elderly, and their children became the breadwinner of family which means their children would also take the role of SDMs. On the other hand, for the some middle-aged cancer patients, their spouse might become the first choice of surrogate decision makerSDM because their children were too young and their parents were too old to make decision. In our study, -participants with 18 to 40 years old have the highest level of ACP engagement among the different age groups and The univariate analyses showed that the mean ACP engagement score of the age group 18-40 is the highest among all. The univariate analyses showed that older SDMs had higher ACP engagement. tThis is different similar to findings by Liu which showed that older SDMs had higher ACP engagement [21]. This difference might be attributed the different population characteristics of care recipients, and Liu's study recruited community-dwelling elderly patients with chronic diseases. With age, the patients were more at risk of various diseases and they would think more about medical care and end-of-life issues. SDMs aged 18-40 have a stronger awareness of patient autonomy and rights. They are more likely to respect the patient's wishes and advocate for their active involvement in decision-making. Additionally, they may have better access to and understanding of medical information, enabling them to support the patient more effectively in navigating complex healthcare decisions.

Formatted: Font: 12 pt

Formatted: Font: 12 pt

Formatted: Font: 12 pt

Experiences of medical decision-making was significant associatedcorrelated with the engagement of ACP, and participants with experiences showed more positive engagement of ACP. However, only 29.7% of the participants had experiences of medical decision-making in this study, which might attribute to the limited implementation of the doctor-patient-surrogate decision-making model in mainland China. And it would hinder SDMs from fully obtaining the patient's medical information [22-24]. A recent study presented that SDMs with experiences of decision-making would be more deliberate when making medical decisions due to the knowledge of disease trajectory [25].

Relevant research showed that education level has an impact on the cognition, the higher education level, the better individual's cognitive ability [26]. Our study showed

that 36.6% of the participants' education level were greater than college, and 31.4% of them have partial knowledge of ACP, which showed a potential correlation between education level and cognition. The univariate analyses showed that the higher the education level was positively correlation with of SDMs, the ACP engagementstronger their willingness to participate in patient ACP discussions. And the resultsBesides wa, as similar to the study of Keam et al [2726]-, which also showed that participants with higher education level have had a more positive ACP engagement than those with lower education level. Participants with high education level tended to have a strong sense of autonomy, and consider the value of life and future medical intention when actively seeking disease information [2827, 2928]. Conversely, participants with low education level might lack knowledge to support their thinking, although they want to express their wishes [3029]. Participants had knowledge of ACP presented a more positive attitude than others, which is consistent with the study of Park et al [3130]. A full understanding of new things is the prerequisite for participation. Therefore, it is important for government, media and hospitals to establish theoretical frameworks to disseminate accurate information about ACP and strengthen the public's knowledge of ACP [3231]. To enhance the engagement participationof SDMs with restrictedlow education in ACP discussions for patients, it is essential to employ plain and straightforward languagecrucial to use simple and clear language to elucidateexplain the importance of ACP. and Pprovidinge them with easily digestible written resourceseasy to understand written materials and visual aids can also be beneficial. MoreoverAdditionally, offering training programssessions and securing support from healthcare professionals can also help SDMsthem better understand the patient's requirementsneeds and preferences, thus increasing their active participationinvolvement in ACP discussions. The different types of cancer were

This study showed that the treatment expenditure played an important role in making medical decisions by SDMs for advanced cancer patients, and the high cost of treatment expenditure often puts SDMs under great economic pressure [3332, 3433],. which in turn rendered them more predisposed to contemplate less invasive medical care approaches.made them more inclined to consider less aggressive medical care measures This financial burden further motivated economic pressure led to a higher willingness to participate in ACP discussions. The composition treatment expenditure was similar to annual income, which indicated the treatment expenditure of advanced cancer would

occupy the most of annual income, bringing huge economic burden to their families [3534-3736]. It is recommended that medical staff should comprehensively consider the treatment needs and economic status of patients and their family, assisting them to weigh the pros and cons of different treatment options and provide personalized medical decision-making recommendations [3837]. Additionally, policymakers shouldcould consider– implementing measures to alleviatereduce the financial burden of cancer treatment, such as expandingimproving insurance coverage and establishingproviding financial assistance programs, to ensure that patients have equal access to appropriate care and the patients and their SDMs are not unduly influenced by economic factors in ACP discussions.

The results in Table 3 and Table 5 both show that, there is a negative correlation between the scores of SDMs MUIS-FM and ACP-17-SDM, a positive correlation between the scores of SDMs SSRS and ACP-17-SDM, and a positive correlation between the scores of SDMs LOT-R and ACP-17-SDM. It meansTthis study showed that the engagement of ACP was negatively correlated to stronger the SDMs' sense of disease uncertainty, and positively correlated to the –weaker their willingness to participate in the patient's ACP. The stronger the SDMs' social support and life orientation ability. The uncertainty of disease was negatively correlated with ACP engagement. FirstiyFirstly, aAccording to the uncertainty in illness theory, uncertainty arises when individuals are unable to establish an adaptive cognitive framework for related events [3938]. Doctors and healthcare professionals play a vital role in facilitating communication between the patients and SDMs by offering support and guidance throughout these stages, especially for SMDsSDMs who have no medical knowledge. Secondly, sSocial support, referring to the material and spiritual support that individuals obtain from family or other social networks [39, 40, 41]., was positively associated with engagement of ACP. However, the results was inconsistent to a study investigating the factor influencing ACP engagement of SDMs of patients with hematologic malignancies [15], which might attributing the difference due to the characteristics of participants. However, there is also a study with similar conclusions to this one: participants with high levels of social support showed more active involvement in ACP and higher quality ACP completionParticipants with high scores of social support showed more active involvement of ACP and high-quality of ACP completion [4241]. Thirdly, mMany studies have shown that individuals under good

emotional state will result in more altruistic behaviors [~~43~~42-~~45~~44]. Optimistic SDMs often ~~would present positive attitudes to patients and~~ actively seek help from others to find solutions when encountering difficulties. Therefore, SDMs with strong life orientation ability ~~the optimistic SDMs~~ are more willing to participate in the discussion of patients' treatment decisions and have a more positive attitude towards ACP.

Patients play a central role in the development and implementation of the entire ACP, and it is crucial to facilitate communication between patients and SDMs. Healthcare providers should play an active role in facilitating communication between patients and SDMs. This includes providing clear and comprehensive information about ACP, its benefits, and the importance of the patient's active participation. Educational sessions and materials can help both patients and SDMs understand the process and their respective roles. Establishing a trusting relationship between patients, SDMs, and healthcare providers is essential. This can be achieved through open and empathetic communication, where patients feel comfortable expressing their wishes and concerns. Each patient and family is unique, and the approach to ACP should be tailored to their specific needs and circumstances. Healthcare providers should be sensitive to cultural, religious, and personal beliefs and preferences, and adapt their communication and support accordingly.

Our study highlights the importance of SDMs in ACP for advanced cancer patients. Future practice should focus on providing healthcare providers with enhanced training on how to effectively communicate with SDMs and patients about ACP. This includes training on cultural sensitivity, communication techniques, and strategies to overcome barriers to ACP discussions. ACP should be integrated into routine care for advanced cancer patients. Healthcare providers should make it a standard practice to discuss ACP with patients and their SDMs early in the disease trajectory. This can help ensure that patients' preferences and values are respected and that their care aligns with their wishes. In addition, future research should focus on longitudinal studies to better understand the long - term impact of ACP on patient outcomes and family satisfaction. Meanwhile, there is a need for more intervention studies to test the effectiveness of different ACP strategies.

Several limitations of this study should be acknowledged. Firstly, recruiting a convenience sample from a single cancer center may limit generalizability to national

target populations. Secondly, ~~Our analysis was constrained by the binary sex categorization available in the original dataset. Future research should adopt more inclusive gender identity measures that recognize non-binary and gender-diverse populations, better reflecting the social construct of gender.~~ ~~the individual perspectives of participants were not included in this study, such as the outlooks of world, life and value, which limited the and comprehensiveness. Further validation studies with a large national-wide sample and more comprehensive factors are need to warrant the conclusions.~~ the individual perspectives of participants, such as their worldviews, life outlooks, and values, were not included in this study, which limited the comprehensiveness of our conclusions. Further studies with a large, national-wide sample and more comprehensive factors are needed to substantiate our findings.

7 Conclusion

~~In mainland China, some interventions should be implemented to strengthen the willingness of the SDMs to participate in ACP. One of most important measure is to strengthen the dissemination of ACP concepts and avoid the public's misunderstanding. In addition, legal support is also essential for the progress of ACP, and clearly defined legislation can provide strong support for all participants involved the decision-making processes, making ACP practice more authoritative and actionable. Based on this study, we have identified some factors influencing the ACP engagement of SMDs, which might provide basic information for the construction of the educational scheme of ACP in mainland China and help to improve SDMs engagement in clinical ACP practice in future.~~This cross-sectional study ~~comprehensively explored the factors influencing the participation of surrogate decision-makers for advanced cancer patients in ACP. Our findings~~ demonstrated~~revealed~~ that multiple factors, including experiences of medical decision-making, education level, knowledge of ACP, treatment expenditure, uncertainty of disease, social support and life orientation, significantly impact the engagement of SDMs in ACP. In conclusion, this study provides valuable insights into the factors influencing ACP participation among surrogate decision-makers for advanced cancer patients. Future research should further explore the specific mechanisms underlying these factors and develop targeted interventions to improve ACP engagement, ultimately enhancing the quality of end-of-life care for patients with advanced cancer. ~~This research might provide basic information for the construction of~~

the educational scheme of ACP in mainland China and help to improve SDMs engagement in clinical ACP practice in future.

Author Contributions

Conceptualization: Xiaoqin Ma.

Data curation: Zhihao Han.

Formal analysis: Zhihao Han.

Supervision: Xiaoqin Ma.

Investigation: Guiyue Ma, Linxia Tang, Yong Fang.

Validation: Jingjing Su.

Writing– original draft: Zhihao Han, Guiyue Ma.

Writing– review & editing: Jingjing Su, Xiaoqin Ma.

Conflict Statement

No conflict of interest has been declared by the authors.

Funding Information

This project did not receive any grant from funding agencies in the public, commercial, or not-for-profit sectors.

Acknowledgments

This research was not funded by any public, commercial, or nonprofit agency. No competing financial interests exist. The authors thank all members of the cancer center for their help in this study.

Data Availability Statement

Data available on request from the authors.

REFERENCES

1. Rietjens JAC, Sudore RL, Connolly M, van Delden JJ, Drickamer MA, Droger M, et al. Definition and recommendations for advance care planning: an international consensus supported by the European Association for Palliative Care. The Lancet

Oncology. 2017; 18(9):e543-~~ee5~~51. https://doi.org/10.1016/s1470-2045(17)30582-x~~.~~ ~~PMID~~ PMID: 28884703.

2. Ribeiro C, Pamplona P, Simonds AK. Advance care planning in patients with respiratory failure. European respiratory review~~ -~~: an official journal of the European Respiratory Society. 2024; 33(174). https://doi.org/10.1183/16000617.0120-2024~~.~~ ~~PMID~~ PMID: 39537243.

3. Porcar Rodado E, Peral Sanchez D, Gisbert Grifo M. Advance Directives. Comparison of current legislation within the European Union. Spanish Journal of Legal Medicine. 2021; 47(2):66-73. ~~https://doi.org/~~https://doi.org/10.1016/j.remle.2020.05.011.

4. Bray F, Laversanne M, Sung H, Ferlay J, Siegel RL, Soerjomataram I, et al. Global cancer statistics 2022: GLOBOCAN estimates of incidence and mortality worldwide for 36 cancers in 185 countries. CA: a cancer journal for clinicians. 2024; 74(3):229-~~2~~63. https://doi.org/10.3322/caac.21834~~. PMID~~ PMID: 38572751.

5. Canny A, Mason B, Boyd K. Public perceptions of advance care planning (ACP) from an international perspective: a scoping review. BMC palliative care. 2023; 22(1):107. https://doi.org/10.1186/s12904-023-01230-4~~. PMID~~ PMID: 37507777.

6. Lu Y, Gu Y, Yu W. Hospice and Palliative Care in China: Development and Challenges. Asia-Pacific journal of oncology nursing. 2018; 5(1):26-32. https://doi.org/10.4103/apjon.apjon_72_17~~. PMID~~ PMID: 29379830.

7. Barry HC. Advance Care Planning Increases Execution of Advance Directives and Surrogate Decision-Maker Assignment. American family physician. 2018; 98(11):682-683~~. PMID~~ PMID: 30485037.

8. Ni P, Zhou J, Wang ZX, Nie R, Phillips J, Mao J. Advance directive and end-of-life care preferences among nursing home residents in Wuhan, China: a cross-sectional study. Journal of the American Medical Directors Association. 2014; 15(10):751-~~7~~56. https://doi.org/10.1016/j.jamda.2014.06.006~~. PMID~~ PMID: 25066002.

9. Bravo G, Dubois MF, Wagneur B. Assessing the effectiveness of interventions to promote advance directives among older adults: a systematic review and multi-level analysis. Social science & medicine (1982). 2008; 67(7):1122-~~11~~32. https://doi.org/10.1016/j.socscimed.2008.06.006~~. PMID~~ PMID: 18644667.

10. Tamayo-Velázquez MI, Simón-Lorda P, Villegas-Portero R, Higueras-Callejón C, García-Gutiérrez JF, Martínez-Pecino F, et al. Interventions to promote the use of advance directives: an overview of systematic reviews. Patient education and counseling. 2010; 80(1):10-20. https://doi.org/10.1016/j.pec.2009.09.027~~. PMID~~ PMID: 19879090.

11. Sudore RL, Heyland DK, Barnes DE, Howard M, Fassbender K, Robinson CA, et al. Measuring Advance Care Planning: Optimizing the Advance Care Planning Engagement Survey. Journal of pain and symptom management. 2017; 53(4):669-~~6~~81.e668. https://doi.org/10.1016/j.jpainsymman.2016.10.367~~. PMID~~ PMID: 28042072.

12. Sudore RL, Stewart AL, Knight SJ, McMahan RD, Feuz M, Miao Y, et al. Development and validation of a questionnaire to detect behavior change in multiple advance care planning behaviors. PLOS One. 2013; 8(9):e72465. https://doi.org/10.1371/journal.pone.0072465~~. PMID~~ PMID: 24039772.

13. Ni P, Chen JL, Liu N. Sample size estimation in quantitative research in nursing, Chinese Journal of Nursing, 45(2010) 378-~~3~~80.

14. Bandura A. Social cognitive theory: an agentic perspective. Annu Rev Psychol. 2001; 52:1-26. https://doi.org/10.1146/annurev.psych.52.1.1~~. PMID~~ PMID: 11148297.

15. Wang TH, Wang W, Shen WT, Shi BX, Participation in advance care planning and associated factors among surrogate decision makers of patients with hematologic m~~w~~alignancies, Chinese general practice, 25 (2022) 859-~~8~~66.

16. Van Scoy LJ, Day AG, Howard M, Sudore R, Heyland DK. Adaptation and Preliminary Validation of the Advance Care Planning Engagement Survey for Surrogate Decision Makers. Journal of pain and symptom management. 2019; 57(5):980-~~9~~88.e989. https://doi.org/10.1016/j.jpainsymman.2019.01.008~~. PMID~~ PMID: 30684633.

17. Wang WY, Wang S, Xia DS, Cui GP, Wang CM, Study on reliability and validity of Chinese version of Mishel uncertainty in illness scale family member form, Chinese nursing research, 26 (2012) 662-~~6~~63.

18. Xiao SY, Theory and research applications of the Social Support Rating Scale, Journal of clinical psychiatry, (1994) 98-100.

19. Deng WG, Wang P, Liu SS, Examining the issues and solutions for testing the dimensions of LOT and LOT-R using traditional methods, Journal of Shandong Normal University (Natural Science Edition), 24 (2009) 51-54. https://doi.org/10.3969/j.issn.1001-4748.2009.04.015.

20. Scheier MF, Carver CS, Bridges MW. Distinguishing optimism from neuroticism (and trait anxiety, self-mastery, and self-esteem): a reevaluation of the Life Orientation Test. Journal of personality and social psychology. 1994; 67(6):1063-~~10~~78. https://doi.org/10.1037//0022-3514.67.6.1063~~. PMID~~ PMID: 7815302.

21. Liu X, Chen H, Zhang L, Zhang Q, Feng T, Liu D. Advance Care Planning Engagement Among Family Members of Community-Dwelling Elderly Patients With Chronic Diseases in China: A Mixed-Methods Study. Journal of hospice and palliative nursing : JHPN : the official journal of the Hospice and Palliative Nurses Association. 2022; 24(2):e26-e34. https://doi.org/10.1097/njh.0000000000000829~~. PMID~~ PMID: 35045050.

22. Chen C, Cheng G, Chen X, Yu L. Information disclosure to cancer patients in Mainland China: A meta-analysis. Psycho-oncology. 2023; 32(3):342-~~3~~55. https://doi.org/10.1002/pon.6085~~. PMID~~ PMID: 36582008.

23. Hahne J, Liang T, Khoshnood K, Wang X, Li X. Breaking bad news about cancer in China: Concerns and conflicts faced by doctors deciding whether to inform patients. Patient education and counseling. 2020; 103(2):286-~~2~~91. https://doi.org/10.1016/j.pec.2019.08.022~~. PMID~~ PMID: 31455567.

24. Xu H, Yuan M. Family roles in informed consent from the perspective of young Chinese doctors: a questionnaire study. BMC medical ethics. 2024; 25(1):2. https://doi.org/10.1186/s12910-023-00999-6~~. PMID~~ PMID: 38172849.

25. Bakke BM, Feuz MA, McMahan RD, Barnes DE, Li B, Volow AM, et al. Surrogate Decision Makers Need Better Preparation for Their Role: Advice from Experienced Surrogates. Journal of palliative medicine. 2022; 25(6):857-~~8~~63. https://doi.org/10.1089/jpm.2021.0283~~. PMID~~ PMID: 35076297.

~~26. Lövdén M, Fratiglioni L, Glymour MM, Lindenberger U, Tucker-Drob EM. Education and Cognitive Functioning Across the Life Span. Psychological science in the public interest : a journal of the American Psychological Society. 2020;21(1):6-41. https://doi.org/10.1177/1529100620920576. PMID: 32772803.~~

~~27~~26. Keam B, Yun YH, Heo DS, Park BW, Cho CH, Kim S, et al. The attitudes of Korean cancer patients, family caregivers, oncologists, and members of the general public

Formatted: Indent: Left: -0.01"

toward advance directives. Supportive care in cancer~~-~~: official journal of the Multinational Association of Supportive Care in Cancer. 2013; 21(5):1437-~~14~~44. https://doi.org/10.1007/s00520-012-1689-z~~. PMID~~ PMID: 23262809.

~~28~~27. Wu F, Zhuang Y, Chen X, Wen H, Tao W, Lao Y, et al. Decision-making among the substitute decision makers in intensive care units: An investigation of decision control preferences and decisional conflicts. Journal of advanced nursing. 2020; 76(9):2323-~~23~~35. https://doi.org/10.1111/jan.14451~~. PMID~~ PMID: 32538477.

~~29~~28. Hopp FP. Preferences for surrogate decision makers, informal communication, and advance directives among community-dwelling elders: results from a national study. The Gerontologist. 2000; 40(4):449-~~4~~57. https://doi.org/10.1093/geront/40.4.449~~. PMID~~ PMID: 10961034.

~~30~~29. Rwabihama JP, Belmin J, Rakotoarisoa R, Hagege M, Audureau E, Benzengli H, et al. Promoting patients' rights at the end of life in a geriatric setting in France: The healthcare professionals' level of knowledge about surrogate decision-makers and advance directives. Patient education and counseling. 2020; 103(7):1390-~~13~~98. https://doi.org/10.1016/j.pec.2020.01.020~~. PMID~~ PMID: 32070651.

~~31~~30. Park HY, Kim YA, Sim JA, Lee J, Ryu H, Lee JL, et al. Attitudes of the General Public, Cancer Patients, Family Caregivers, and Physicians Toward Advance Care Planning: A Nationwide Survey Before the Enforcement of the Life-Sustaining Treatment Decision-Making Act. Journal of pain and symptom management. 2019; 57(4):774-~~7~~82. https://doi.org/10.1016/j.jpainsymman.2018.12.332~~. PMID~~ PMID: 30593911.

~~32~~31. Lin Goh SS. Singapore Takes Six Steps Forward in 'The Quality of Death Index' Rankings. Asia-Pacific journal of oncology nursing. 2018; 5(1):21-25. https://doi.org/10.4103/apjon.apjon_66_17~~. PMID~~ PMID: 29379829.

~~33~~32. Jeong W, Kim S, Kim H, Park EC, Jang SI. Effects of life-sustaining treatment plans on healthcare expenditure and healthcare utilization. BMC health services research. 2023; 23(1):1236. https://doi.org/10.1186/s12913-023-10235-x~~. PMID~~ PMID: 37950202.

~~34~~33. Longo CJ, Fitch MI, Loree JM, Carlson LE, Turner D, Cheung WY, et al. Patient and family financial burden associated with cancer treatment in Canada: a national study. Supportive care in cancer~~-~~: official journal of the Multinational Association of Supportive Care in Cancer. 2021; 29(6):3377-~~33~~86. https://doi.org/10.1007/s00520-020-05907-x~~. PMID~~ PMID: 33403399.

~~35~~34. Xu J, Wang J, Liu R, Xing J, Su L, Yu F, et al. Mental health inpatient treatment expenditure trends in China, 2005-2012: evidence from Shandong. The journal of mental health policy and economics. 2014; 17(4):173-~~1~~82~~. PMID~~ PMID: 25599281.

~~36~~35. Yao A, Shen X, Chai J, Cheng J, Liu R, Feng R, et al. Characteristics and implications of insurance-reimbursed inpatient care for gastric and oesophageal cancers in Anhui, China. International health. 2021; 13(5):446-~~4~~55. https://doi.org/10.1093/inthealth/ihaa092~~. PMID~~ PMID: 33210133.

~~37~~36. Fu W, Shi J, Zhang X, Liu C, Sun C, Du Y, et al. Effects of cancer treatment on household impoverishment: a multicentre cross-sectional study in China. BMJ open. 2021; 11(6):e044322. https://doi.org/10.1136/bmjopen-2020-044322~~. PMID~~ PMID: 34193481.

~~38~~37. van den Heuvel L, Meinders MJ, Post B, Bloem BR, Stiggelbout AM. Personalizing decision-making for persons with Parkinson's disease: where do we stand and what to improve? Journal of neurology. 2022; 269(7):3569-~~35~~78. https://doi.org/10.1007/s00415-022-10969-4~~. PMID~~ PMID: 35084559.

~~39~~38. Mishel MH. Reconceptualization of the uncertainty in illness theory. Image--the journal of nursing scholarship. 1990; 22(4):256-~~2~~62. https://doi.org/10.1111/j.1547-5069.1990.tb00225.x~~. PMID~~ PMID: 2292449.

~~40~~39. Usta YY. Importance of social support in cancer patients. Asian Pacific journal of cancer prevention : APJCP. 2012; 13(8):3569-~~35~~72. https://doi.org/10.7314/apjcp.2012.13.8.3569~~. PMID~~ PMID: 23098436.

~~41~~40. Hupcey JE. Clarifying the social support theory-research linkage. Journal of advanced nursing. 1998; 27(6):1231-~~12~~41. https://doi.org/10.1046/j.1365-2648.1998.01231.x~~. PMID~~ PMID: 9663875.

~~42~~41. Arch JJ, Bright EE, Finkelstein LB, Fink RM, Mitchell JL, Andorsky DJ, et al. Anxiety and Depression in Metastatic Cancer: A Critical Review of Negative Impacts on Advance Care Planning and End-of-Life Decision Making With Practical Recommendations. JCO oncology practice. 2023; 19(12):1097-~~11~~08. https://doi.org/10.1200/op.23.00287~~. PMID~~ PMID: 37831973.

~~43~~42. Feng L, Zhang L, Zhong H. Perceived Parenting Styles and Mental Health: The Multiple Mediation Effect of Perfectionism and Altruistic Behavior. Psychology research and behavior management. 2021; 14:1157-~~11~~70. https://doi.org/10.2147/prbm.S318446~~. PMID~~ PMID: 34377038.

~~44~~43. Brethel-Haurwitz KM, Stoianova M, Marsh AA. Empathic emotion regulation in prosocial behaviour and altruism. Cognition & emotion. 2020; 34(8):1532-~~15~~48. https://doi.org/10.1080/02699931.2020.1783517~~. PMID~~ PMID: 32576078.

~~45~~44. Barasch A, Levine EE, Berman JZ, Small DA. Selfish or selfless? On the signal value of emotion in altruistic behavior. Journal of personality and social psychology. 2014; 107(3):393-413. https://doi.org/10.1037/a0037207~~. PMID~~ PMID: 25133723.

Response to Reviewers

PONE-D-24-35495R1

Factors influencing the participation of surrogate decision-makers for advanced cancer patients in advance care planning: A cross-sectional study

We are very grateful to the reviewers for their revised comments on the manuscript, which are very important for the quality of the manuscript. On behalf of all the contributing authors, I would like to express our sincere appreciations of your letter and reviewers' constructive comments concerning our article entitled "Factors influencing the participation of surrogate decision-makers for advanced cancer patients in advance care planning: A cross-sectional study". We revised the manuscript again based on the reviewers' comments, hoping to meet PLOS ONE's publication criteria.

Reviewer #1: The authors do not fully explore the principles behind advance care planning and substitute decision making. Their analysis largely ignores the fundamental issues of patient autonomy and of decision makers needing to implement the patient's wishes. The authors mention that these are Western concepts that are new to China, but they do not make pay substantial attention to that point. The authors' conclusions are sound, but the design of the study did not fully explore the points made in the conclusion. The research design was not the most appropriate design for this topic.

Response: Many thanks to the reviewers for their constructive comments. There have been comprehensive studies on the autonomy of cancer patients in participating in ACP, and the focus group of our study is the surrogate decision-makers of cancer patients. We only conducted a questionnaire survey on the willingness and influencing factors of surrogate decision-makers before formally participating in ACP. The surrogate decision-makers did not actually participate in ACP. Because currently ACP has not been fully implemented in mainland China, and no relevant laws have been promulgated, but our research conclusions can provide some suggestions for law makers. In addition, ACP began to rise in countries such as Europe and the United States, and mainland China should learn from it. Currently, mainland China has very limited policies and laws regarding ACP. Therefore, we are unable to provide a substantive explanation of the implementation of ACP in mainland China.

In response to the issue mentioned by the reviewer that the research design is not suitable for this topic, we would like to explain that conducting quantitative research is the most important part of our research topic. We are currently conducting qualitative research on patients' surrogate decision-makers, with the purpose of supplementing and explaining the quantitative research results. We hope that subsequent research results can also be submitted to the PLOS ONE and have opportunity to be published.

Reviewer #2: There are major areas that need revision to strengthen the manuscript for publication. These have been highlighted in the attached file.

1) Multiple grammatical errors, and flow of ideas, need to be reviewed and edited throughout the manuscript. Please have the manuscript reviewed for grammatical and sentence structure errors to bring more clarity to the content.

Response: We sincerely appreciate the reviewers' insightful comments and constructive suggestions, which have significantly improved the quality of our manuscript. We have polished the grammar and sentence structure of the manuscript and hope that the revised manuscript will meet the publication requirements in terms of grammar and sentence structure.

2) Need to check the facts again, for example, the implementation of AD has been guaranteed in UK etc. This is mentioned in the introduction. However, it is not guaranteed or legally binding there due to multifactorial reasons including legal and practical. Kindly review for each country listed and remove "etc." wherever possible in the manuscript.

Response: The authors are grateful for the valuable feedback provided by the reviewers, as it has greatly enhanced the clarity and rigor of this work. We misspelled the England into UK. We have checked and revised the issues raised by the reviewer.

3) Background:

- Last paragraph of this section talks a lack of existing research on ACP engagement by SDMs. Are you referring to the situation in China? Please specify and elaborate with current evidence to support the need for this study.
- Talk about relational aspect to caregiving in Asian cultures, and how does it impact involvement in ACP.

- It is unclear if authors are referring to the need for the SDMs to engage in ACP with their patients living with cancer or by themselves. Please clarify. ACP is not possible without the presence of the person in question.

Response: We thank the reviewers for their thoughtful critiques, which have guided us in refining both the methodology and presentation of results. We first explain the doubts raised by the reviewers. We then discussed the impact of Asian nursing cultural background on participation in ACP. In addition, we need to explain that ACP emphasizes the joint participation of patients and patient surrogate decision-makers. Therefore, the main purpose of our study is to understand the willingness and influencing factors of patients' surrogate decision-makers to participate in the formulation and implementation of patients' ACP, rather than to explore the surrogate decision-makers themselves' participation in ACP. There are many studies focus on ACP engagement, while the study about ACP engagement of SDMs in mainland China is less, and this is the first large sample study focusing on the factors influencing ACP engagement of SDMs of different advanced cancer patients. We hope that our explanation will provide reviewers with a clear understanding of the purpose of this study.

4) Study Design:

- Clearly identify the study design used in this section e.g., cross sectional study.

Response: The constructive criticisms from the reviewers have been instrumental in strengthening the arguments and addressing gaps in the original submission. We provide a clear explanation of study design types in this section.

5) Participants and selection criteria:

- Consider using the term, "participants" instead of subjects.
- Move participant related information in this section from the study design section.
- Clearly identify who recruited participants and how e.g., nurse or doctor in the department. What was the role of investigators in recruitment. Procedure of recruitment remains unclear.

- Was the survey electronic or paper-based.

Response: We deeply acknowledge the reviewers' expertise, and their detailed suggestions have led to a more robust and coherent analysis. We have replaced "subjects" with "participants" based on comments made by reviewers. We have moved participant related information in this section from the study design section. Our investigators are hospital nurses and the questionnaire is in the form of a paper questionnaire. The investigators were nurses in the department, and completed all the recruitment.

6) Measurements:

- Please cite the studies utilized for devising sociodemographic survey.

Response: We extend our gratitude to the reviewers for their meticulous evaluation. We have added relevant references where necessary in the manuscript.

7) Results:

- How was "gender" captured, did you only include female and male? These are biological constructs whereas, gender is a social construct. Please review and change accordingly.

Response: We sincerely appreciate the reviewer's insightful comment regarding the distinction between biological sex and gender as a social construct. In response to this concern, we have replaced the term "gender" with "sex" throughout the manuscript.

8) Discussion:

- Your point about treatment expenditure and ACP is not clear. What are the clear recommendations here? Do you think people with less might be coerced into ACP discussions?

Response: Thank you for your valuable feedback. We understand your concern about the clarity of the relationship between treatment expenditure and advance care planning (ACP) and the potential implications of economic pressure on decision - making.

In our study, we found that high treatment costs significantly increased the economic burden on families, which in turn made them more inclined to consider less aggressive

medical care measures. This economic pressure led to a higher willingness to participate in ACP discussions. It’s important to note that this does not necessarily mean that people with less economic means were coerced into ACP discussions. Rather, the financial strain influenced their preferences and considerations regarding end - of - life care options.

To address your concerns and provide clearer recommendations, we suggest that healthcare providers should be aware of the economic impact on patients and their families when discussing treatment options and ACP. They should offer support and guidance to help patients and their families make informed decisions that align with their values and financial situations. Additionally, policymakers could consider measures to reduce the financial burden of cancer treatment, such as improving insurance coverage and providing financial assistance programs, to ensure that patients have equal access to appropriate care and are not unduly influenced by economic factors in ACP discussions.

We have revised the manuscript to better clarify these points and provide more detailed recommendations. We hope this addresses your concerns and enhances the quality of our research.

- Throughout your discussion, you don’t talk about the central role of the patient in ACP. ACP can’t happen without the active involvement of the patient. What are your recommendations to bring patients and SDM together to involve in ACP.

Response: Thank you for your insightful comment. You are right that the patient’s central role in advance care planning (ACP) is crucial. In our study, we focused on the factors affecting the involvement of SDMs. However, we acknowledge that we did not sufficiently emphasize the patient’s active involvement in ACP. To address your concern and provide clear recommendations on how to bring patients and SDMs together in ACP, we suggest the following:

Patients play a central role in the development and implementation of the entire ACP, and it is crucial to facilitate communication between patients and SDMs. Healthcare providers should play an active role in facilitating communication between patients and SDMs. This includes providing clear and comprehensive information about ACP, its

benefits, and the importance of the patient's active participation. Educational sessions and materials can help both patients and SDMs understand the process and their respective roles. Establishing a trusting relationship between patients, SDMs, and healthcare providers is essential. This can be achieved through open and empathetic communication, where patients feel comfortable expressing their wishes and concerns. Each patient and family is unique, and the approach to ACP should be tailored to their specific needs and circumstances. Healthcare providers should be sensitive to cultural, religious, and personal beliefs and preferences, and adapt their communication and support accordingly.

We have revised our manuscript to better address the patient's central role in ACP and to provide more detailed recommendations on how to involve both patients and SDMs in the process. We hope these changes address your concerns and enhance the quality of our research.

- What is your opinion on goals of care discussion for patients who are not able to participate in ACP process?

Response: Thank you for raising this important issue. You are correct that for patients who are not able to participate in the ACP process, the goals of care discussion become even more critical. In our study, titled "Factors influencing the participation of surrogate decision - makers for advanced cancer patients in advance care planning: A cross - sectional study," we focused on the factors affecting the involvement of SDMs. However, we acknowledge that we did not sufficiently address the specific needs of patients who cannot participate in ACP.

We should enhance communication between patients and surrogate decision-makers and emphasize the guiding role that healthcare providers play in this. We have added relevant content in the discussion section. We hope these changes address your concerns and enhance the quality of our research.

- Future implications for practice and research need to be further elaborated.

Response: Thank you for your valuable feedback. We appreciate your suggestion that the future implications for practice and research need to be further elaborated. In response to this, we have revised our manuscript to include more detailed discussions on the future directions for both clinical practice and research.

Our study highlights the importance of SDMs in ACP for advanced cancer patients. Future practice should focus on providing healthcare providers with enhanced training on how to effectively communicate with SDMs and patients about ACP. This includes training on cultural sensitivity, communication techniques, and strategies to overcome barriers to ACP discussions. ACP should be integrated into routine care for advanced cancer patients. Healthcare providers should make it a standard practice to discuss ACP with patients and their SDMs early in the disease trajectory. This can help ensure that patients' preferences and values are respected and that their care aligns with their wishes. In addition, future research should focus on longitudinal studies to better understand the long - term impact of ACP on patient outcomes and family satisfaction. Meanwhile, there is a need for more intervention studies to test the effectiveness of different ACP strategies.

We have revised our manuscript to include these points in the discussion section, providing a more comprehensive outlook on the future implications of our study. We believe these additions will enhance the value of our research and provide a clearer direction for future practice and research.

9) Limitations:

- Please elaborate on your point about "individual perspectives of participants were not included."

Response: Thank you for your insightful comment. We apologize for not elaborating on the point about "individual perspectives of participants were not included" in the original manuscript. Here's a more detailed explanation:

In this study, we mainly focused on the objective data and collective patterns. The individual perspectives, such as the outlooks of world, life and value, were indeed not taken into account. This is because the research design was primarily aimed at exploring the general trends and relationships among the variables at a macro level. The inclusion of individual perspectives would have required a more in-depth and qualitative approach, which was beyond the scope of the current research. However, we fully acknowledge that this limitation has affected the comprehensiveness of the study. We will make up for this shortcoming in subsequent research. We have also suggested that future research could adopt a mixed - methods approach to incorporate both the

objective data and the individual perspectives, so as to provide a more comprehensive understanding of the issue.

Once again, we appreciate your valuable suggestion and we have made the corresponding revisions to improve the quality of the paper.

Reviewer #3: The first and most crucial comment is to send your manuscript to professional editing before getting published. There were many grammatical mistakes and inconsistent use of terms, such as SDMs vs SMDs, severely affect readers' understanding. More specific comments are as below.

1) Section 1: introduction

- The connection between ACP & AD was not highlighted, despite the two concepts were compared. Why promoting ACP is significant and how does it relate to the completion of AD? Please explain.

Response: Thank you for your insightful comment regarding the connection between Advance Care Planning (ACP) and Advance Directives (AD). We appreciate this opportunity to clarify and elaborate on the significance of promoting ACP and its relation to the completion of AD.

Advance Care Planning (ACP): ACP is a process that involves discussions between patients, surrogate decision-makers, and healthcare providers about the patient's values, preferences, and goals for future healthcare. It is a dynamic and ongoing process that helps ensure that the patient's wishes are respected and followed, especially when they are no longer able to make decisions for themselves.

Advance Directives (AD): AD are legal documents that outline a patient's preferences for medical care in situations where they may not be able to communicate their wishes. AD can include living wills and durable power of attorney for healthcare.

Integration of AD into ACP: AD can be an important component of the ACP process. While ACP is a broader process that includes discussions and planning, AD serves as a legal document that formalizes these preferences. By promoting ACP, we can ensure that patients' preferences are not only discussed but also documented in a legally binding manner.

We have revised our manuscript to better highlight the connection between ACP and AD. We believe these additions will enhance the clarity and value of our research.

2) Section 2: Background

- Consider using the term “individual” instead of “patient” to minimize stigmatization.

Response: Thank you for your insightful suggestion. We fully agree with your point about minimizing stigmatization. In response to your comment, we have carefully reviewed the manuscript and have made the necessary changes to replace “patient” with “individual” where appropriate. We believe this amendment will enhance the inclusivity and sensitivity of our work. Thank you again for your valuable guidance.

3) Section 3: Aim

- Suggest moving the last sentence of this paragraph to the background section. It highlights the significance of the study rather than aim of this study.

Response: Thank you for your suggestion. We have carefully considered your comment and have moved the last sentence of the paragraph to the background section as you recommended. This change has been made to ensure that the paragraph now clearly focuses on the aim of the study, while the significance of the study is appropriately highlighted in the background section. Thank you for your helpful guidance.

4) Section 4.1: Study design

- Please state that this is a cross-sectional survey.

Response: Thank you for your suggestion. We appreciate your attention to the details of our study design. You are absolutely right that this is a cross-sectional survey. We have added a statement to clarify this in the relevant section of the manuscript. We believe this addition will provide a clearer description of the study design for our readers.

- Please clarify the duration of data collection period. It is not necessary to mention the period of evaluation (i.e. from July 2023 to January 2024).

Response: Thank you for your suggestion. We have revised the manuscript to clarify the duration of the data collection period. We have removed the mention of the evaluation period (i.e., from July 2023 to January 2024) as you suggested. The revised sentence now reads: “This data collection study was conducted from February 2023 to July 2023 at First Affiliated Hospital of Zhejiang Chinese Medical University.” We believe this change will provide a clearer and more concise description of the study timeline. Thank you again for your valuable feedback.

- Please state the data collection approach. Whether participants were invited to complete a self-report questionnaire or assisted by research assistants.

Response: Thank you for your suggestion. We have revised the manuscript to clarify the data collection approach.

Participants were recruited using convenience sampling methods. Paper questionnaires were used for data collection during the research process. Participants were invited to take part in this survey under the guidance of well-trained investigators. The investigators were hospital nurses who were responsible for explaining the study to the SDMs, and responsible for recycling and checking the paper questionnaires. The investigators were available to assist participants during the questionnaire filling process if they needed it, but most participants completed the questionnaire independently.

We believe this clarification will provide a better understanding of the data collection process for our readers.

5) Section 4.2: Participant selection

- Please clarify how SDM is appointed in your study setting. The phrase “appointed according to relevant regulations” is ambiguous.

Response: Thank you for your suggestion. We understand that the phrase “appointed according to relevant regulations” is ambiguous. We have revised the manuscript to clarify how SDM is appointed in our study setting. The revised sentence now reads as follows:

blood, marital, parental relationship with the advanced tumor patient (such as, parents, spouse, children, sibling etc.) and being appointed as a SDM by the patient through a

formal written designation, in accordance with the ethical principles and clinical guidelines relevant to the protection of patients' rights and interests.

We believe this revision provides a clearer explanation of the appointment process for SDMs in our study.

6) Section 4.3: Measurements

- Please cite the references that support your choice of exploring factors under the three dimensions.

Response: Thank you for your suggestion. We have revised the manuscript to include the references that support our choice of exploring factors under the three dimensions of person, environment, and behavior based on the Triadic Reciprocal Determinism theory. Specifically, we have added the following references:

Bandura A. Social cognitive theory: an agentic perspective. Annu Rev Psychol. 2001; 52:1-26. https://doi.org/10.1146/annurev.psych.52.1.1. PMID: 11148297.

The reference provide a solid theoretical foundation for our decision to explore factors under the three dimensions of person, environment, and behavior in our study.

7) Section 4.3.3 to 4.4.5: Instruments

- The description of scoring methods was lengthy. Consider delete it as long as the total score is reported for analysis. Showing the direction of total score is adequate.

Response: Thank you for your suggestion. We have carefully considered your comment regarding the description of the scoring methods. We understand that the detailed description may have been lengthy. In response to your suggestion, we have revised the manuscript to delete the detailed scoring methods, as long as the total score is reported for analysis. We believe that showing the direction of the total score is adequate for the purpose of this study.

8) Section 4.4 Statistical analysis

- The term “cancer characteristics of SDMs” are ambiguous. The SDMs are not cancer patient. Do you mean “the characteristics of cancer of their care recipients”.

Response: Thank you for pointing out the ambiguity in the term “cancer characteristics of SDMs.” You are absolutely right that SDMs are not cancer patients themselves. We meant to refer to “the characteristics of cancer of their care recipients.” We have revised the manuscript to clarify this terminology. Thank you again for your valuable feedback.

- Please state clearly if an independent t-test or paired t-test was used.

Response: Many thanks to the reviewers for their suggestions. In the statistical analysis part of this study, the independent T-test was used, and the paired T-test was not used. We have made this clear in the manuscript.

- When stating the p-value. Please consider if a small letter “*p”* or a capital letter “P” should be used.

Response: Thank you for your attention to the details of our manuscript. You raised a good point regarding the use of "p" or "P" when stating the p-value. We have reviewed the manuscript and will ensure that the p-value is consistently presented with a capital letter "P" in accordance with standard statistical reporting conventions.

- Consider addressing how missing data was treated in this section.

Response: Thank you for your suggestion. We have revised the manuscript to address how missing data was treated. During the data collection process, investigators will check the paper questionnaires filled out by participants to ensure the integrity of the data. However, during the data entry process, we still found 9 paper questionnaires with missing data and difficult to identify data. We eventually deleted these questionnaires to ensure the integrity of all data. We believe that this approach is appropriate for our study. Thank you again for your valuable feedback.

9) Section 5.2: Factors influencing the ACP engagement of SDMs

- This section reported the results of univariate analyses. The heading should be “factors associated with the ACP engagement of SDMs”

Response: Thank you for your suggestion. We have revised the heading of this section to "Factors associated with the ACP engagement of SDMs" as you recommended. We believe this change will make the content of this section clearer and more consistent with the results of the univariate analyses.

- The section heading should not be the same for section 5.2 and 5.3

Response: The thoughtful remarks from the reviewers have not only improved the manuscript but also deepened our understanding of the topic:

What is presented in 5.2 is the result of univariate analysis; what is presented in 5.3 is the result of multiple linear regression analysis, that is, the variables with statistical significance in the univariate analysis are further analyzed. In order to avoid duplication of the titles of 5.2 and 5.3, we modify the title of 5.3 to "Factors associated with the ACP engagement of SDMs (Base on multiple linear regression analysis)."

10) Table 2:

- The heading of columns were not aligned with the heading of table. If subscale score is reported, please indicate these are the three subscale component of ACP engagement.

Response: We are indebted to the reviewers for their rigorous feedback. The second, third, and fourth columns of data in Table 2 represent the scores of the three dimensions of Serving as SDM, Contemplation, and Readiness in the ACP-17-SDM scale respectively. The fifth column represents the total score of the above three dimensions in a specific group of people. We hope that our explanation can make the reviewers understand the content in Table 2.

- In the column heading, please indicate "mean (SD)" was reported in the table.

Response: Thank you for your careful consideration of our manuscript and for pointing out the need to clarify the statistical measures used in the table.

We have conducted normality tests on all the data. Since some of the data were found to be non - normally distributed, we have used "mean (SD)" to describe the normally distributed data and "median (IQR)" for the non - normally distributed data in the table.

We have revised the column heading in the table to clearly indicate the statistical measures used. We believe this revision will make the data presentation more accurate and understandable.

- The choice of statistical tests for certain variables were queried. Please review the test being used for “marital status”, “experience of making medical decision”

Response: We have the following explanations for the questions raised by reviewers and editors:

We divided marital status into two groups to clarify the differences in Serving as SDM, Contemplation, Readiness, and Total between the groups. After consulting with statistical experts and reviewing relevant literature, we concluded that the Mann-Whitney U test (Z test) is a non-parametric method suitable for comparing two independent samples when the data do not follow a normal distribution. It assesses whether the two groups come from the same distribution by comparing the rank sums of the data. Therefore, it is appropriate for the analysis of the variable of marital status.

Similarly, the Mann-Whitney U test (Z test) is also applicable for the analysis of medical decision-making experience.

- Please state the level of significance at the bottom of the table.

Response: Thank you for your suggestion. We have made the following revision to our manuscript:

We have added the level of significance at the bottom of the table. The level of significance is set at 0.05, which is a commonly used threshold for determining statistical significance. This information is now clearly stated at the bottom of the table.

Thank you for your attention to this detail. We believe this revision will make the statistical results in our manuscript clearer to the readers.

11) Table 3:

- Please clarify whether the construct reported here should be named as "life orientation" or "optimism"? Please align with section 4.3.5.

Response: Thank you for your comment. We have carefully considered your suggestion and have made the following revision:

We have changed the construct from "optimism" to "life orientation" throughout the manuscript to align with section 4.3.5. At the same time, we have made changes to other descriptions related to “life orientation” in the manuscript. This change ensures consistency and accuracy in the terminology used in our study.

- What was the variable “attitudes” reported here? It was not described in section 4.3

Response: Thank you for your comment. We recognize that the description of “attitudes” here was inappropriate:

What we intended to convey is that “attitudes” represents the total score of the three dimensions of the ACP-17-SDM scale (serving as SDM, contemplation, and readiness), and we aimed to explore the correlation between the total score of the ACP-17-SDM scale and the three variables Uncertainty of disease Social support and Life orientatio. We have changed “attitudes” to “Total” to correspond to the contents of Table 2. We have revised the relevant content in the manuscript to clarify this point.

12) Section 5.3

- The section heading should not be the same as section 5.2.

Response: Both reviewers raised the same question, and we have made positive changes to this question:

What is presented in 5.2 is the result of univariate analysis; what is presented in 5.3 is the result of multiple linear regression analysis, that is, the variables with statistical significance in the univariate analysis are further analyzed. In order to avoid duplication of the titles of 5.2 and 5.3, we modify the title of 5.3 to “Factors associated with the ACP engagement of SDMs (Base on multiple linear regression analysis).” At the same time, combined with the comments of another reviewer, we revised the title of 5.2 to: Factors associated with the ACP engagement of SDMs.

- Please check the presentation format of table 5. The collinearity statistics, TOL and VIF should be included in the table. Values for the constant is not necessarily to be reported.

Response: Thank you for your comment. We have checked the presentation format of Table 5 and made the following revisions:

We have included the collinearity statistics, Tolerance (TOL) and Variance Inflation Factor (VIF), in Table 5. We have removed the values for the constant, as they are not necessary to be reported. We believe these changes will improve the clarity and completeness of the table.

- Please add the level of significance/ removal standard at the bottom of the table.

Response: Thank you for your comment. We have added the level of significance at the bottom of the table. The level of significance is set at 0.05. These values are now clearly stated at the bottom of the table.

13) Section 6: Discussion

- The conclusion “The univariate analyses showed that older SDMs had higher ACP engagement” was not supported by the statistics. From table 2, the mean ACP engagement score of the age group 18-40 is the highest among all. Please update your discussion.

Response: We sincerely appreciate the reviewer’s insightful comment regarding the conclusion about the relationship between age and ACP engagement. Upon re-examining Table 2, we realized that the mean ACP engagement score of the 18-40 age group was indeed the highest among all age groups, which contradicts the previous conclusion that "older SDMs had higher ACP engagement." We have updated the discussion section to reflect this finding and provide a more accurate interpretation of the data.

In the revised discussion, we have highlighted that the 18-40 age group showed the highest level of ACP engagement, and we have explored potential reasons for this observation. We have also removed the previous conclusion.

We apologize for the oversight and are grateful for the reviewer's attention to detail. We believe that the revised manuscript provides a clearer and more accurate representation of the study findings.

- The discussion between educational level, cognitive ability and knowledge of ACP was not logical. People having higher educational level doesn't mean they have higher cognitive ability, as they may suffer from cognitive problem such as memory problem. The discussion followed cannot address the need of the less educated group. Please revise the implication drawn from this finding.

Response: We sincerely appreciate the reviewer's valuable feedback regarding the discussion of the relationship between educational level, cognitive ability, and knowledge of advance care planning (ACP). Upon reflection, we recognize that the original discussion incorrectly assumed a direct correlation between higher educational level and higher cognitive ability, overlooking the possibility of cognitive issues such as memory problems. We have revised the discussion to address this oversight.

In the revised manuscript, we have deleted the contradictory content in the manuscript. We have also emphasized the need to consider the specific needs and challenges of individuals with lower educational levels, ensuring that our implications are more inclusive and comprehensive.

Thank you again for your insightful comments, which have helped us improve the manuscript significantly.

- The discussion made based on the result of table 3 was not logical. Please explain why people having higher treatment expenditure reported higher level of ACP engagement; and why people having higher level of uncertainty of disease reported lower level of ACP engagement. Followed by the discussion of their implications.

Response: We apologize for the lack of clarity in the original discussion and appreciate the reviewer's valuable feedback. We have revised the discussion to better explain the relationship between higher treatment expenditure and higher ACP engagement. Table 2 (univariate analyses) and table 5 (multiple linear regression analysis) both show that treatment expenditure is an influencing factor for SDMs to participate in patients' ACP. The results in Table 2 show that SDMs with higher treatment expenditures have higher

scores on the ACP-17-SDM scale, indicating that SDMs are more willing to participate in patients’ ACP, and the differences between different groups are statistically significant. Therefore, we believe that SDMs with higher treatment expenditures have greater willingness to participate in patient ACP. SDMs with higher treatment expenditure may be more likely to engage in ACP due to several factors. Firstly, they may have a greater awareness of the importance of planning for future healthcare decisions, as they have already invested significantly in their treatment. Secondly, higher treatment expenditure may also be associated with better access to healthcare information and resources, making these individuals more informed and willing to participate in ACP. They may have established a closer relationship with their healthcare providers during the treatment process, which makes them feel more comfortable and confident in initiating ACP discussions.

We have also revised the discussion to clarify why SDMs with higher levels of disease uncertainty reported lower levels of ACP engagement. The results of Table 3 (correlation analysis) and Table 5 (multiple linear regression analysis) both show that there is a negative correlation between SDMs’ disease uncertainty scores and ACP-17-SDM scores, that is, the stronger the disease uncertainty, the weaker the SDMs’ willingness to participate in patient ACP. This is because high uncertainty increases anxiety and fear, making it difficult for them to think about and plan for future care. They may be so focused on the unknown aspects of the illness, such as prognosis and treatment outcomes, that they find it challenging to engage in ACP discussions. Additionally, the lack of clear information about the disease and its progression may hinder their ability to make informed decisions and express their preferences, further reducing their willingness to participate in ACP.

We hope our response can answer the reviewer’s questions. We would like to thank you again for your questions. This is very important for improving the quality of our manuscript.

- The recommendations made (public education and legal support) were not drawn from the study findings. Please tailored your recommendation specifically to a specific population that need extra attention and support to enhance their ACP engagement. And suggest what components should be

included in the ACP intervention. Recommendations should be discussed in Section 6 instead of section 7.

Response: We sincerely appreciate the reviewer's valuable feedback. We have carefully considered the comments and made the following revisions to our manuscript:

We have made recommendations for specific groups of people who need support and attention and have included these in Part 6. In addition, we have modified the conclusion section to summarize the study and point out the significance of the study for clinical practice. At the same time, we also provide an outlook for the next step of research.

We hope these revisions address the reviewer's concerns and enhance the quality of our manuscript. Thank you again for your valuable feedback.

- Please clarify the meaning of "validation studies".

Response: Thank you for your comment. We apologize for any ambiguity in our original statement. By "validation studies", we refer to research efforts that aim to confirm the reliability and validity of our findings. In this context, it means conducting further studies with a larger, national - wide sample and incorporating more comprehensive factors to ensure that our conclusions are robust and generalizable.

Thank you for your attention to this matter. We have revised the sentence for clarity: "Finally, the individual perspectives of participants, such as their worldviews, life outlooks, and values, were not included in this study, which limited the comprehensiveness of our conclusions. Further studies with a large, national - wide sample and more comprehensive factors are needed to substantiate our findings."

We have made every effort to revise the manuscript. This revision note aims to address the questions raised by the reviewers. We would like to express our gratitude once again for the suggestions provided by the reviewers, which are crucial for improving the quality of the research.

Zhihao Han, Jingjing Su, Guiyue Ma, Yong Fang, Linxia Tang, Xiaoqin Ma

March 4, 2025
