# Supplementary material for: Factors influencing the participation of surrogate decision-makers for advanced cancer patients in advance care planning: A cross-sectional study
Source: PLoS One. 2025 Jun 16;20(6):e0325551. doi: 10.1371/journal.pone.0325551 (PMC12169540; doi:10.1371/journal.pone.0325551)
Supplement: Attachment — Submitted filename: ACP in cancer.docx [file pone.0325551.s002.docx]

Factors influencing the participation of surrogate decision-makers for advanced cancer patients in advance care planning: A cross-sectional study.

Thank you for allowing me to review this important manuscript.

General feedback:

Overall, very well written manuscript and a well thought study.

Recommend using tenses consistently. Please have the manuscript reviewed for grammatical and sentence structure errors to bring more clarity to the content.

Recommend changing the term, “propaganda” as it has a negative connotation.

Abstract: Concise and well written. However, please identify the age group of patients you are focusing on.

Introduction: The flow of ideas is clear leading to your research question.

Methods and results:

Very thorough and well presented.

Discussion:

Please expand on your point regarding optimistic surrogate decision makers. Are you suggesting that people should be optimistic so healthcare providers can better engage them in ACP discussions? I think this is too much to ask from them when they are already dealing with cancer diagnosis and treatments. What can be done from the perspectives of healthcare providers and researchers to increase the uptake of ACP?

Conclusion:

Make conclusion concise and move future suggestions in research and practice implications as well as limitations to the discussion.

I recommend changing, “cognitive error” to another better term to describe your point.
