# Supplementary material for: Factors influencing the participation of surrogate decision-makers for advanced cancer patients in advance care planning: A cross-sectional study
Source: PLoS One. 2025 Jun 16;20(6):e0325551. doi: 10.1371/journal.pone.0325551 (PMC12169540; doi:10.1371/journal.pone.0325551)
Supplement: Attachment — Submitted filename: Respones to Reviewers.docx [file pone.0325551.s008.docx]

**Response to Reviewers**

PONE-D-24-35495

Factors influencing the participation of surrogate decision-makers for advanced cancer patients in advance care planning: A cross-sectional study

On behalf of all the contributing authors, I would like to express our sincere appreciations of your letter and reviewers’ constructive comments concerning our article entitled “Factors influencing the participation of surrogate decision-makers for advanced cancer patients in advance care planning: A cross-sectional study”. These comments are all valuable and helpful for improving our article. According to the reviewers’ comments, we have made modifications to our manuscript.

**Reviewer #1**: The authors have clearly identified surrogate decision-making as an important subject especially for patients with advanced cancer and the need for education and policy development in China. However, there are three main reasons why this article is not suitable for publication in its present form:

1) The English language is currently not up to publication standard and requires considerable editing. Also, there are incorrect facts in the Introduction about advance care planning in different countries, for example, Australia does not have federal laws regulating advance care planning, this is a Sate responsibility.

**Response**: Thank you for your comments. We have polished and revised the incorrected part in this paper.

2) The research method does not address the key principle of surrogate decision-making, that is, what would the patient want, even though it is mentioned in the Introduction when defining Advance Care Planning, “… which aims to respect the patient’s values … “. There is no mention of this fundamental reason for surrogate decision making in the Aim or Methods. The authors note there are limitations with the research including no interviews, but do not address how the participants would know or understand the decisions the patient would want. This is the basic principle underpinning advance care planning and as the authors mention will require interviews to understand the complex issues involved.

**Response**: Thank you for your comments. We have added the reason for surrogate decision making in the “Introduction” part. This study was mainly focus on the factors influencing the engagement of ACP, and we also invited some surrogate decision-makers to interview about the complex issues, but this part was not presented in this study.

3) Although the authors have not clearly explained their quantitative methodology, it is unclear how the participants were approached to be involved with this research. Were they given the questionnaire to complete, or were they assisted by one of the researchers. (As a qualitative methods research fellow, I have not commented on the quantitative results).

**Response**: Thank you for reminding us to add the methods of this study. We have added the procedure of our study in the revised manuscript in the “Study design” part.

**Reviewer #2**: Title: Factors influencing the participation of surrogate decision-makers for advanced cancer patients in advance care planning: A cross-sectional study.

Thank you for allowing me to review this important manuscript.

General feedback: Overall, very well written manuscript and a well thought study.

Recommend using tenses consistently. Please have the manuscript reviewed for grammatical and sentence structure errors to bring more clarity to the content.

**Response**: Thank you for reminding us the tenses in the paper, grammatical and sentence structure errors; and we have revised the full paper.

Recommend changing the term, “propaganda” as it has a negative connotation.

**Response**: Thank you for your comments. We have changed the term, “propaganda” into “dissemination”.

Abstract: Concise and well written. However, please identify the age group of patients you are focusing on.

**Response**: We mainly focused on the surrogate decision-makers of advanced patients, and patients who were eligible at different age groups were all recruited in this study.

Introduction: The flow of ideas is clear leading to your research question.

Methods and results: Very thorough and well presented.

Discussion: Please expand on your point regarding optimistic surrogate decision makers. Are you suggesting that people should be optimistic so healthcare providers can better engage them in ACP discussions? I think this is too much to ask from them when they are already dealing with cancer diagnosis and treatments. What can be done from the perspectives of healthcare providers and researchers to increase the uptake of ACP?

**Response**: Thank you for your comments and sorry for the misunderstanding. As your opinions, we could not ask them to do too much because they have already suffered a lot. However, as the study shown, the optimistic participants presented more active engagement of ACP, and it might be helpful for healthy caregivers to evaluate the status of patient or surrogate decision-makers to avoid some conflicts during the ACP.

Conclusion: Make conclusion concise and move future suggestions in research and practice implications as well as limitations to the discussion. I recommend changing, “cognitive error” to another better term to describe your point.

**Response**: Thank you for your comments. We have change the “cognitive error” into “misunderstanding”.

**Reviewer** **#3**: Comments: Thank you for offering the opportunity to review the manuscript titled “Factors influencing the participation of surrogate decision-makers for advanced cancer patients in advance care planning: A cross-sectional study”.

I believe the dataset of this study have valuable and important figures that worth dissemination. However, the quality of the current manuscript is far below the standard ready to publish. Here is an overall comment to address:

1) Please justify why the chosen study population “surrogate decision-maker of patients with advanced cancer” is important to study in the Chinese context. There should be more elaboration about how the Chinese culture affecting medical decisions and the dynamic between family caregivers and doctors. This would highlight the significance of this study.

**Response**: Thank you for your comments. We have added the related information in the introduction part.

2) The statistical tests performed in inferential analyses were not clearly reported. It is unclear about how the missing data was treated, and the completion rate of each outcome measure. The result tables do not clearly describe if mean (SD) is reported or not.

**Response**: Thank you for your comments. We have revised the Statistical Analysis part. “The socio-demographic and cancer characteristics of SDMs were described by frequency and percentage. The scores of questionnaires were described by mean ± standard deviation or median (quartile) for normal or non-normal distribution data”.

3) The discussion is superficial to add new knowledge to the field of study. Consider comparing the results with the following papers:

- WANG, T., WANG, W., SHEN, W., & SHI, B. (2022). Participation in Advance Care Planning and Associated Factors among Surrogate Decision Makers of Patients with Hematologic Malignancies. Chinese General Practice, 25(07), 859.

- Liu, X., Chen, H., Zhang, L., Zhang, Q., Feng, T., & Liu, D. (2022). Advance care planning engagement among family members of community-dwelling elderly patients with chronic diseases in China: a mixed-methods study. Journal of Hospice & Palliative Nursing, 24(2), E26-E34.

**Response**: Thank you for your suggestions. We have added those two papers in the discussion part.

**Editor #1**: I believe these three subscales should be reported in terms of mean (SD). Please specify.

**Response**: Thank you for the editor's suggestions. When we conducted the normality test on the data, we found that not all of the data conform to a normal distribution. Therefore, we only used mean (SD) to represent the data that meet the normal distribution criteria, while for the data that do not conform to a normal distribution, we used the median and interquartile range for representation.

We tried our best to improve the manuscript which will not influence the content and framework of the manuscript. We appreciate for Editor/Reviewers’ warm work earnestly, and hope the correction will meet with approval. Once again, thank you very much for your comments and suggestions.

Zhihao Han, Jingjing Su, Guiyue Ma, Yong Fang, Linxia Tang, Xiaoqin Ma

December 31th, 2024
