# Supplementary material for: Factors influencing the participation of surrogate decision-makers for advanced cancer patients in advance care planning: A cross-sectional study
Source: PLoS One. 2025 Jun 16;20(6):e0325551. doi: 10.1371/journal.pone.0325551 (PMC12169540; doi:10.1371/journal.pone.0325551)
Supplement: Attachment — Submitted filename: Revised manuscript (clean copy)_reviewer comment.docx [file pone.0325551.s005.docx]

The first and most crucial comment is to send your manuscript to professional editing before getting published. There were many grammatical mistakes and inconsistent use of terms, such as SDMs vs SMDs, severely affect readers’ understanding. More specific comments are as below:

**Section 1: introduction**

- The connection between ACP & AD was not highlighted, despite the two concepts were compared. Why promoting ACP is significant and how does it relate to the completion of AD? Please explain.

**Section 2: Background**

- Consider using the term “individual” instead of “patient” to minimize stigmatization.

**Section 3: Aim**

- Suggest moving the last sentence of this paragraph to the background section. It highlights the significance of the study rather than aim of this study.

**Section 4.1: Study design**

- Please state that this is a cross-sectional survey.
- Please clarify the duration of data collection period. It is not necessary to mention the period of evaluation (i.e. from July 2023 to January 2024).
- Please state the data collection approach. Whether participants were invited to complete a self-report questionnaire or assisted by research assistants.

**Section 4.2: Participant selection**

- Please clarify how SDM is appointed in your study setting. The phrase “appointed according to relevant regulations” is ambiguous.

**Section 4.3: Measurements**

- Please cite the references that support your choice of exploring factors under the three dimensions.

**Section 4.3.3 to 4.4.5: Instruments**

- The description of scoring methods was lengthy. Consider delete it as long as the total score is reported for analysis. Showing the direction of total score is adequate.

**Section 4.4 Statistical analysis**

- The term “cancer characteristics of SDMs” are ambiguous. The SDMs are not cancer patient. Do you mean “the characteristics of cancer of their care recipients”.
- Please state clearly if an independent t-test or paired t-test was used.
- When stating the p-value. Please consider if a small letter “*p”* or a capital letter “P” should be used.
- Consider addressing how missing data was treated in this section.

**Section 5.2: Factors influencing the ACP engagement of SDMs**

- This section reported the results of univariate analyses. The heading should be “factors associated with the ACP engagement of SDMs”
- The section heading should not be the same for section 5.2 and 5.3

**Table 2:**

- The heading of columns were not aligned with the heading of table. If subscale score is reported, please indicate these are the three subscale component of ACP engagement.
- In the column heading, please indicate “mean (SD)” was reported in the table.
- The choice of statistical tests for certain variables were queried. Please review the test being used for “marital status”, “experience of making medical decision”
- Please state the level of significance at the bottom of the table.

**Table 3:**

- Please clarify whether the construct reported here should be named as "life orientation" or "optimism"? Please align with section 4.3.5.
- What was the variable “attitudes” reported here? It was not described in section 4.3

**Section 5.3**

- The section heading should not be the same as section 5.2.
- Please check the presentation format of table 5. The collinearity statistics, TOL and VIF should be included in the table. Values for the constant is not necessarily to be reported.
- Please add the level of significance/ removal standard at the bottom of the table

**Section 6: Discussion**

- The conclusion “The univariate analyses showed that older SDMs had higher ACP engagement” was not supported by the statistics. From table 2, the mean ACP engagement score of the age group 18-40 is the highest among all. Please update your discussion.
- The discussion between educational level, cognitive ability and knowledge of ACP was not logical. People having higher educational level doesn’t mean they have higher cognitive ability, as they may suffer from cognitive problem such as memory problem. The discussion followed cannot address the need of the less educated group. Please revise the implication drawn from this finding.
- The discussion made based on the result of table 3 was not logical. Please explain why people having higher treatment expenditure reported higher level of ACP engagement; and why people having higher level of uncertainty of disease reported lower level of ACP engagement. Followed by the discussion of their implications.
- The recommendations made (public education and legal support) were not drawn from the study findings. Please tailored your recommendation specifically to a specific population that need extra attention and support to enhance their ACP engagement. And suggest what components should be included in the ACP intervention. Recommendations should be discussed in Section 6 instead of section 7.
- Please clarify the meaning of “validation studies”.
