# Supplementary material for: Factors influencing the participation of surrogate decision-makers for advanced cancer patients in advance care planning: A cross-sectional study
Source: PLoS One. 2025 Jun 16;20(6):e0325551. doi: 10.1371/journal.pone.0325551 (PMC12169540; doi:10.1371/journal.pone.0325551)
Supplement: Attachment — Submitted filename: PLOS One Jan 2025.docx [file pone.0325551.s007.docx]

# **Review**

Title: Factors influencing the participation of surrogate decision-makers for advanced cancer patients in advance care planning: A cross-sectional study.

| **General Comments** | - Thank you for allowing me to review this important manuscript. - Multiple grammatical errors, and flow of ideas, need to be reviewed and edited throughout the manuscript. Please have the manuscript reviewed for grammatical and sentence structure errors to bring more clarity to the content. - Need to check the facts again, for example, the implementation of AD has been guaranteed in UK etc. This is mentioned in the introduction. However, it is not guaranteed or legally binding there due to multifactorial reasons including legal and practical. Kindly review for each country listed and remove “etc.” wherever possible in the manuscript. |
| --- | --- |
| **Major Comments** | Background:   - Last paragraph of this section talks a lack of existing research on ACP engagement by SDMs. Are you referring to the situation in China? Please specify and elaborate with current evidence to support the need for this study. - Talk about relational aspect to caregiving in Asian cultures, and how does it impact involvement in ACP - It is unclear if authors are referring to the need for the SDMs to engage in ACP with their patients living with cancer or by themselves. Please clarify. ACP is not possible without the presence of the person in question.   Methods  Study Design:   - Clearly identify the study design used in this section e.g., cross sectional study.   Participants and selection criteria:   - Consider using the term, “participants” instead of subjects. - Move participant related information in this section from the study design section. - Clearly identify who recruited participants and how e.g., nurse or doctor in the department. What was the role of investigators in recruitment. Procedure of recruitment remains unclear. - Was the survey electronic or paper-based.   Measurements:   - Please cite the studies utilized for devising sociodemographic survey..   Results:   - How was “gender” captured, did you only include female and male? These are biological constructs whereas, gender is a social construct. Please review and change accordingly.   Discussion:   - Your point about treatment expenditure and ACP is not clear. What are the clear recommendations here? Do you think people with less might be coerced into ACP discussions? - Throughout your discussion, you don’t talk about the central role of the patient in ACP. ACP cant happen without the active involvement of the patient. What are your recommendations to bring patients and SDM together to involve in ACP. - What is your opinion on goals of care discussion for patients who are not able to participate in ACP process? - Future implications for practice and research need to be further elaborated.   Limitations:   - Please elaborate on your point about “individual perspectives of participants were not included.” |
