# Supplementary material for: Factors influencing the participation of surrogate decision-makers for advanced cancer patients in advance care planning: A cross-sectional study
Source: PLoS One. 2025 Jun 16;20(6):e0325551. doi: 10.1371/journal.pone.0325551 (PMC12169540; doi:10.1371/journal.pone.0325551)
Supplement: Attachment — Submitted filename: Response to Reviewers.docx [file pone.0325551.s011.docx]

**Response to Reviewers**

PONE-D-24-35495R1

Factors influencing the participation of surrogate decision-makers for advanced cancer patients in advance care planning: A cross-sectional study

We are very grateful to the reviewers for their revised comments on the manuscript, which are very important for the quality of the manuscript. On behalf of all the contributing authors, I would like to express our sincere appreciations of your letter and reviewers’ constructive comments concerning our article entitled “Factors influencing the participation of surrogate decision-makers for advanced cancer patients in advance care planning: A cross-sectional study”. We revised the manuscript again based on the reviewers’ comments, hoping to meet PLOS ONE’s publication criteria.

**Reviewer #1**: The authors do not fully explore the principles behind advance care planning and substitute decision making. Their analysis largely ignores the fundamental issues of patient autonomy and of decision makers needing to implement the patient’s wishes. The authors mention that these are Western concepts that are new to China, but they do not make pay substantial attention to that point. The authors’ conclusions are sound, but the design of the study did not fully explore the points made in the conclusion. The research design was not the most appropriate design for this topic.

**Response**: Many thanks to the reviewers for their constructive comments. There have been comprehensive studies on the autonomy of cancer patients in participating in ACP, and the focus group of our study is the surrogate decision-makers of cancer patients. We only conducted a questionnaire survey on the willingness and influencing factors of surrogate decision-makers before formally participating in ACP. The surrogate decision-makers did not actually participate in ACP. Because currently ACP has not been fully implemented in mainland China, and no relevant laws have been promulgated, but our research conclusions can provide some suggestions for law makers. In addition, ACP began to rise in countries such as Europe and the United States, and mainland China should learn from it. Currently, mainland China has very limited policies and laws regarding ACP. Therefore, we are unable to provide a substantive explanation of the implementation of ACP in mainland China.

In response to the issue mentioned by the reviewer that the research design is not suitable for this topic, we would like to explain that conducting quantitative research is the most important part of our research topic. We are currently conducting qualitative research on patients’ surrogate decision-makers, with the purpose of supplementing and explaining the quantitative research results. We hope that subsequent research results can also be submitted to the PLOS ONE and have opportunity to be published.

**Reviewer #2:** There are major areas that need revision to strengthen the manuscript for publication. These have been highlighted in the attached file.

1) Multiple grammatical errors, and flow of ideas, need to be reviewed and edited throughout the manuscript. Please have the manuscript reviewed for grammatical and sentence structure errors to bring more clarity to the content.

**Response**: We sincerely appreciate the reviewers’ insightful comments and constructive suggestions, which have significantly improved the quality of our manuscript. We have polished the grammar and sentence structure of the manuscript and hope that the revised manuscript will meet the publication requirements in terms of grammar and sentence structure.

2) Need to check the facts again, for example, the implementation of AD has been guaranteed in UK etc. This is mentioned in the introduction. However, it is not guaranteed or legally binding there due to multifactorial reasons including legal and practical. Kindly review for each country listed and remove “etc.” wherever possible in the manuscript.

**Response**: The authors are grateful for the valuable feedback provided by the reviewers, as it has greatly enhanced the clarity and rigor of this work. We misspelled the England into UK. We have checked and revised the issues raised by the reviewer.

3) Background:

- Last paragraph of this section talks a lack of existing research on ACP engagement by SDMs. Are you referring to the situation in China? Please specify and elaborate with current evidence to support the need for this study.
- Talk about relational aspect to caregiving in Asian cultures, and how does it impact involvement in ACP.
- It is unclear if authors are referring to the need for the SDMs to engage in ACP with their patients living with cancer or by themselves. Please clarify. ACP is not possible without the presence of the person in question.

**Response:** We thank the reviewers for their thoughtful critiques, which have guided us in refining both the methodology and presentation of results. We first explain the doubts raised by the reviewers. We then discussed the impact of Asian nursing cultural background on participation in ACP. In addition, we need to explain that ACP emphasizes the joint participation of patients and patient surrogate decision-makers. Therefore, the main purpose of our study is to understand the willingness and influencing factors of patients’ surrogate decision-makers to participate in the formulation and implementation of patients’ ACP, rather than to explore the surrogate decision-makers themselves’ participation in ACP. There are many studies focus on ACP engagement, while the study about ACP engagement of SDMs in mainland China is less, and this is the first large sample study focusing on the factors influencing ACP engagement of SDMs of different advanced cancer patients. We hope that our explanation will provide reviewers with a clear understanding of the purpose of this study.

4) Study Design:

- Clearly identify the study design used in this section e.g., cross sectional study.

**Response**: The constructive criticisms from the reviewers have been instrumental in strengthening the arguments and addressing gaps in the original submission. We provide a clear explanation of study design types in this section.

5) Participants and selection criteria:

- Consider using the term, “participants” instead of subjects.
- Move participant related information in this section from the study design section.
- Clearly identify who recruited participants and how e.g., nurse or doctor in the department. What was the role of investigators in recruitment. Procedure of recruitment remains unclear.
- Was the survey electronic or paper-based.

**Response**: We deeply acknowledge the reviewers’ expertise, and their detailed suggestions have led to a more robust and coherent analysis. We have replaced “subjects” with “participants” based on comments made by reviewers. We have moved participant related information in this section from the study design section. Our investigators are hospital nurses and the questionnaire is in the form of a paper questionnaire. The investigators were nurses in the department, and completed all the recruitment.

6) Measurements:

- Please cite the studies utilized for devising sociodemographic survey.

**Response**: We extend our gratitude to the reviewers for their meticulous evaluation. We have added relevant references where necessary in the manuscript.

7) Results:

- How was “gender” captured, did you only include female and male? These are biological constructs whereas, gender is a social construct. Please review and change accordingly.

**Response**: We sincerely appreciate the reviewer’s insightful comment regarding the distinction between biological sex and gender as a social construct. In response to this concern, we have replaced the term “gender” with “sex” throughout the manuscript.

8) Discussion:

- Your point about treatment expenditure and ACP is not clear. What are the clear recommendations here? Do you think people with less might be coerced into ACP discussions?

**Response:** Thank you for your valuable feedback. We understand your concern about the clarity of the relationship between treatment expenditure and advance care planning (ACP) and the potential implications of economic pressure on decision - making.

In our study, we found that high treatment costs significantly increased the economic burden on families, which in turn made them more inclined to consider less aggressive medical care measures. This economic pressure led to a higher willingness to participate in ACP discussions. It’s important to note that this does not necessarily mean that people with less economic means were coerced into ACP discussions. Rather, the financial strain influenced their preferences and considerations regarding end - of - life care options.

To address your concerns and provide clearer recommendations, we suggest that healthcare providers should be aware of the economic impact on patients and their families when discussing treatment options and ACP. They should offer support and guidance to help patients and their families make informed decisions that align with their values and financial situations. Additionally, policymakers could consider measures to reduce the financial burden of cancer treatment, such as improving insurance coverage and providing financial assistance programs, to ensure that patients have equal access to appropriate care and are not unduly influenced by economic factors in ACP discussions.

We have revised the manuscript to better clarify these points and provide more detailed recommendations. We hope this addresses your concerns and enhances the quality of our research.

- Throughout your discussion, you don’t talk about the central role of the patient in ACP. ACP can’t happen without the active involvement of the patient. What are your recommendations to bring patients and SDM together to involve in ACP.

**Response:** Thank you for your insightful comment. You are right that the patient’s central role in advance care planning (ACP) is crucial. In our study, we focused on the factors affecting the involvement of SDMs. However, we acknowledge that we did not sufficiently emphasize the patient’s active involvement in ACP. To address your concern and provide clear recommendations on how to bring patients and SDMs together in ACP, we suggest the following:

Patients play a central role in the development and implementation of the entire ACP, and it is crucial to facilitate communication between patients and SDMs. Healthcare providers should play an active role in facilitating communication between patients and SDMs. This includes providing clear and comprehensive information about ACP, its benefits, and the importance of the patient’s active participation. Educational sessions and materials can help both patients and SDMs understand the process and their respective roles. Establishing a trusting relationship between patients, SDMs, and healthcare providers is essential. This can be achieved through open and empathetic communication, where patients feel comfortable expressing their wishes and concerns. Each patient and family is unique, and the approach to ACP should be tailored to their specific needs and circumstances. Healthcare providers should be sensitive to cultural, religious, and personal beliefs and preferences, and adapt their communication and support accordingly.

We have revised our manuscript to better address the patient’s central role in ACP and to provide more detailed recommendations on how to involve both patients and SDMs in the process. We hope these changes address your concerns and enhance the quality of our research.

- What is your opinion on goals of care discussion for patients who are not able to participate in ACP process?

**Response:** Thank you for raising this important issue. You are correct that for patients who are not able to participate in the ACP process, the goals of care discussion become even more critical. In our study, titled "Factors influencing the participation of surrogate decision - makers for advanced cancer patients in advance care planning: A cross - sectional study," we focused on the factors affecting the involvement of SDMs. However, we acknowledge that we did not sufficiently address the specific needs of patients who cannot participate in ACP.

We should enhance communication between patients and surrogate decision-makers and emphasize the guiding role that healthcare providers play in this. We have added relevant content in the discussion section. We hope these changes address your concerns and enhance the quality of our research.

- Future implications for practice and research need to be further elaborated.

**Response**: Thank you for your valuable feedback. We appreciate your suggestion that the future implications for practice and research need to be further elaborated. In response to this, we have revised our manuscript to include more detailed discussions on the future directions for both clinical practice and research.

Our study highlights the importance of SDMs in ACP for advanced cancer patients. Future practice should focus on providing healthcare providers with enhanced training on how to effectively communicate with SDMs and patients about ACP. This includes training on cultural sensitivity, communication techniques, and strategies to overcome barriers to ACP discussions. ACP should be integrated into routine care for advanced cancer patients. Healthcare providers should make it a standard practice to discuss ACP with patients and their SDMs early in the disease trajectory. This can help ensure that patients’ preferences and values are respected and that their care aligns with their wishes. In addition, future research should focus on longitudinal studies to better understand the long - term impact of ACP on patient outcomes and family satisfaction. Meanwhile, there is a need for more intervention studies to test the effectiveness of different ACP strategies.

We have revised our manuscript to include these points in the discussion section, providing a more comprehensive outlook on the future implications of our study. We believe these additions will enhance the value of our research and provide a clearer direction for future practice and research.

9) Limitations:

- Please elaborate on your point about “individual perspectives of participants were not included.”

**Response:** Thank you for your insightful comment. We apologize for not elaborating on the point about "individual perspectives of participants were not included" in the original manuscript. Here’s a more detailed explanation:

In this study, we mainly focused on the objective data and collective patterns. The individual perspectives, such as the outlooks of world, life and value, were indeed not taken into account. This is because the research design was primarily aimed at exploring the general trends and relationships among the variables at a macro level. The inclusion of individual perspectives would have required a more in-depth and qualitative approach, which was beyond the scope of the current research. However, we fully acknowledge that this limitation has affected the comprehensiveness of the study. We will make up for this shortcoming in subsequent research. We have also suggested that future research could adopt a mixed - methods approach to incorporate both the objective data and the individual perspectives, so as to provide a more comprehensive understanding of the issue.

Once again, we appreciate your valuable suggestion and we have made the corresponding revisions to improve the quality of the paper.

**Reviewer #3**: The first and most crucial comment is to send your manuscript to professional editing before getting published. There were many grammatical mistakes and inconsistent use of terms, such as SDMs vs SMDs, severely affect readers’ understanding. More specific comments are as below.

1) Section 1: introduction

- The connection between ACP & AD was not highlighted, despite the two concepts were compared. Why promoting ACP is significant and how does it relate to the completion of AD? Please explain.

**Response:** Thank you for your insightful comment regarding the connection between Advance Care Planning (ACP) and Advance Directives (AD). We appreciate this opportunity to clarify and elaborate on the significance of promoting ACP and its relation to the completion of AD.

Advance Care Planning (ACP): ACP is a process that involves discussions between patients, surrogate decision-makers, and healthcare providers about the patient’s values, preferences, and goals for future healthcare. It is a dynamic and ongoing process that helps ensure that the patient’s wishes are respected and followed, especially when they are no longer able to make decisions for themselves.

Advance Directives (AD): AD are legal documents that outline a patient’s preferences for medical care in situations where they may not be able to communicate their wishes. AD can include living wills and durable power of attorney for healthcare.

Integration of AD into ACP: AD can be an important component of the ACP process. While ACP is a broader process that includes discussions and planning, AD serves as a legal document that formalizes these preferences. By promoting ACP, we can ensure that patients’ preferences are not only discussed but also documented in a legally binding manner.

We have revised our manuscript to better highlight the connection between ACP and AD. We believe these additions will enhance the clarity and value of our research.

2) Section 2: Background

- Consider using the term “individual” instead of “patient” to minimize stigmatization.

**Response:** Thank you for your insightful suggestion. We fully agree with your point about minimizing stigmatization. In response to your comment, we have carefully reviewed the manuscript and have made the necessary changes to replace “patient” with “individual” where appropriate. We believe this amendment will enhance the inclusivity and sensitivity of our work. Thank you again for your valuable guidance.

3) Section 3: Aim

- Suggest moving the last sentence of this paragraph to the background section. It highlights the significance of the study rather than aim of this study.

**Response:** Thank you for your suggestion. We have carefully considered your comment and have moved the last sentence of the paragraph to the background section as you recommended. This change has been made to ensure that the paragraph now clearly focuses on the aim of the study, while the significance of the study is appropriately highlighted in the background section. Thank you for your helpful guidance.

4) Section 4.1: Study design

- Please state that this is a cross-sectional survey.

**Response:** Thank you for your suggestion. We appreciate your attention to the details of our study design. You are absolutely right that this is a cross-sectional survey. We have added a statement to clarify this in the relevant section of the manuscript. We believe this addition will provide a clearer description of the study design for our readers.

- Please clarify the duration of data collection period. It is not necessary to mention the period of evaluation (i.e. from July 2023 to January 2024).

**Response:** Thank you for your suggestion. We have revised the manuscript to clarify the duration of the data collection period. We have removed the mention of the evaluation period (i.e., from July 2023 to January 2024) as you suggested. The revised sentence now reads: “This data collection study was conducted from February 2023 to July 2023 at First Affiliated Hospital of Zhejiang Chinese Medical University.” We believe this change will provide a clearer and more concise description of the study timeline. Thank you again for your valuable feedback.

- Please state the data collection approach. Whether participants were invited to complete a self-report questionnaire or assisted by research assistants.

**Response:** Thank you for your suggestion. We have revised the manuscript to clarify the data collection approach.

Participants were recruited using convenience sampling methods. Paper questionnaires were used for data collection during the research process. Participants were invited to take part in this survey under the guidance of well-trained investigators. The investigators were hospital nurses who were responsible for explaining the study to the SDMs, and responsible for recycling and checking the paper questionnaires. The investigators were available to assist participants during the questionnaire filling process if they needed it, but most participants completed the questionnaire independently.

We believe this clarification will provide a better understanding of the data collection process for our readers.

5) Section 4.2: Participant selection

- Please clarify how SDM is appointed in your study setting. The phrase “appointed according to relevant regulations” is ambiguous.

**Response:** Thank you for your suggestion. We understand that the phrase “appointed according to relevant regulations” is ambiguous. We have revised the manuscript to clarify how SDM is appointed in our study setting. The revised sentence now reads as follows:

blood, marital, parental relationship with the advanced tumor patient (such as, parents, spouse, children, sibling etc.) and being appointed as a SDM by the patient through a formal written designation, in accordance with the ethical principles and clinical guidelines relevant to the protection of patients’ rights and interests.

We believe this revision provides a clearer explanation of the appointment process for SDMs in our study.

6) Section 4.3: Measurements

- Please cite the references that support your choice of exploring factors under the three dimensions.

**Response:** Thank you for your suggestion. We have revised the manuscript to include the references that support our choice of exploring factors under the three dimensions of person, environment, and behavior based on the Triadic Reciprocal Determinism theory. Specifically, we have added the following references:

Bandura A. Social cognitive theory: an agentic perspective. Annu Rev Psychol. 2001; 52:1-26. https://doi.org/10.1146/annurev.psych.52.1.1. PMID: 11148297.

The reference provide a solid theoretical foundation for our decision to explore factors under the three dimensions of person, environment, and behavior in our study.

7) Section 4.3.3 to 4.4.5: Instruments

- The description of scoring methods was lengthy. Consider delete it as long as the total score is reported for analysis. Showing the direction of total score is adequate.

**Response:** Thank you for your suggestion. We have carefully considered your comment regarding the description of the scoring methods. We understand that the detailed description may have been lengthy. In response to your suggestion, we have revised the manuscript to delete the detailed scoring methods, as long as the total score is reported for analysis. We believe that showing the direction of the total score is adequate for the purpose of this study.

8) Section 4.4 Statistical analysis

- The term “cancer characteristics of SDMs” are ambiguous. The SDMs are not cancer patient. Do you mean “the characteristics of cancer of their care recipients”.

**Response:** Thank you for pointing out the ambiguity in the term “cancer characteristics of SDMs.” You are absolutely right that SDMs are not cancer patients themselves. We meant to refer to “the characteristics of cancer of their care recipients.” We have revised the manuscript to clarify this terminology. Thank you again for your valuable feedback.

- Please state clearly if an independent t-test or paired t-test was used.

**Response:** Many thanks to the reviewers for their suggestions. In the statistical analysis part of this study, the independent T-test was used, and the paired T-test was not used. We have made this clear in the manuscript.

- When stating the p-value. Please consider if a small letter “*p”* or a capital letter “P” should be used.

**Response:** Thank you for your attention to the details of our manuscript. You raised a good point regarding the use of "p" or "P" when stating the p-value. We have reviewed the manuscript and will ensure that the p-value is consistently presented with a capital letter "P" in accordance with standard statistical reporting conventions.

- Consider addressing how missing data was treated in this section.

**Response:** Thank you for your suggestion. We have revised the manuscript to address how missing data was treated. During the data collection process, investigators will check the paper questionnaires filled out by participants to ensure the integrity of the data. However, during the data entry process, we still found 9 paper questionnaires with missing data and difficult to identify data. We eventually deleted these questionnaires to ensure the integrity of all data. We believe that this approach is appropriate for our study. Thank you again for your valuable feedback.

9) Section 5.2: Factors influencing the ACP engagement of SDMs

- This section reported the results of univariate analyses. The heading should be “factors associated with the ACP engagement of SDMs”

**Response:** Thank you for your suggestion. We have revised the heading of this section to “Factors associated with the ACP engagement of SDMs” as you recommended. We believe this change will make the content of this section clearer and more consistent with the results of the univariate analyses.

- The section heading should not be the same for section 5.2 and 5.3

**Response:** The thoughtful remarks from the reviewers have not only improved the manuscript but also deepened our understanding of the topic:

What is presented in 5.2 is the result of univariate analysis; what is presented in 5.3 is the result of multiple linear regression analysis, that is, the variables with statistical significance in the univariate analysis are further analyzed. In order to avoid duplication of the titles of 5.2 and 5.3, we modify the title of 5.3 to “Factors associated with the ACP engagement of SDMs (Base on multiple linear regression analysis).”

10) Table 2:

- The heading of columns were not aligned with the heading of table. If subscale score is reported, please indicate these are the three subscale component of ACP engagement.

**Response:** We are indebted to the reviewers for their rigorous feedback. The second, third, and fourth columns of data in Table 2 represent the scores of the three dimensions of Serving as SDM, Contemplation, and Readiness in the ACP-17-SDM scale respectively. The fifth column represents the total score of the above three dimensions in a specific group of people. We hope that our explanation can make the reviewers understand the content in Table 2.

- In the column heading, please indicate “mean (SD)” was reported in the table.

**Response:** Thank you for your careful consideration of our manuscript and for pointing out the need to clarify the statistical measures used in the table.

We have conducted normality tests on all the data. Since some of the data were found to be non - normally distributed, we have used “mean (SD)” to describe the normally distributed data and “median (IQR)” for the non - normally distributed data in the table.

We have revised the column heading in the table to clearly indicate the statistical measures used. We believe this revision will make the data presentation more accurate and understandable.

- The choice of statistical tests for certain variables were queried. Please review the test being used for “marital status”, “experience of making medical decision”

**Response:** We have the following explanations for the questions raised by reviewers and editors:

We divided marital status into two groups to clarify the differences in Serving as SDM, Contemplation, Readiness, and Total between the groups. After consulting with statistical experts and reviewing relevant literature, we concluded that the Mann-Whitney U test (Z test) is a non-parametric method suitable for comparing two independent samples when the data do not follow a normal distribution. It assesses whether the two groups come from the same distribution by comparing the rank sums of the data. Therefore, it is appropriate for the analysis of the variable of marital status.

Similarly, the Mann-Whitney U test (Z test) is also applicable for the analysis of medical decision-making experience.

- Please state the level of significance at the bottom of the table.

**Response:** Thank you for your suggestion. We have made the following revision to our manuscript:

We have added the level of significance at the bottom of the table. The level of significance is set at 0.05, which is a commonly used threshold for determining statistical significance. This information is now clearly stated at the bottom of the table.

Thank you for your attention to this detail. We believe this revision will make the statistical results in our manuscript clearer to the readers.

11) Table 3:

- Please clarify whether the construct reported here should be named as "life orientation" or "optimism"? Please align with section 4.3.5.

**Response:** Thank you for your comment. We have carefully considered your suggestion and have made the following revision:

We have changed the construct from "optimism" to "life orientation" throughout the manuscript to align with section 4.3.5. At the same time, we have made changes to other descriptions related to “life orientation” in the manuscript. This change ensures consistency and accuracy in the terminology used in our study.

- What was the variable “attitudes” reported here? It was not described in section 4.3

**Response:** Thank you for your comment. We recognize that the description of “attitudes” here was inappropriate:

What we intended to convey is that “attitudes” represents the total score of the three dimensions of the ACP-17-SDM scale (serving as SDM, contemplation, and readiness), and we aimed to explore the correlation between the total score of the ACP-17-SDM scale and the three variables Uncertainty of disease Social support and Life orientatio. We have changed “attitudes” to “Total” to correspond to the contents of Table 2. We have revised the relevant content in the manuscript to clarify this point.

12) Section 5.3

- The section heading should not be the same as section 5.2.

**Response:** Both reviewers raised the same question, and we have made positive changes to this question:

What is presented in 5.2 is the result of univariate analysis; what is presented in 5.3 is the result of multiple linear regression analysis, that is, the variables with statistical significance in the univariate analysis are further analyzed. In order to avoid duplication of the titles of 5.2 and 5.3, we modify the title of 5.3 to “Factors associated with the ACP engagement of SDMs (Base on multiple linear regression analysis).” At the same time, combined with the comments of another reviewer, we revised the title of 5.2 to: Factors associated with the ACP engagement of SDMs.

- Please check the presentation format of table 5. The collinearity statistics, TOL and VIF should be included in the table. Values for the constant is not necessarily to be reported.

**Response:** Thank you for your comment. We have checked the presentation format of Table 5 and made the following revisions:

We have included the collinearity statistics, Tolerance (TOL) and Variance Inflation Factor (VIF), in Table 5. We have removed the values for the constant, as they are not necessary to be reported. We believe these changes will improve the clarity and completeness of the table.

- Please add the level of significance/ removal standard at the bottom of the table.

**Response:** Thank you for your comment. We have added the level of significance at the bottom of the table. The level of significance is set at 0.05. These values are now clearly stated at the bottom of the table.

13) Section 6: Discussion

- The conclusion “The univariate analyses showed that older SDMs had higher ACP engagement” was not supported by the statistics. From table 2, the mean ACP engagement score of the age group 18-40 is the highest among all. Please update your discussion.

**Response:** We sincerely appreciate the reviewer’s insightful comment regarding the conclusion about the relationship between age and ACP engagement. Upon re-examining Table 2, we realized that the mean ACP engagement score of the 18-40 age group was indeed the highest among all age groups, which contradicts the previous conclusion that "older SDMs had higher ACP engagement." We have updated the discussion section to reflect this finding and provide a more accurate interpretation of the data.

In the revised discussion, we have highlighted that the 18-40 age group showed the highest level of ACP engagement, and we have explored potential reasons for this observation. We have also removed the previous conclusion.

We apologize for the oversight and are grateful for the reviewer’s attention to detail. We believe that the revised manuscript provides a clearer and more accurate representation of the study findings.

- The discussion between educational level, cognitive ability and knowledge of ACP was not logical. People having higher educational level doesn’t mean they have higher cognitive ability, as they may suffer from cognitive problem such as memory problem. The discussion followed cannot address the need of the less educated group. Please revise the implication drawn from this finding.

**Response:** We sincerely appreciate the reviewer’s valuable feedback regarding the discussion of the relationship between educational level, cognitive ability, and knowledge of advance care planning (ACP). Upon reflection, we recognize that the original discussion incorrectly assumed a direct correlation between higher educational level and higher cognitive ability, overlooking the possibility of cognitive issues such as memory problems. We have revised the discussion to address this oversight.

In the revised manuscript, we have deleted the contradictory content in the manuscript. We have also emphasized the need to consider the specific needs and challenges of individuals with lower educational levels, ensuring that our implications are more inclusive and comprehensive.

Thank you again for your insightful comments, which have helped us improve the manuscript significantly.

- The discussion made based on the result of table 3 was not logical. Please explain why people having higher treatment expenditure reported higher level of ACP engagement; and why people having higher level of uncertainty of disease reported lower level of ACP engagement. Followed by the discussion of their implications.

**Response:** We apologize for the lack of clarity in the original discussion and appreciate the reviewer’s valuable feedback. We have revised the discussion to better explain the relationship between higher treatment expenditure and higher ACP engagement. Table 2 (univariate analyses) and table 5 (multiple linear regression analysis) both show that treatment expenditure is an influencing factor for SDMs to participate in patients’ ACP. The results in Table 2 show that SDMs with higher treatment expenditures have higher scores on the ACP-17-SDM scale, indicating that SDMs are more willing to participate in patients’ ACP, and the differences between different groups are statistically significant. Therefore, we believe that SDMs with higher treatment expenditures have greater willingness to participate in patient ACP. SDMs with higher treatment expenditure may be more likely to engage in ACP due to several factors. Firstly, they may have a greater awareness of the importance of planning for future healthcare decisions, as they have already invested significantly in their treatment. Secondly, higher treatment expenditure may also be associated with better access to healthcare information and resources, making these individuals more informed and willing to participate in ACP. They may have established a closer relationship with their healthcare providers during the treatment process, which makes them feel more comfortable and confident in initiating ACP discussions.

We have also revised the discussion to clarify why SDMs with higher levels of disease uncertainty reported lower levels of ACP engagement. The results of Table 3 (correlation analysis) and Table 5 (multiple linear regression analysis) both show that there is a negative correlation between SDMs’ disease uncertainty scores and ACP-17-SDM scores, that is, the stronger the disease uncertainty, the weaker the SDMs’ willingness to participate in patient ACP. This is because high uncertainty increases anxiety and fear, making it difficult for them to think about and plan for future care. They may be so focused on the unknown aspects of the illness, such as prognosis and treatment outcomes, that they find it challenging to engage in ACP discussions. Additionally, the lack of clear information about the disease and its progression may hinder their ability to make informed decisions and express their preferences, further reducing their willingness to participate in ACP.

We hope our response can answer the reviewer’s questions. We would like to thank you again for your questions. This is very important for improving the quality of our manuscript.

- The recommendations made (public education and legal support) were not drawn from the study findings. Please tailored your recommendation specifically to a specific population that need extra attention and support to enhance their ACP engagement. And suggest what components should be included in the ACP intervention. Recommendations should be discussed in Section 6 instead of section 7.

**Response:** We sincerely appreciate the reviewer’s valuable feedback. We have carefully considered the comments and made the following revisions to our manuscript:

We have made recommendations for specific groups of people who need support and attention and have included these in Part 6. In addition, we have modified the conclusion section to summarize the study and point out the significance of the study for clinical practice. At the same time, we also provide an outlook for the next step of research.

We hope these revisions address the reviewer’s concerns and enhance the quality of our manuscript. Thank you again for your valuable feedback.

- Please clarify the meaning of “validation studies”.

**Response:** Thank you for your comment. We apologize for any ambiguity in our original statement. By “validation studies”, we refer to research efforts that aim to confirm the reliability and validity of our findings. In this context, it means conducting further studies with a larger, national - wide sample and incorporating more comprehensive factors to ensure that our conclusions are robust and generalizable.

Thank you for your attention to this matter. We have revised the sentence for clarity: “Finally, the individual perspectives of participants, such as their worldviews, life outlooks, and values, were not included in this study, which limited the comprehensiveness of our conclusions. Further studies with a large, national - wide sample and more comprehensive factors are needed to substantiate our findings.”

We have made every effort to revise the manuscript. This revision note aims to address the questions raised by the reviewers. We would like to express our gratitude once again for the suggestions provided by the reviewers, which are crucial for improving the quality of the research.

Zhihao Han, Jingjing Su, Guiyue Ma, Yong Fang, Linxia Tang, Xiaoqin Ma

March 4, 2025
