# Supplementary material for: Factors influencing the participation of surrogate decision-makers for advanced cancer patients in advance care planning: A cross-sectional study
Source: PLoS One. 2025 Jun 16;20(6):e0325551. doi: 10.1371/journal.pone.0325551 (PMC12169540; doi:10.1371/journal.pone.0325551)
Supplement: Attachment — Submitted filename: PLOS One Mar 2025.docx [file pone.0325551.s010.docx]

# **Review**

Title: Factors influencing the participation of surrogate decision-makers for advanced cancer patients in advance care planning: A cross-sectional study.

| **General Comments** | - Thank you for allowing me to review this manuscript for the third time. Most of the comments have been responded to however there are still some points that need addressing. - Multiple grammatical errors, and flow of ideas, need to be reviewed and edited throughout the manuscript. Please have the manuscript reviewed for grammatical and sentence structure errors to bring more clarity to the content. |
| --- | --- |
| **Major Comments** | Background:   - Last paragraph of this section talks a lack of existing research on ACP engagement by SDMs. But it is still unclear if authors are referring to the need for the SDMs to engage in ACP with or without their patients (family members). - It is unclear how will you use the findings of this study apart from education. Who will be the target for the education - You have mentioned education, but what is your opinion on reducing/addressing the hierarchy existing between elders and physicians. How will knowing SDMs’ perspective help with removing barriers related to hierarchy?   Discussion:   - Your rationalization on yours and Liu study differences are not very clear. - Its unclear what you mean by “new things” as a prerequisite for participation in ACP, please elaborate. - You talk about the role of government, media and hospital in establishing theoretical framework. I don’t believe that’s their role. But I am also wondering if you are referring to policies instead of theoretical framework. - Your point regarding higher financial burden leading to high participation in ACP by SDMs is contradictory to the actual philosophy of ACP, which must involve the patient. It appears you are referring to care decisions made by SDMs only. - You talk about patients playing a central role in developing ACP and suggest healthcare providers should be sensitive to culture, religion etc., these are generic suggestions. I am interested to know recommendations related to the Mainland China. |
