# Supplementary material for: Factors influencing the participation of surrogate decision-makers for advanced cancer patients in advance care planning: A cross-sectional study
Source: PLoS One. 2025 Jun 16;20(6):e0325551. doi: 10.1371/journal.pone.0325551 (PMC12169540; doi:10.1371/journal.pone.0325551)
Supplement: Attachment — Submitted filename: Response_to_Reviewers_auresp_3.docx [file pone.0325551.s013.docx]

**Response to Reviewers**

Factors influencing the participation of surrogate decision-makers for advanced cancer patients in advance care planning: A cross-sectional study

Thank you for providing us with this final opportunity to revise our manuscript and for your continued guidance throughout the review process. We sincerely appreciate the detailed feedback from the reviewers and the editorial team, and we have carefully addressed all concerns raised in the previous decision letter. Attached please find the revised manuscript with tracked changes, along with a point-by-point response to each comment. We hope these revisions now fully meet the journal’s requirements and look forward to your final evaluation.

**Reviewer**:

1) Multiple grammatical errors, and flow of ideas, need to be reviewed and edited throughout the manuscript. Please have the manuscript reviewed for grammatical and sentence structure errors to bring more clarity to the content.

**Response**: Regarding your first comment about grammatical errors and the flow of ideas, we have taken immediate action to address this issue.

We have engaged a professional editing service to thoroughly review and polish the manuscript, ensuring that all grammatical and sentence structure errors have been corrected. In fact, we previously engaged a language editing service to assist with manuscript revisions, and we have again requested their assistance to polish the language of the manuscript for this submission. This process has significantly improved the clarity and readability of the content, and we are confident that the manuscript now meets the journal's requirements for language quality.

Additionally, we have attached a certificate from the editing service as proof of the language polishing. Please find it in the attachment for your reference.

2) Background: Last paragraph of this section talks a lack of existing research on ACP engagement by SDMs. But it is still unclear if authors are referring to the need for the SDMs to engage in ACP with or without their patients (family members).

**Response**: Regarding your comment about the lack of existing research on ACP engagement by SDMs, we would like to clarify:

The manuscript emphasizes the insufficient research on SDMs engagement in ACP with patients, rather than SDMs engagement in ACP without patients. We have revised the relevant section to better reflect this focus and to avoid any potential misunderstanding.

We sincerely thank you for pointing out this issue, which has helped us improve the clarity and accuracy of our manuscript.

3) Background: It is unclear how will you use the findings of this study apart from education. Who will be the target for the education.

**Response**: Thank you for your insightful comments on our manuscript. We have carefully considered your feedback and made the following revisions:

①Clarified the application of the study findings beyond just education. We have specified that the educational scheme will target SDMs of advanced cancer patients and aim to enhance their understanding and participation in ACP.

②Specified the target audience for the education as SDMs of advanced cancer patients.

We believe these revisions address your concerns and strengthen the manuscript. We appreciate your time and effort in reviewing our work and providing valuable feedback.

4) Background: You have mentioned education, but what is your opinion on reducing/addressing the hierarchy existing between elders and physicians. How will knowing SDMs’ perspective help with removing barriers related to hierarchy?

**Response**: Thank you for your insightful comments on our manuscript. We have carefully considered your feedback and made the following revisions:

Addressed the issue of hierarchy by discussing how understanding SDMs' perspectives can help identify and reduce barriers related to the hierarchical respect for elders and physicians. We have emphasized the importance of promoting more egalitarian communication and decision-making processes through targeted education.

We believe these revisions address your concerns and strengthen the manuscript.

5) Discussion: Your rationalization on yours and Liu study differences are not very clear.

**Response**: Thank you for your insightful comments on our manuscript. We have carefully considered your feedback and made the following revisions to address the differences between our study and Liu's study:

①Clarified the specific characteristics of the care recipients in both studies, highlighting that Liu's study focused on elderly patients with chronic diseases, while our study centered on advanced cancer patients.

②Provided a more detailed explanation of why younger SDMs in our study exhibited higher ACP engagement, considering factors such as adaptability and access to resources.

We believe these revisions provide a clearer rationale for the differences observed in our study compared to Liu's study and strengthen the manuscript. We appreciate your time and effort in reviewing our work and providing valuable feedback.

6) Discussion: Its unclear what you mean by "new things" as a prerequisite for participation in ACP, please elaborate.

**Response**: Thank you for your insightful comments on our manuscript. We have carefully considered your feedback and made the following revisions:

We agree that the term "new things" required clarification. In the revised manuscript, we have replaced it with "relatively unfamiliar healthcare concepts like ACP" and added contextual explanation about cultural barriers to end-of-life discussions. This modification better specifies that ACP itself represents a novel decision-making paradigm in certain sociocultural settings.

We appreciate your feedback in strengthening the precision of this critical conceptual linkage.

7) Discussion: You talk about the role of government, media and hospital in establishing theoretical framework. I don’t believe that’s their role. But I am also wondering if you are referring to policies instead of theoretical framework.

**Response**: Thank you for your insightful comments on our manuscript. We have carefully considered your feedback.

We acknowledge that the original phrasing regarding "theoretical frameworks" was ambiguous and potentially misleading. As you rightly pointed out, these entities are better positioned to implement practical policies rather than abstract theoretical constructs. In the revised text, we have replaced "theoretical frameworks" with "policy-driven strategies" to better reflect the practical roles of governments (e.g., funding policies and regulatory support), media (e.g., public awareness initiatives), and hospitals (e.g., integrating ACP into clinical workflows).

We sincerely appreciate your expertise in identifying this inconsistency.

8) Discussion: Your point regarding higher financial burden leading to high participation in ACP by SDMs is contradictory to the actual philosophy of ACP, which must involve the patient. It appears you are referring to care decisions made by SDMs only.

**Response**: Thank you for your astute observation. We acknowledge that the original phrasing could inadvertently imply that SDMs’ financial motivations replace patient-centered ACP principles, which contradicts the core ethos of shared decision-making. In the revised text, we have:

①Clarified that SDMs’ cost-related decisions arise only when patient preferences are undocumented, emphasizing ACP’s foundational goal of proactively capturing patient autonomy.

②Reframed the financial burden as a catalyst for advocating early patient engagement in ACP, aligning with its philosophy of preemptive communication.

Your critique has strengthened the manuscript’s alignment with ACP’s ethical framework. We deeply appreciate your expertise.

9) Discussion: You talk about patients playing a central role in developing ACP and suggest healthcare providers should be sensitive to culture, religion etc., these are generic suggestions. I am interested to know recommendations related to the Mainland China.

**Response**: Thank you for emphasizing the need for region-specific insights. We have substantially revised this section to address Mainland China’s unique challenges and opportunities in ACP implementation:

①Added evidence on SDMs’ predominant role in family hierarchies, citing studies about filial piety’s dual impact on ACP engagement.

② Researchers could develop ACP decision aids tailored to Chinese culture. These tools should primarily focus on patient-related information, helping patients understand their disease characteristics and predict treatment outcomes. Currently, there are no researchers in Mainland China who have developed relevant decision aids.

③ Aligned hospital policies with the "Healthy China 2030" framework, emphasizing measurable outcomes (e.g., ACP documentation rates) to drive institutional change.

These additions, supported by region-specific references [47], transform generic recommendations into actionable strategies for the Chinese context. We deeply appreciate your guidance in enhancing the manuscript’s relevance.

Thank you once again for your patience and constructive feedback. We have dedicated significant effort to refining this manuscript in response to the reviewers’ and editors’ concerns. Should any further revisions be required, please do not hesitate to contact us. We sincerely hope that the current version now aligns with the journal’s standards and would be honoured to contribute to PLOS ONE’s scientific community.

Best regards,

Zhihao Han, Jingjing Su, Guiyue Ma, Yong Fang, Linxia Tang, Xiaoqin Ma

Zhejiang Chinese Medical University

[20041028@zcmu.edu.cn](mailto:20041028@zcmu.edu.cn)
