# Supplementary material for: Factors influencing the participation of surrogate decision-makers for advanced cancer patients in advance care planning: A cross-sectional study
Source: PLoS One. 2025 Jun 16;20(6):e0325551. doi: 10.1371/journal.pone.0325551 (PMC12169540; doi:10.1371/journal.pone.0325551)
Supplement: Attachment — Submitted filename: Response to Reviewer.docx [file pone.0325551.s014.docx]

**Response to Reviewers**

Factors influencing the participation of surrogate decision-makers for advanced cancer patients in advance care planning: A cross-sectional study

We sincerely appreciate the detailed feedback from the reviewers and the editorial team, and we have carefully addressed all concerns raised in the decision letter. Attached please find the revised manuscript with tracked changes, along with a point-by-point response to each comment. We hope these revisions now fully meet the journal’s requirements and look forward to your final evaluation.

**Reviewer**:

1) Background: In the 4th paragraph, I believe the meaning of “there are few studies focusing on the ACP engagement of SDMs with patients in mainland China” is deviated from your original meaning. Please consider rewrite it as “there are few studies related to ACP involving both SDMs and patients in mainland China.

**Response**: We would like to express our sincere gratitude for your valuable comments and suggestions on our manuscript. We have carefully considered your feedback and made the necessary revisions.

Regarding the statement in the 4th paragraph, we agree with your assessment that the original wording may have deviated from our intended meaning. We have revised the sentence to “there are few studies related to ACP involving both SDMs and patients in mainland China” as you suggested. We believe this revision more accurately conveys our original intention and provides a clearer understanding of the research landscape in this area.

Once again, we thank you for your time and insightful comments. We are confident that these revisions have improved the quality of our manuscript and we look forward to the opportunity to address any further feedback you may have.

2) In the last paragraph of section 2, the elaboration on literature gap closely resembles the discussion section, which sounds duplicated and a preconception before investigation.

**Response**: Thank you for your valuable feedback on our manuscript. We've carefully reviewed your comments and made the appropriate revisions.

We acknowledge your observation regarding the overlap between the elaboration on the literature gap in the last paragraph of Section 2 and the discussion section, which could give an impression of duplication and preconception. In response, we have removed the redundant content from Section 2 to ensure each section has a distinct focus and contributes uniquely to the manuscript.

We believe these revisions have improved the clarity and logical flow of our paper, and we appreciate your time and insightful comments.

3) To address the literature gap, please consider citing the following reference to highlight the complexity of implementing ACP in mainland China.

McMahan, R. D., Tellez, I., & Sudore, R. L. (2021). Deconstructing the Complexities of Advance Care Planning Outcomes: What Do We Know and Where Do We Go? A Scoping Review. Journal of the American Geriatrics Society, 69(1), 234–244. https://doi.org/10.1111/jgs.16801

**Response**: Thank you for your insightful comments and suggestions on our manuscript. We have carefully considered your feedback and made the appropriate revisions.

In response to your suggestion to address the literature gap, we have added the reference you recommended. We believe this addition strengthens our analysis and provides a more comprehensive perspective on the challenges and intricacies of ACP in this specific cultural context.

Thank you again for your time and valuable insights. We are confident that these revisions have improved the quality of our manuscript and we look forward to the opportunity to address any further feedback you may have.

4) Methods: In section 4.1 and 4.2, the handling of missing data has been reported in section 4.4. Please delete it from here.

**Response**: Thank you for your valuable feedback on our manuscript. We have carefully reviewed your comments and made the necessary revisions.

In response to your suggestion regarding the methods section, we have removed the mention of missing data handling from sections 4.1 and 4.2.

We appreciate your guidance in helping us improve the clarity and organization of our manuscript. Thank you again for your time and insights.

5) Results: In section 5.1, according to the figure in table 1, only 8.7% of advanced cancer patients were fully independent.

**Response**: Thank you for bringing this to our attention. In response to your feedback regarding section 5.1, we have revised the description to more accurately reflect the data presented in table 1. The original statement has been updated to emphasize the minority of fully independent patients and clarify the distribution of dependency levels among the majority.

Thank you for your guidance in enhancing the precision of our manuscript.

6) Please clarify if the author would like to emphasize that over 90% of them require ADL support? It sounds more logical to emphasize the caregiver burden in this way than emphasizing that two-thirds of them could take care of themselves.

**Response**: Thank you for your insightful feedback. In response to your suggestion, we have revised the description in section 5.1 to better emphasize the caregiver burden. The original statement has been updated to highlight that over 90% of patients require some level of ADL support, which more accurately reflects the data in table 1 and aligns with the intended emphasis on the challenges faced by caregivers.

Thank you again for your guidance in improving the clarity and logical flow of our manuscript.

7) Discussion: In the 7th paragraph, please consider citing references to support the first two recommendations (involving SDMs in ACP and developing decision aids), especially literature related to the Chinese cultural context:

Yeung, C. C. Y., Ho, K. H. M., & Chan, H. Y. L. (2023). A dyadic advance care planning intervention for people with early-stage dementia and their family caregivers in a community care setting: a feasibility trial. BMC geriatrics, 23(1), 115.

**Response**: Thank you for your valuable suggestions. We have carefully considered your comments and made the appropriate revisions to our manuscript.

In the 7th paragraph of the Discussion section, we have added the reference you suggested to support the first two recommendations. This addition provides a more solid foundation for our recommendations and aligns with the cultural context highlighted in your review.

We believe this revision strengthens our discussion and provides a more comprehensive perspective on the topic. Thank you again for your time and insightful comments.

8) In the 8th pagraph, please consider citing the following reference to support the importance of healthcare provider training:

Mills, J., Kim, S. H., Chan, H. Y., Ho, M. H., Montayre, J., Liu, M. F., & Lin, C. C. (2021). Palliative care education in the Asia Pacific: challenges and progress towards palliative care development. Progress in Palliative Care, 29(5), 251-254.

**Response**: Thank you for your valuable suggestion. In response to your request in the 8th paragraph, we have added the reference to Mills et al. (2021) to support the importance of healthcare provider training. This addition reinforces the significance of education in palliative care and provides a stronger foundation for our discussion.

Thank you again for your guidance in enhancing the scholarly rigor of our manuscript.

Thank you once again for your patience and constructive feedback. We have dedicated significant effort to refining this manuscript in response to the reviewers’ and editors’ concerns. Should any further revisions be required, please do not hesitate to contact us. We sincerely hope that the current version now aligns with the journal’s standards and would be honoured to contribute to PLOS ONE’s scientific community.

Best regards,

Zhihao Han, Jingjing Su, Guiyue Ma, Yong Fang, Linxia Tang, Xiaoqin Ma

Zhejiang Chinese Medical University

[20041028@zcmu.edu.cn](mailto:20041028@zcmu.edu.cn)
